# Author Correction: Identification of PTGR2 inhibitors as a new therapeutic strategy for diabetes and obesity

**DOI:** 10.1038/s44321-025-00228-0

**Published:** 2025-04-03

**Authors:** Yi-Cheng Chang, Meng-Lun Hsieh, Hsiao-Lin Lee, Siow-Wey Hee, Chi-Fon Chang, Hsin-Yung Yen, Yi-An Chen, Yet-Ran Chen, Ya-Wen Chou, Fu-An Li, Yi-Yu Ke, Shih-Yi Chen, Ming-Shiu Hung, Alfur Fu-Hsin Hung, Jing-Yong Huang, Chu-Hsuan Chiu, Shih-Yao Lin, Sheue-Fang Shih, Chih-Neng Hsu, Juey-Jen Hwang, Teng-Kuang Yeh, Ting-Jen Rachel Cheng, Karen Chia-Wen Liao, Daniel Laio, Shu-Wha Lin, Tzu-Yu Chen, Chun-Mei Hu, Ulla Vogel, Daniel Saar, Birthe B Kragelund, Lun Kelvin Tsou, Yu-Hua Tseng, Lee-Ming Chuang

**Affiliations:** 1 Department of Internal Medicine, National Taiwan University Hospital, Taipei, 100225 Taiwan; 2 Graduate Institute of Medical Genomics and Proteomics, National Taiwan University, Taipei, 100225 Taiwan; 3 Institute of Biomedical Sciences, Academia Sinica, Taipei, 115201 Taiwan; 4 Department of Medicinal Chemistry, University of Florida, Gainesville, FL 32610 USA; 5 Genomics Research Center, Academia Sinica, Taipei, 115201 Taiwan; 6 Institute of Biological Chemistry, Academia Sinica, Taipei, 115201 Taiwan; 7 Agricultural Biotechnology Research Center, Academia Sinica, Taipei, 115201 Taiwan; 8 Institute for Drug Evaluation Platform, Development Center for Biotechnology, Taipei, 11571 Taiwan; 9 Institute of Biotechnology and Pharmaceutical Research, National Health Research Institutes, Zhunan, Miaoli County, 35053 Taiwan; 10 Rakuten Medical, San Diego, CA 92121 USA; 11 AltruBio Taiwan R&D Center, Taipei, 114063 Taiwan; 12 Taiwan Liposome Company, Taipei, 11560 Taiwan; 13 Department of Internal Medicine, National Taiwan University Hospital, Yunlin branch, Yunlin, 64041 Taiwan; 14 Biological Sciences Division, University of Chicago, Chicago, IL 60637 USA; 15 Centers of Genomic and Precision Medicine, National Taiwan University, Taipei, 100225 Taiwan; 16 Department of Clinical Laboratory Sciences and Medical Biotechnology, National Taiwan University, Taipei, 10048 Taiwan; 17 National Laboratory Animal Center, National Applied Research Laboratories, Taipei, 11571 Taiwan; 18 National Research Centre for the Working Environment, Lerso Parkallé 105, DK-2100 Copenhagen, Denmark; 19 REPIN, University of Copenhagen, Ole Maaloes Vej 5, DK-2200 Copenhagen N, Denmark; 20 Structural Biology and NMR Laboratory, Department of Biology, University of Copenhagen, Ole Maaløes Vej 5, 2200 Copenhagen, Denmark; 21 The Linderstrøm-Lang Centre for Protein Science, University of Copenhagen, Ole Maaløes Vej 5, DK-2200 Copenhagen, Denmark; 22 Joslin Diabetes Center, Harvard Medical School, Boston, MA 022515 USA

C**orrection to:** *EMBO Molecular Medicine*. 10.1038/s44321-025-00216-4 | Published online 21 March 2025

In this article the affiliation details for Teng-Kuang Yeh were incorrectly given as “AltruBio Taiwan R&D Center, Taipei 114063, Taiwan” but should have been “Institute of Biotechnology and Pharmaceutical Research, National Health Research Institutes, Zhunan, Miaoli County 35053, Taiwan”. The original article has been corrected.

In this article the e-mail address of the corresponding author Lun Kelvin Tsou was incorrect. The original article has been corrected.

