# Supplementary material for: Identification of PTGR2 inhibitors as a new therapeutic strategy for diabetes and obesity
Source: EMBO Mol Med. 2025 Mar 21;17(5):938–66. doi: 10.1038/s44321-025-00216-4 (PMC12081876; doi:10.1038/s44321-025-00216-4)
Supplement: Supplementary file 1 — Appendix [file 44321_2025_216_MOESM1_ESM.pdf]

Appendix Table of Content

1. Appendix Figure S1 Pg 2
2. Appendix Figure S2 Pg 3
3. Appendix Figure S3 Pg 4
4. Appendix Figure S4 Pg 5
5. Appendix Figure S5 Pg 6
6. Appendix Figure S6 Pg 7
7. Appendix Figure S7 Pg 8
8. Appendix Figure S8 Pg 9
9. Appendix Figure S9 Pg 10
10. Appendix Figure S10 Pg 11
11. Appendix Figure S11 Pg 12
12. Appendix Figure S12 Pg 13
13. Appendix Figure S13 Pg 14
14. Appendix Figure S14 Pg 15
15. Appendix Figure S15 Pg 16
16. Appendix Figure S16 Pg 17
17. Appendix Figure S17 Pg 18
18. Appendix Figure S18 Pg 19
19. Appendix Figure S19 Pg 20
20. Appendix Table S1 Pg 22
21. Appendix Table S2 Pg 23
22. Appendix Table S3 Pg 26
23. Appendix Table S4 Pg 27
24. Appendix Table S5 Pg 28

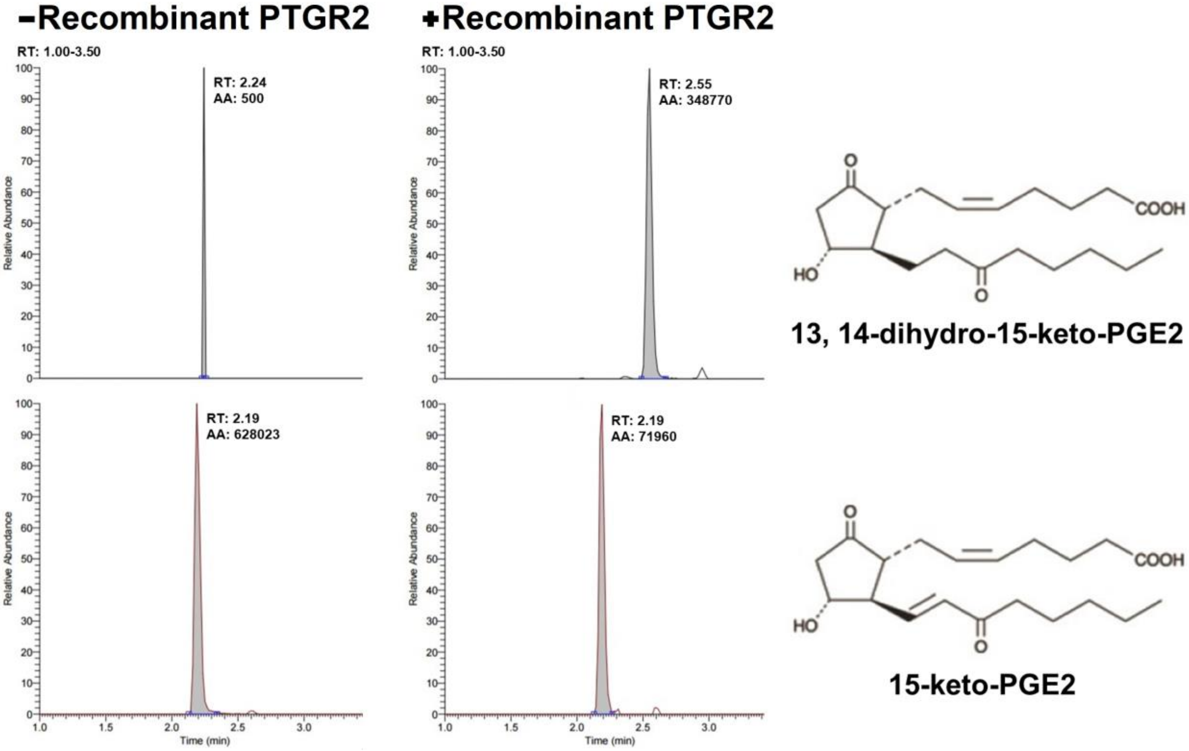

Appendix Figure S1. Orbitrap LC-MS/MS showing 15-keto-PGE2 is converted to 13,14-dihydro-15-keto-PGE2 by recombinant human PTGR2 protein.

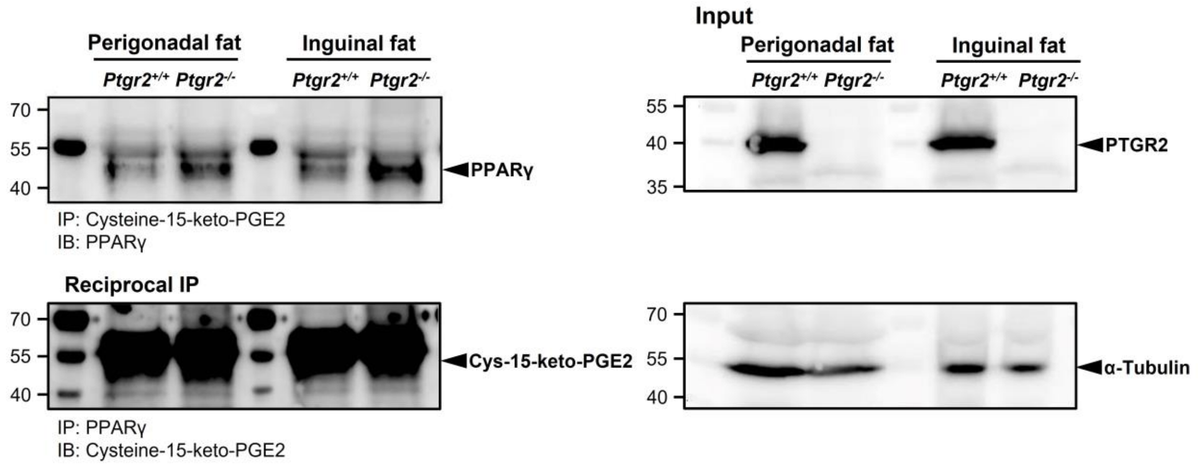

Appendix Figure S2. Reciprocal co-immunoprecipitation between 15-keto-PGE2 and PPARγ in mouse perigonadal white adipose tissues of *Ptgr2*$^{+/+}$ and *Ptgr2*$^{-/-}$ mice. A single band corresponding to murine PPARγ (57 kDa) was detected (upper panel), confirming the covalent binding of PPARγ to 15-keto-PGE2. When protein lysates were reciprocally immunoprecipitated with the anti-PPARγ antibody and immunoblotted with the anti-15-keto-PGE2-cysteine-BSA antibody. However, when protein lysates were reciprocally immunoprecipitated with the anti-PPARγ antibody and immunoblotted with the anti-15-keto-PGE2-cysteine-BSA antibody, the mPPARγ band was obscured by the IgG heavy chain, which is enriched in tissue (lower panel).

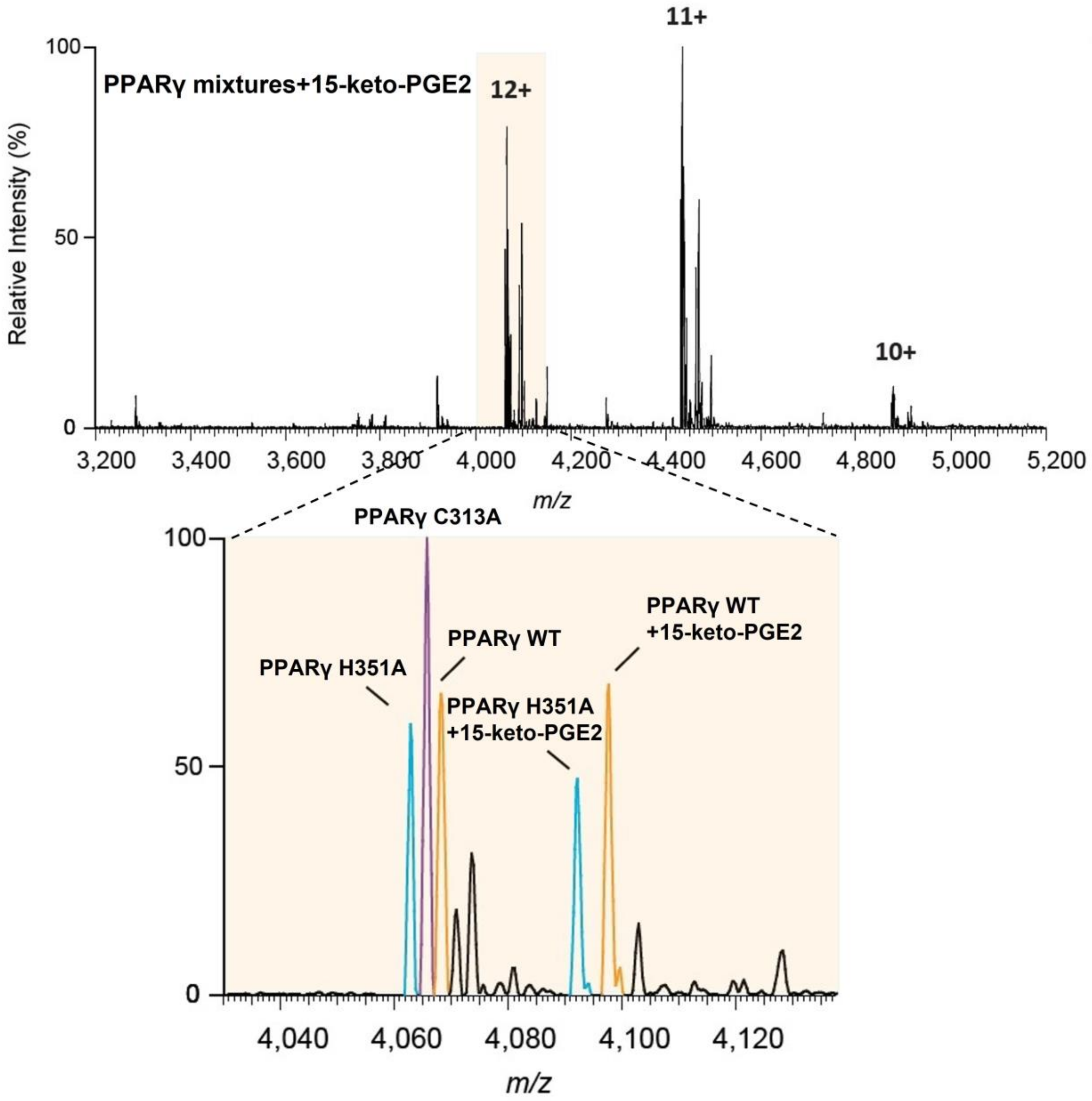

Appendix Figure S3. Examination of 15-keto-PGE2 to wild-type murine PPARγ (mPPARγ) and mutants by native mass spectrometry. The H351A mutants, C313A mutants and wild-type mPPARγ are denoted in blue, magenta and orange respectively. The 15-keto-PGE2 was analyzed by measuring the increased mass in individual proteins. The binding of 15-keto-PGE2 was analyzed at the indicated charge state (12+), highlighted in the vanilla background.

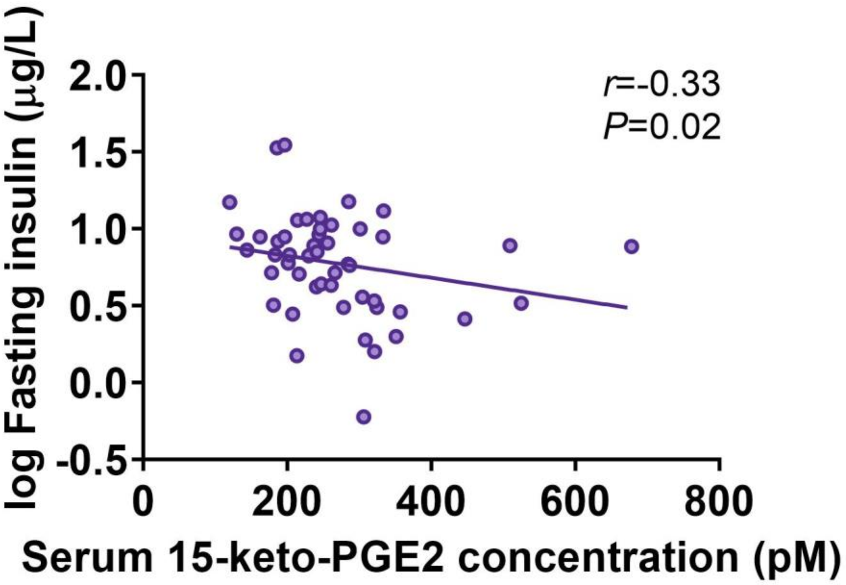

Appendix Figure S4. The inverse association between serum 15-keto-PGE2 levels and log fasting insulin levels in 50 non-diabetic human subjects.

Data information: Statistical significance was calculated by Spearman's correlation.

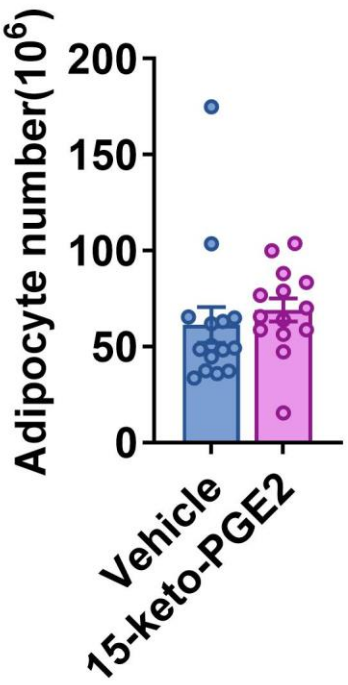

Appendix Figure S5. The number of adipocytes of perigonadal fat in 15-keto-PGE2-treated mice compared with control mice (n=15:14 mice with at least 3 images measured for each mouse).

Data information: Data are presented as mean and standard error (S.E.M.). Statistical significance was calculated by two-sample independent *t*-test.

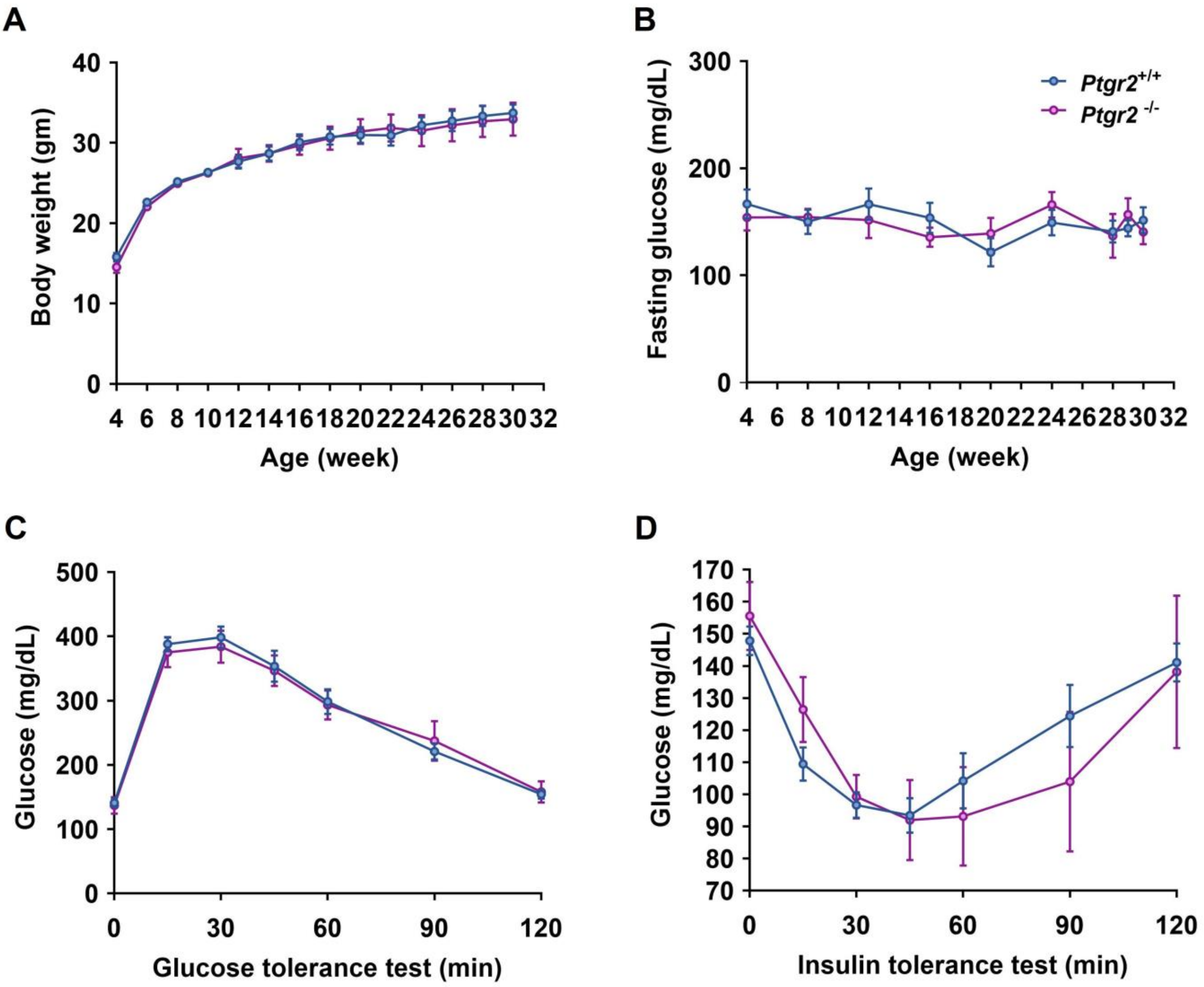

Appendix Figure S6. Body weight (n=44:42 mice) (A), fasting glucose (n=25:26 mice) (B), glycemic levels during intraperitoneal glucose tolerance test a (n=25:26 mice) (C) and insulin tolerance test of *Prgr2*$^{-/-}$ and *Prgr2*$^{+/+}$ mice on chow diet (n=25:26 mice) (D) at the age of 30 weeks.

Data information: Data are presented as mean and standard error (S.E.M.). Statistical significance was calculated by two-sample independent *t*-test.

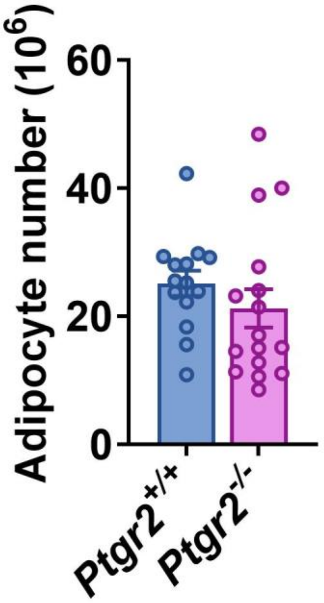

Appendix Figure S7. The number of adipocytes of in the perigonadal fat of *Ptgr2*$^{-/-}$mice compared with control mice (n=14:16 mice with at least 3 images measured for each mouse).

Data information: Data are presented as mean and standard error (S.E.M.). Statistical significance was calculated by two-sample independent *t*-test.

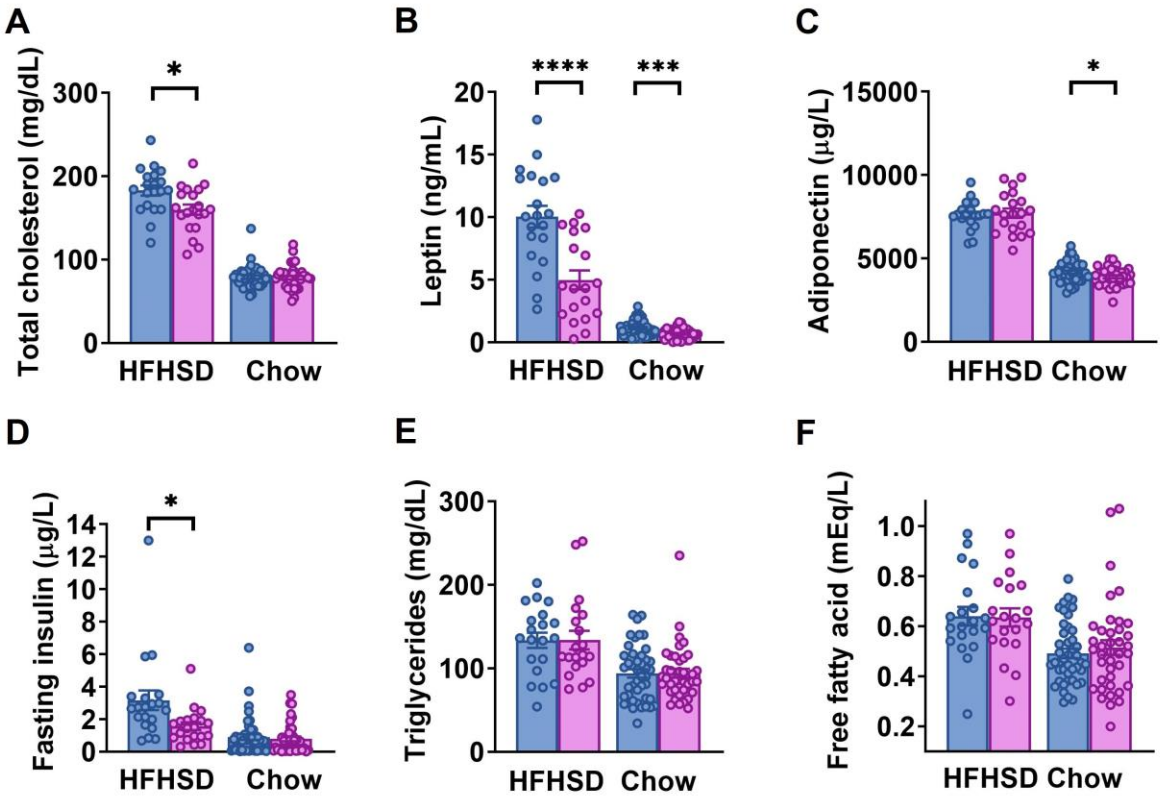

Appendix Figure S8. (A) Total cholesterol (**P*=0.0112), (B) leptin (*****P*<0.0001, ****P*=0.0001), (C) adiponectin (**P*=0.0189), (D) fasting insulin (**P*=0.0189), (E) triglycerides and (F) free fatty acid of *Ptgr2*$^{-/-}$ and *Ptgr2*$^{+/+}$ mice on chow (n=44:38 mice) or high-fat high-sucrose diet (HFHSD) (n=20:20 mice) at the age of 30 weeks. The blue dot represents *Ptgr2*$^{+/+}$ mice and the purple dot represents *Ptgr2*$^{-/-}$ mice.

Data information: Data are presented as mean and standard error (S.E.M.). Statistical significance was calculated by two-sample independent t-test. * $p < 0.05$, *** $p < 0.001$, **** $p < 0.0001$.

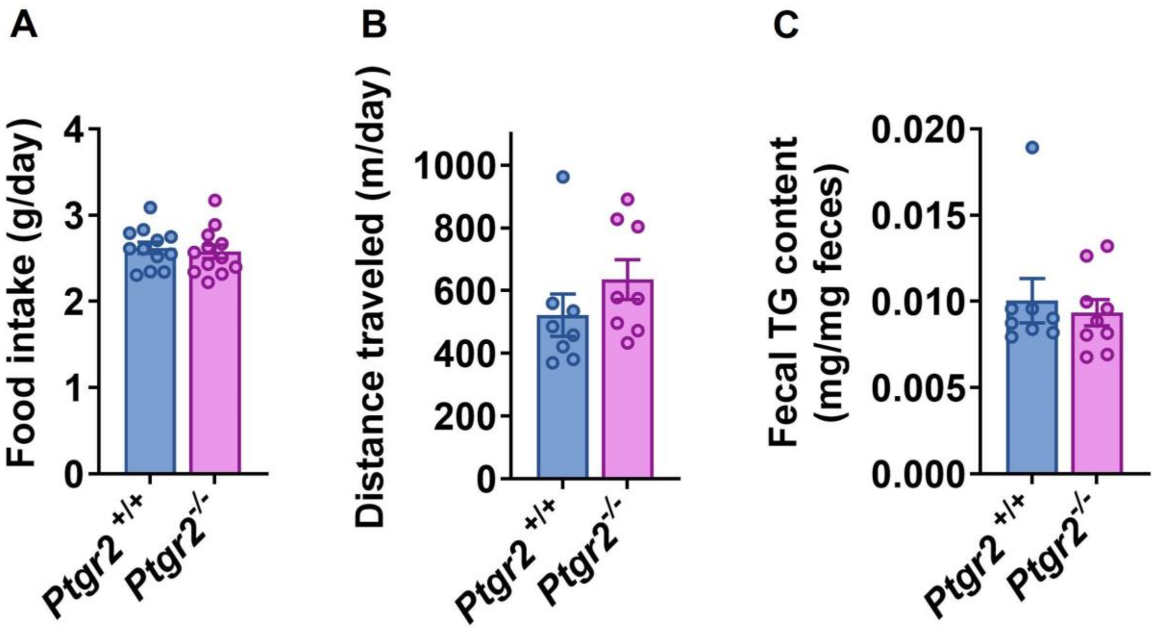

Appendix Figure S9. (A) Food intake (n=12:12 mice), (B) distance traveled (n=8:8 mice), (C) fecal triglyceride content (n=8:9 mice) of *Ptgr2*$^{-/-}$ and *Ptgr2* $^{+/+}$ mice on HFHSD.

Data information: Data are presented as mean and standard error (S.E.M.). Statistical significance was calculated by two-sample independent *t*-test.

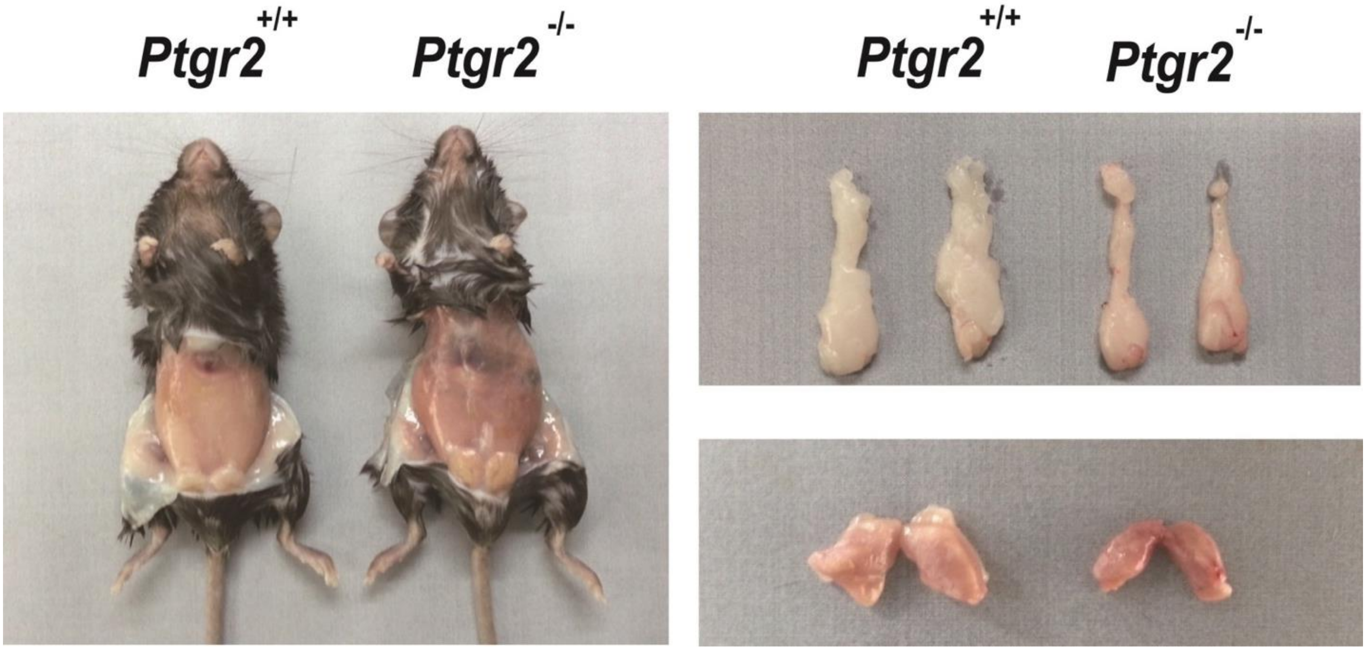

Appendix Figure S10. Gross appearance (left panel), perigonadal fat (right upper panel), and brown fat (right lower panel) of *Ptgr2*$^{-/-}$ mice and *Ptgr2*$^{+/+}$ mice on high-fat high-sucrose diet.

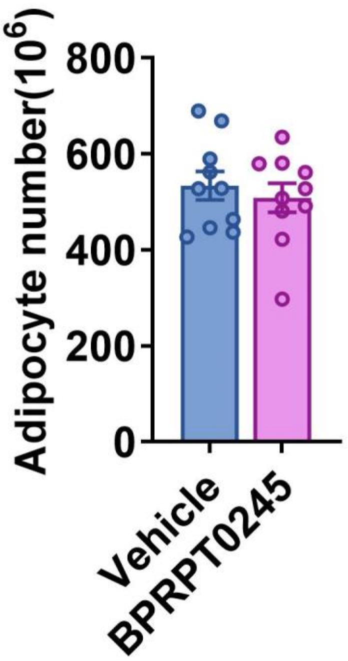

Appendix Figure S11. The adipocyte number of perigonadal fat in mice receiving BPRPT0245 compared to vehicle (n=10:10 mice with at least 3 images measured for each mouse)**.**

Data information: Data are presented as mean and standard error (S.E.M.). Statistical significance was calculated by two-sample independent *t*-test.

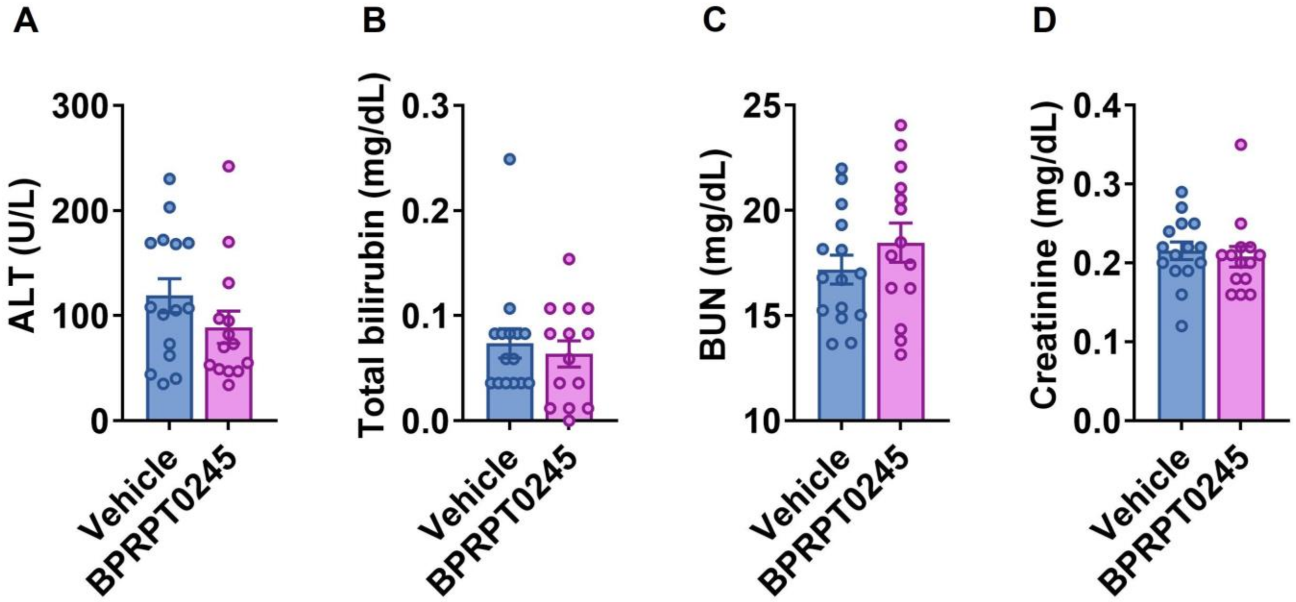

Appendix Figure S12. Serum (A) alanine aminotransferase (ALT) (B) total bilirubin (C) blood urea nitrogen (BUN) and (D) creatinine levels of C57BL6/J receiving vehicle and BPRPT0245 (100mg/kg/day) (n=15:14 mice).

Data information: All data are presented as mean and standard error (S.E.M.). Statistical significance was calculated by two-sample independent *t*-test.

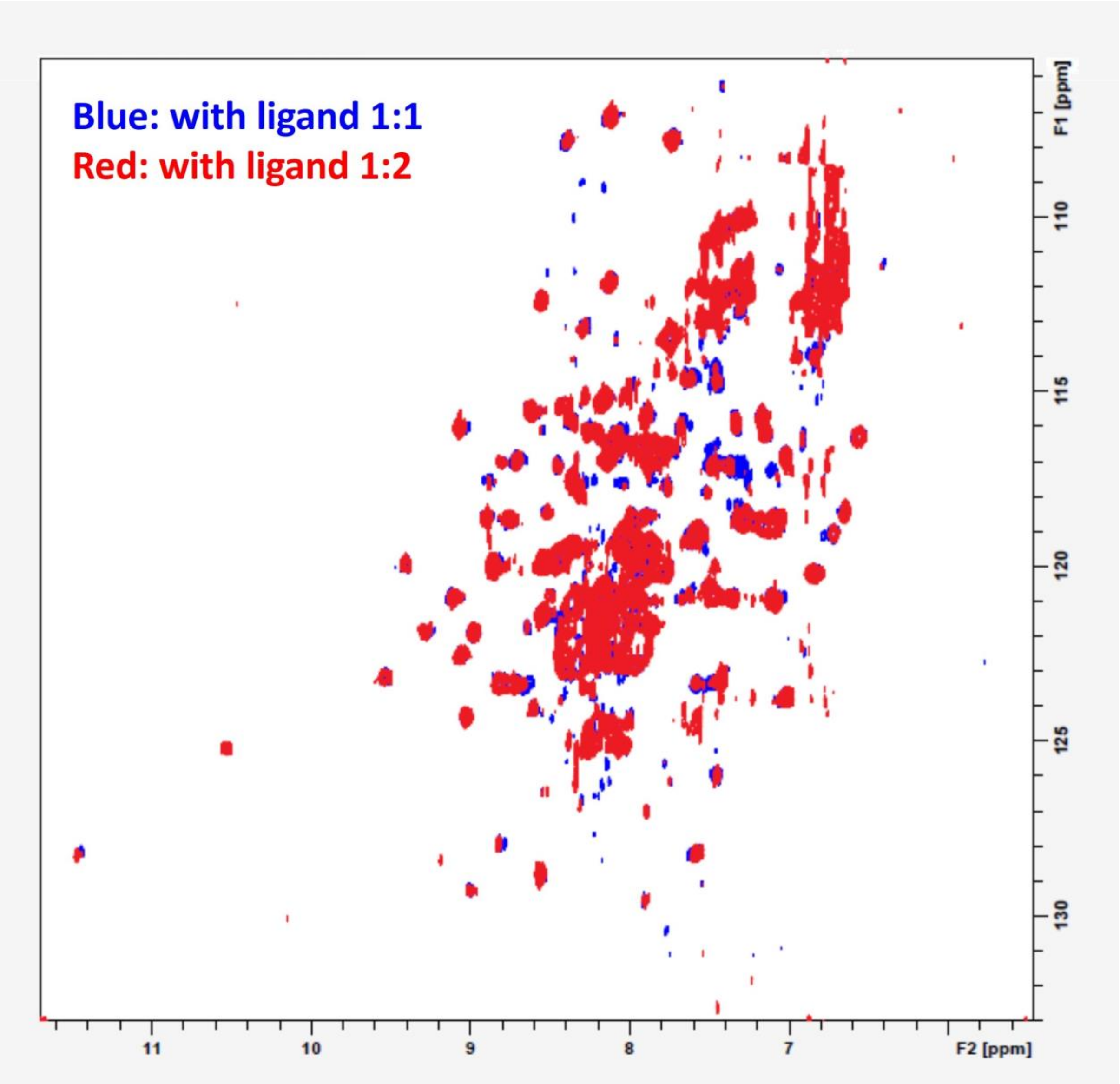

Appendix Figure S13. Comparison of the 2D ^{1}H-^{15}N-TROSY-HSQC NMR spectra NMR spectra of 15-keto-PGE2-bound human PPARγ ligand binding domain at two different molar ratios of 15-keto-PGE2 (1:1 vs. 2:1).

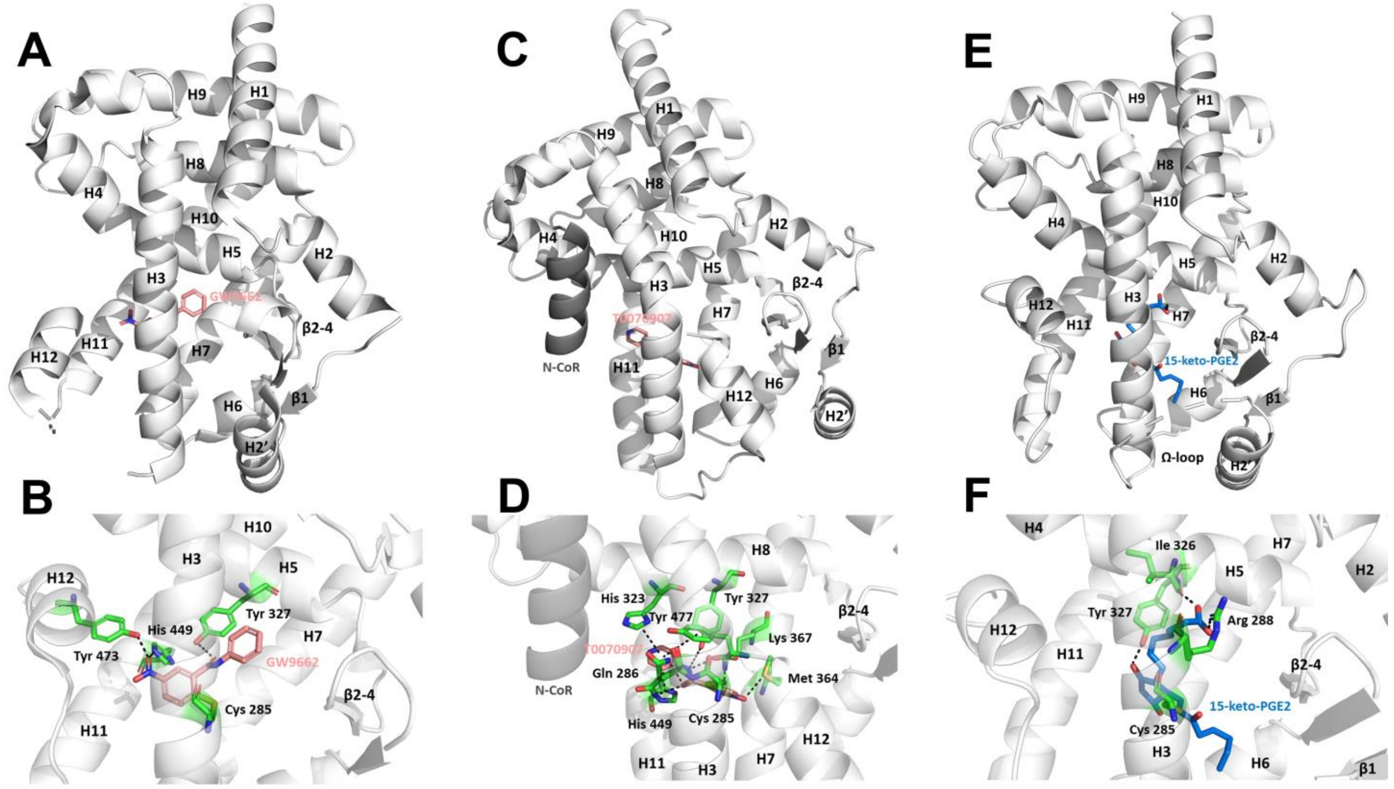

Appendix Figure S14. (A)(B) X-ray crystallography showed covalent PPARγ antagonist GW9662 forms a covalent bond with Cys285 (helix 3) and hydrogen bonds with Tyr327 (helix 5), His449 (helix 10) and Tyr473 at helix 12 of human PPARγ (hPPARγ) ligand binding domain (LBD) (PDB: 3B0R chain B). (C)(D) The recruitment of co-repressors such as NCoR1 by T0090907 leads to a unique transcriptionally repressive conformation of the hPPARγ LBD. In this unique conformation, helix 12 was turned into a pocket flanked by helix 3, helix 2', and β-sheets. This helical turn of helix 12 leaves the remaining AF-2 space exposed for more co-repressors binding. In this repressive conformation, an extensive network of interactions between T0070907 and nearby residues to lock helix 12 within the orthosteric ligand-binding pocket (PDB: 6ONI). (E)(F) Molecular docking showed that 15-keto-PGE2 forms a covalent bond with Cys285 and hydrogen bonds with Tyr327 (helix 5), Arg288 (helix 3) and Ile326 (helix 5) of hPPARγ LBD.

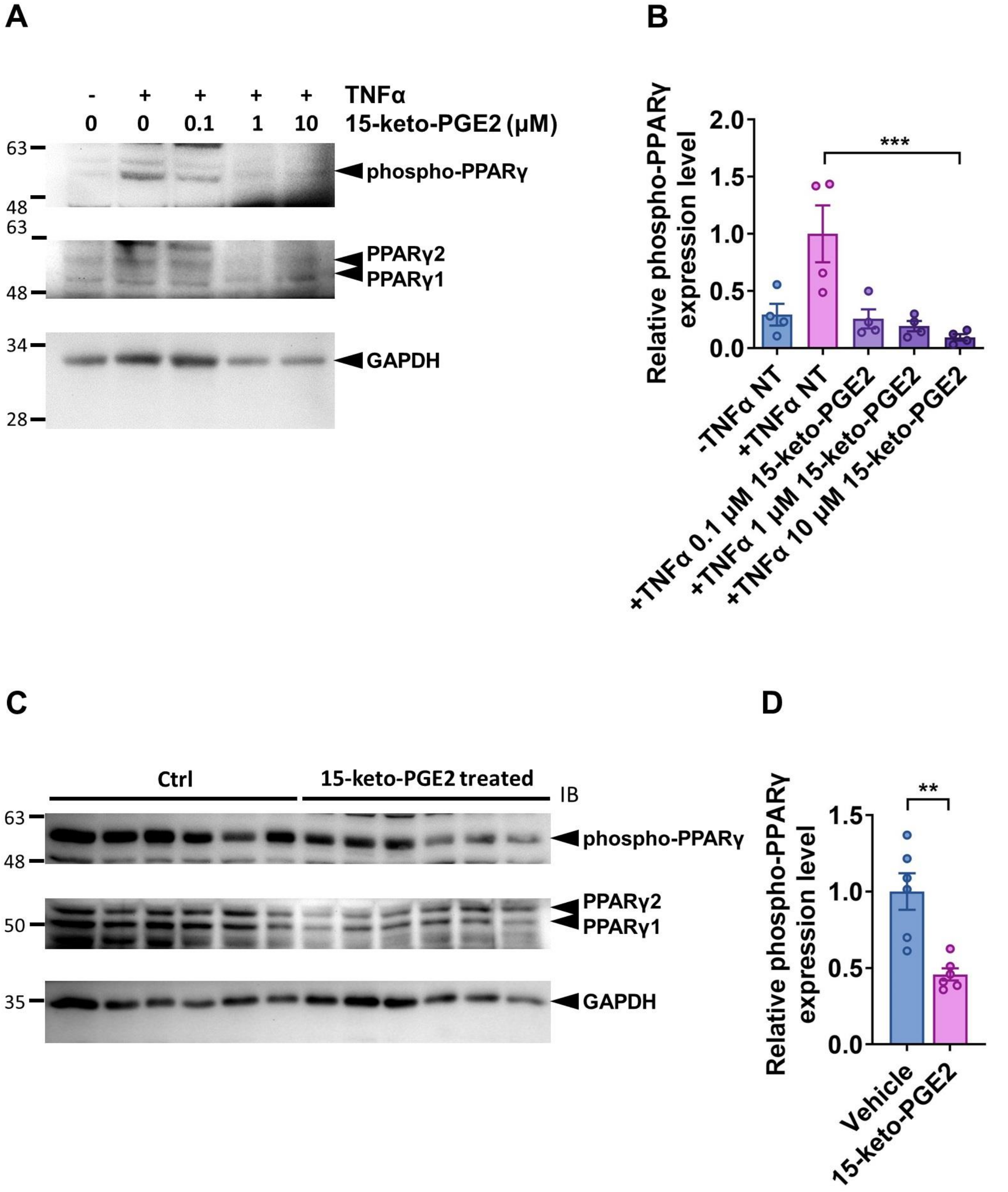

Appendix Figure S15. The effect of 15-keto-PGE2 on TNFα-induced phosphorylation of murine PPARγ Ser273 in both (A, B) cultured adipocytes (****P*=0.0006; n=4 per group, 4 biological replicates with 1 technical replicate) and (C, D) perigonadal adipose tissue (***P*=0.0016; n=6:6 mice) of mice.

Data information: Data are presented as mean and standard error (S.E.M.). Statistical significance was calculated by one-way analyses of variance (ANOVA) with Tukey's post hoc test in (B) and two-sample independent *t*-test in (D). ** $P < 0.01$, *** $P < 0.001$.

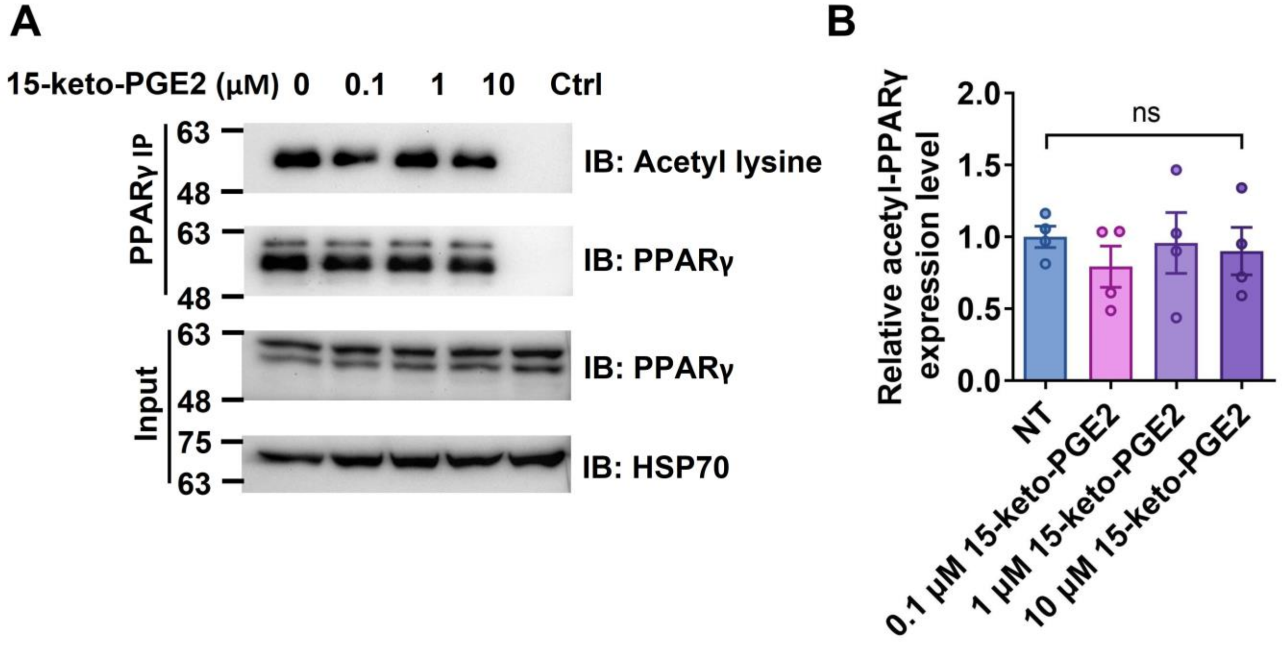

Appendix Figure S16. (A, B) The effect 15-keto-PGE2 on lysine acetylation of murine PPARγ in cultured adipocytes (n=4 per group, 4 biological replicates with 1 technical replicate). NT: non-treatment; ns: not significant.

Data information: Data are presented as mean and standard error (S.E.M.). Statistical significance was calculated by one-way analyses of variance (ANOVA) with Tukey's post hoc test in (B).

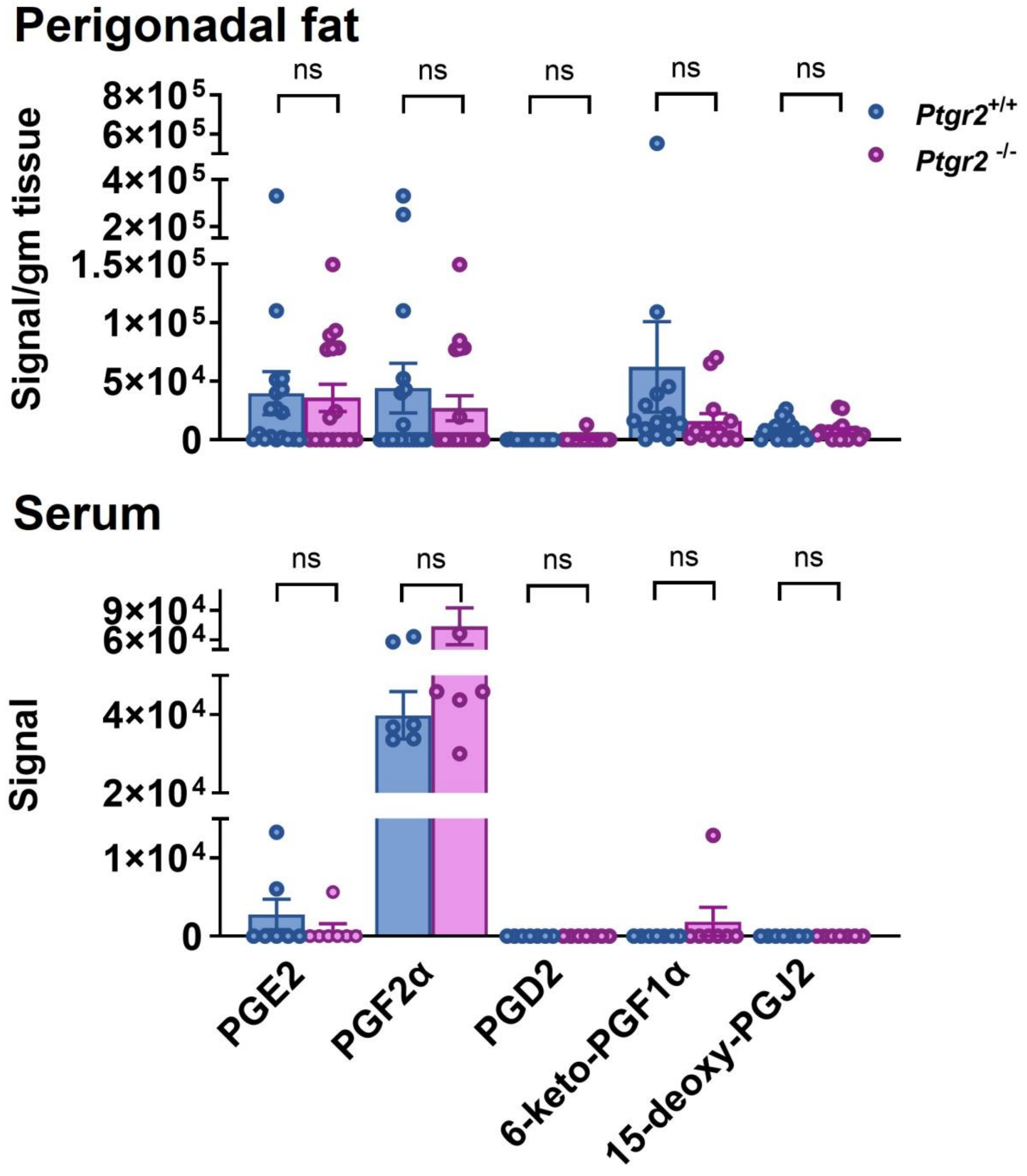

Appendix Figure S17 Levels of PGE2, PGF2α, PGD2, 15-deoxy-PGJ2, and 6-keto-PGF1α in the perigonadal fat (n=14:13 mice) and serum (n=7:7 mice) of *Ptgr2*$^{+/+}$ and *Ptgr2*$^{-/-}$ mice.

Data information: All data are presented as mean and standard error (S.E.M.). Statistical significance was calculated by two-sample independent *t*-test. ns: not significant.

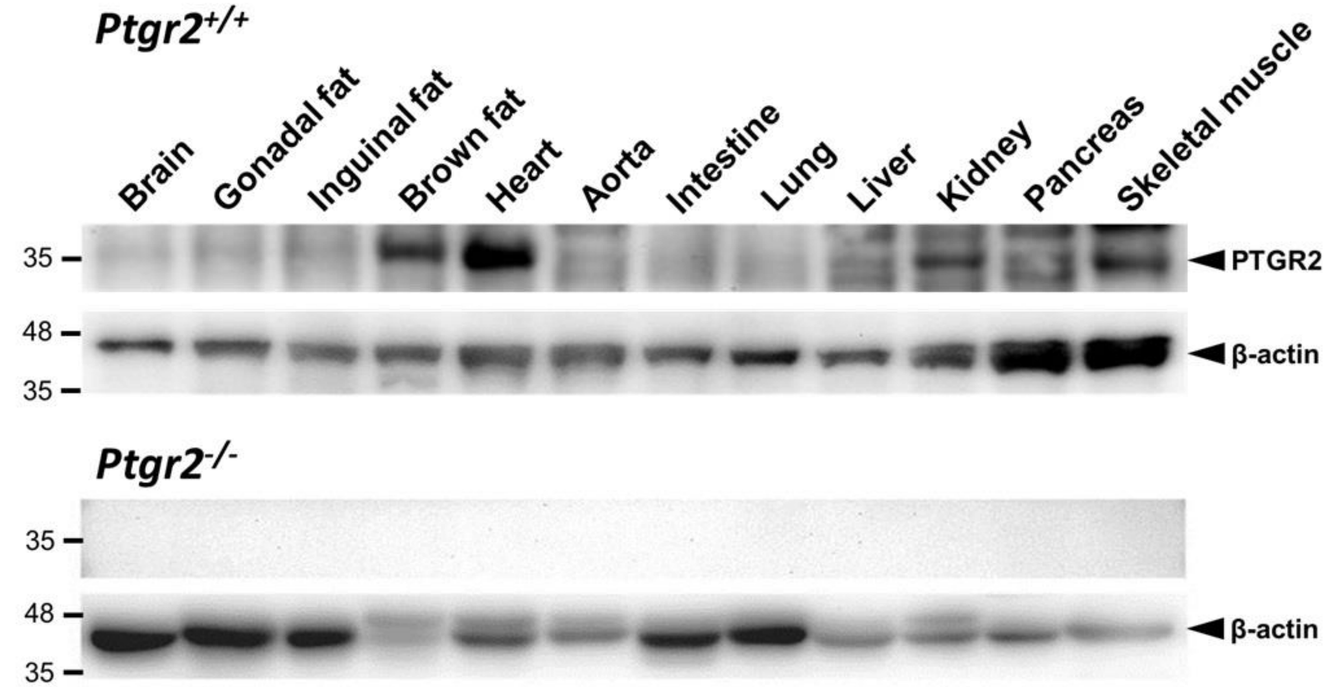

Appendix Figure S18. PTGR2 expression in various tissues of *Ptgr2*$^{+/+}$ (upper panel) and *Ptgr2*$^{-/-}$ (lower panel) mice.

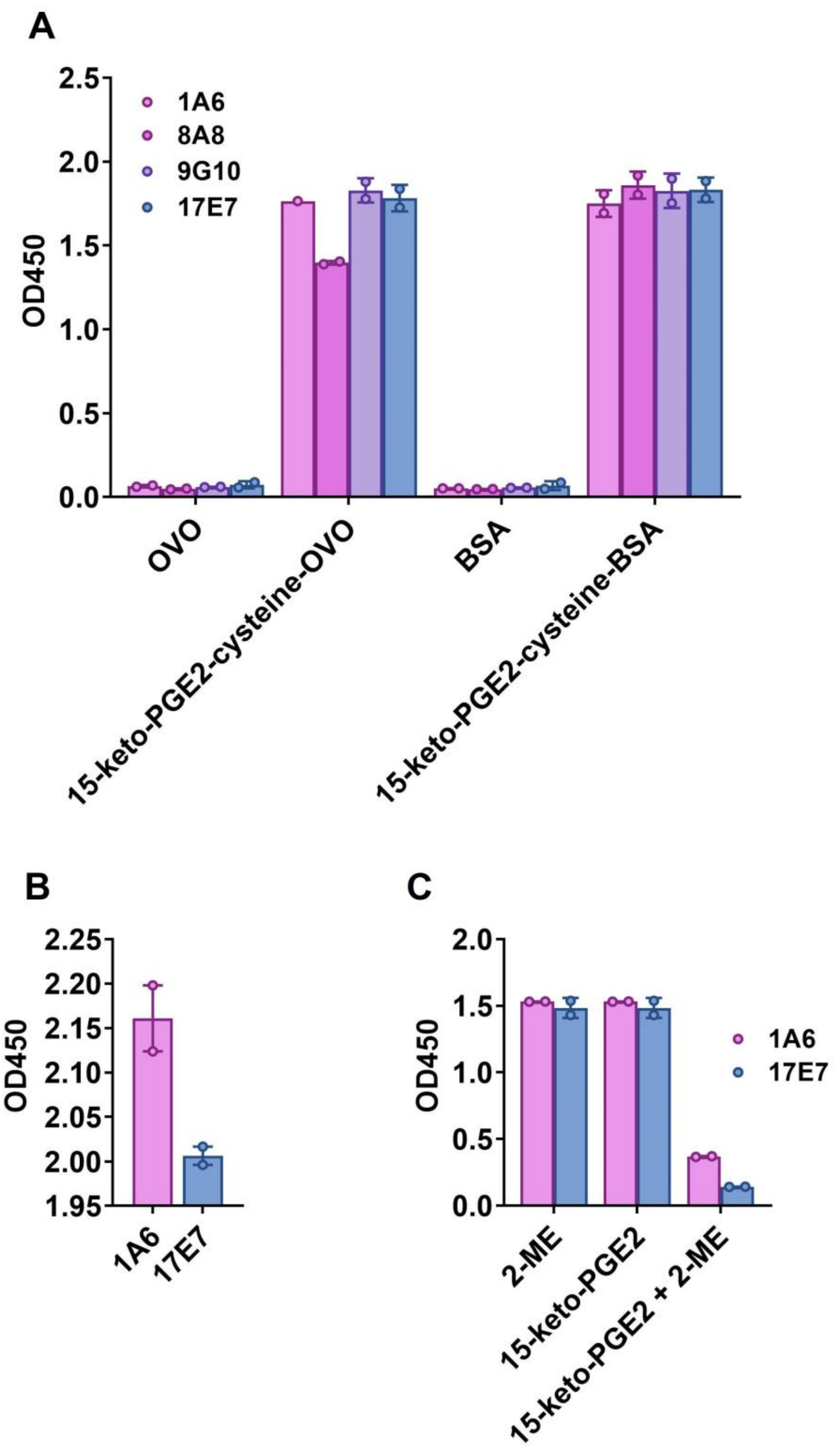

Appendix Figure S19 Four anti-cysteine-coupled 15-keto-PGE2 hybridoma clones were identified. (A) Clone 1A6 and 17E7 have superior specificity and (B) react only to cysteine-coupled 15-keto-PGE2. (C) The specificity of monoclonal antibodies was examined using ELISA assays. Hybridoma culture supernatants were tested for the reactivity to different antigens coated onto micro-wells. To further test specificity, 10 nM 15-keto-PGE2 was reduced with 2-mercaptoethanol (2-ME). Reduced 15-keto-PGE2 (10 nM), 15-keto-PGE2 (10 nM), and 2-ME (20 nM) were mixed with 1 nM purified antibodies in PBS containing 1% BSA,

respectively, and incubated at room temperature for 30 min. The antibody mixtures were then added into wells coated with 15-keto-PGE2-cysteine-BSA for assays (n=2 technical replicates).

Appendix Table S1. Pharmacokinetic study of BPRPT0245 in mice.

Mouse IV (dose: 2mg/kg)				Mouse PO (dose: 10 mg/kg)				
$T_{1/2}$ (hr)	Cl (mL/min/kg)	Vss (L/kg)	AUC (0-inf) (ng/mL*hr)	$T_{1/2}$ (hr)	C_{max} (ng/ml)	T_{max} (hr)	AUC (0-inf) (ng/mL*hr)	F(%)
2	79.5	5	480	2.5	398	0.4	725	30.2

$T_{1/2}$: half-life, Cl: clearance, Vss: steady-state volume of distribution, AUC: area under curve, C_{max}: maximum serum concentration, T_{max}: time take to reach C_{max}, F(%): bioavailability. (Unpublished data)

Appendix Table S2. Pathological examination of major organs between mice receiving BPRPT0245 and vehicles

**Group**	**Control**				
**Animal ID**	**C1**	**C2**	**C3**	**C4**	**C5**
**Brain, Forebrain**	X	X	X	X	X
**Brain, Midbrain**	X	X	X	X	X
**Brain, Cerebellum and Pons**	X	X	X	X	X
**Heart**					
Inflammatory cell infiltration/Cardiomyopathy	1	1	1	1	1
Vacuolation, cardiomyocyte	1	1	1	-	1
**Lung**					
Inflammatory cell infiltration, perivascular	2	2	2	1	X
Alveolar epithelium hyperplasia	-	-	2	-	X
Chronic inflammation, focal	-	1	-	1	X
**Liver**					
Steatosis	3	3	3	3	4
Inflammation	2	1	2	2	2
Lymphocytic cell infiltration, perivascular	-	2	2	-	1
Hepatocellular adenoma, multiple	-	-	+	-	-
**Kidney**					
Tubular degeneration/regeneration with inflammatory cell infiltration	2	2	2	2	2
Inflammatory cell infiltration, peripelvic /perivacscular	2	2	2	2	2
Hyaline cast, renal tubule	1	-	1	1	1
Cytoplasmic vacuolation, renal tubule	2	2	2	2	2
Mineralization, renal tubule	1	-	1	-	-
**Pancreas**					
Atrophy and accumulation adipocyte, acinar cell	-	-	4	-	-
Pancreatic islets hyperplasia	-	-	-	2	-
Lymphocytic infiltration, perivascular	1	1	1	2	-
Fat infiltration, exocrine	2	-	-	2	1
**Brown fat (largely in dorsal intrascapular region)**					
Increased adipocyte size	+	-	+	+	+
**White fat (Perigonadal fat)**					
Inflammatory cell infiltration	1	-	1	-	1
**Bone marrow, Sternum/ Femur**	X	X			
Mineralization, focal			2	-	-

Cellularity, decreased, bone marrow			-	2	1

**Group**	**Test**			
**Animal ID**	**T1**	**T2**	**T2**	**T4**
**Brain, Forebrain**	X	X	X	X
**Brain, Midbrain**	X	X	X	X
**Brain, Cerebellum and Pons**	X	X	X	X
**Heart**				
Inflammatory cell infiltration/Cardiomyopathy	2	1	2	2
Vacuolation, cardiomyocyte	-	-	-	2
**Lung**				
Inflammatory cell infiltration, perivascular	1	1	2	2
Alveolar epithelium hyperplasia	2	-	-	-
Chronic inflammation, focal	1	-	1	-
**Liver**				
Steatosis	2	4	2	4
Inflammation	1	2	1	2
Lymphocytic cell infiltration, perivascular	1	1	2	2
Hepatocellular adenoma, multiple	-	-	-	-
**Kidney**				
Tubular degeneration/regeneration with inflammatory cell infiltration	2	2	2	2
Inflammatory cell infiltration, peripelvic /perivacscular	2	1	2	2
Hyaline cast, renal tubule	1	-	-	-
Cytoplasmic vacuolation, renal tubule	2	-	2	2
Mineralization, renal tubule	1	-	-	-
**Pancreas**				
Atrophy and accumulation adipocyte, acinar cell	-	-	-	-
Pancreatic islets hyperplasia	1	1	-	-
Lymphocytic infiltration, perivascular	-	-	2	2
Fat infiltration, exocrine	2	1	2	1
**Brown fat (largely in dorsal intrascapular region)**				
Increased adipocyte size	+	+	+	+
**White fat (Perigonadal fat)**	X	X		
Inflammatory cell infiltration			1	2
**Bone marrow, Sternum/ Femur**	X		X	X
Mineralization, focal		-		

Cellularity, decreased, bone marrow		-		
Infiltration, lymphocyte		1		

+ = Present; - =Not present; X=Not remarkable lesion. Degrees of lesions were graded histopathologically from one to five depending on severity (1 = minimal (< 1%); 2 = mild (1–25%); 3 = moderate (26–50%); 4 = moderately severe (51–75%); 5 = severe/high (76–100%).

Gross examinations were summarized as follows: (1) smaller than usual size of the pancreas - The lesion was observed in control mouse (ID: C3). Histopathologically, the pancreas showed exocrine atrophy and fat infiltration. (2) pale discoloration of the liver - The lesion was observed in 2 mice of test group and 5 mice of control group. Histopathologically, the liver showed steatosis and inflammation. (3) small hepatic nodules (<3 mm) of the liver - Histopathologically, the liver showed hepatocellular adenoma. The microscopic alterations were presented in Supplementary Table 5 as follows: 1. Histopathology changes related to the experimental outcomes (non-spontaneous lesions)(1). steatosis with inflammation of the liver (fatty change of liver). (2). cytoplasmic vacuolation of the renal tubule. (3). cardiomyocyte vacuolation. (4). fatty infiltration of exocrine pancreas (adipocyte accumulation). (5). diffuse atrophy of exocrine acini and replacement by adipose tissue.(6). Increase adipocyte size of the brown fat (largely in dorsal intrascapular region). 2. Lesions related to the spontaneous lesions. Several microscopic lesions were observed in organs of the submitted mice, but they were considered to be spontaneous developmental and aging lesions or congenital abnormality. It consisted of: cardiomyopathy/inflammatory cell infiltration, inflammatory cell infiltration at perivascular area in the lung, alveolar epithelium hyperplasia, focal chronic inflammation of the lung, lymphocytic cell infiltration at perivascular area in the liver, hepatocellular adenoma, tubular degeneration/regeneration with inflammatory cell infiltration of the kidney, inflammatory cell infiltration in peripelvic area, renal tubular focal mineralization of medulla, hyaline cast formation of the renal tubule, pancreatic islets hyperplasia, lymphocytic
infiltration at perivascular area in the pancreas, focal mineralization of the bone marrow
(femur) and decreased cellularity of the bone marrow (femur).

In conclusion, several microscopic changes in various tissues of treated and control animals were considered incidental spontaneous and ageing changes, and these were not related to the
test article administration.

Appendix Table S3. RT-qPCR primer sequences

Gene	Gene ID	Forward primer (5'-3')	Reverse primer (5'-3')
Glut4 (Slc2a4)	20528	ACATACCTGACAGGGCAAGG	CGCCCTTAGTTGGTCAGAAG
Irs2	384783	CACAATTCCAAGCGCCACAA	CATCACCTCCTCCCAGGGTA
Sorbs1	20411	TGGACCAGACAAAGACATGGA	TTCCCCTGTAGGAAGACGAGG
Cd36	12491	ATGGGCTGTGATCGGAACTG	GTCTTCCCAATAAGCATGTCTCC
Acs	60525	GCTGCCGACGGGATCAG	TCCAGACACATTGAGCATGTCAT
Cebpα	12606	GAACAGCAACGAGTACCGGGTA	GCCATGGCCTTGACCAAGGAG
Adipq	11450	TCCTGGAGAGAAGGGAGAGAAAG	CCCTTCAGCTCCTGTCATTCC
Ppia	268373	GCATACGGGTCCTGGCATCTTGTCC	ATGGTGATCTTCTTGCTGGTCTTGC

Appendix Table S4. Primers for site-directed mutagenesis

	**Sequence (5’-3’)**
Forward	CATCCGAATTTTTCAAGGGGCCCAGTTTCGATCCGTAGAA
Reverse	TTCTACGGATCGAAACTGGGCCCCTTGAAAAATTCGGATG

Appendix Table S5. Sequence of sgRNAs

	**Sequence (5'-3')**
sgRNA1	CGGCTTCTACGGATCGAAAC
sgRNA2	GCACCCTTGAAAAATTCGGA
