# Supplementary figures and images for: Identification of PTGR2 inhibitors as a new therapeutic strategy for diabetes and obesity
Source: EMBO Mol Med. 2025 Mar 21;17(5):938–66. doi: 10.1038/s44321-025-00216-4 (PMC12081876; doi:10.1038/s44321-025-00216-4)

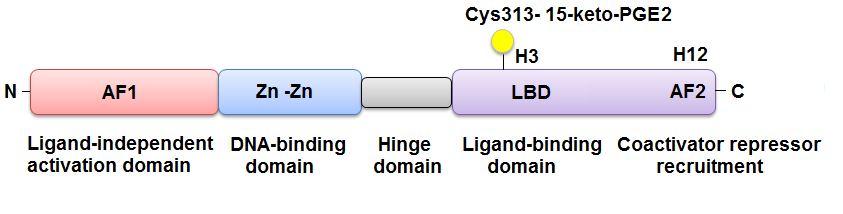

Supplement: Supplementary file 3 — Source data Fig. 1 [file 44321_2025_216_MOESM3_ESM.zip › Figure 1/1Q.tif]

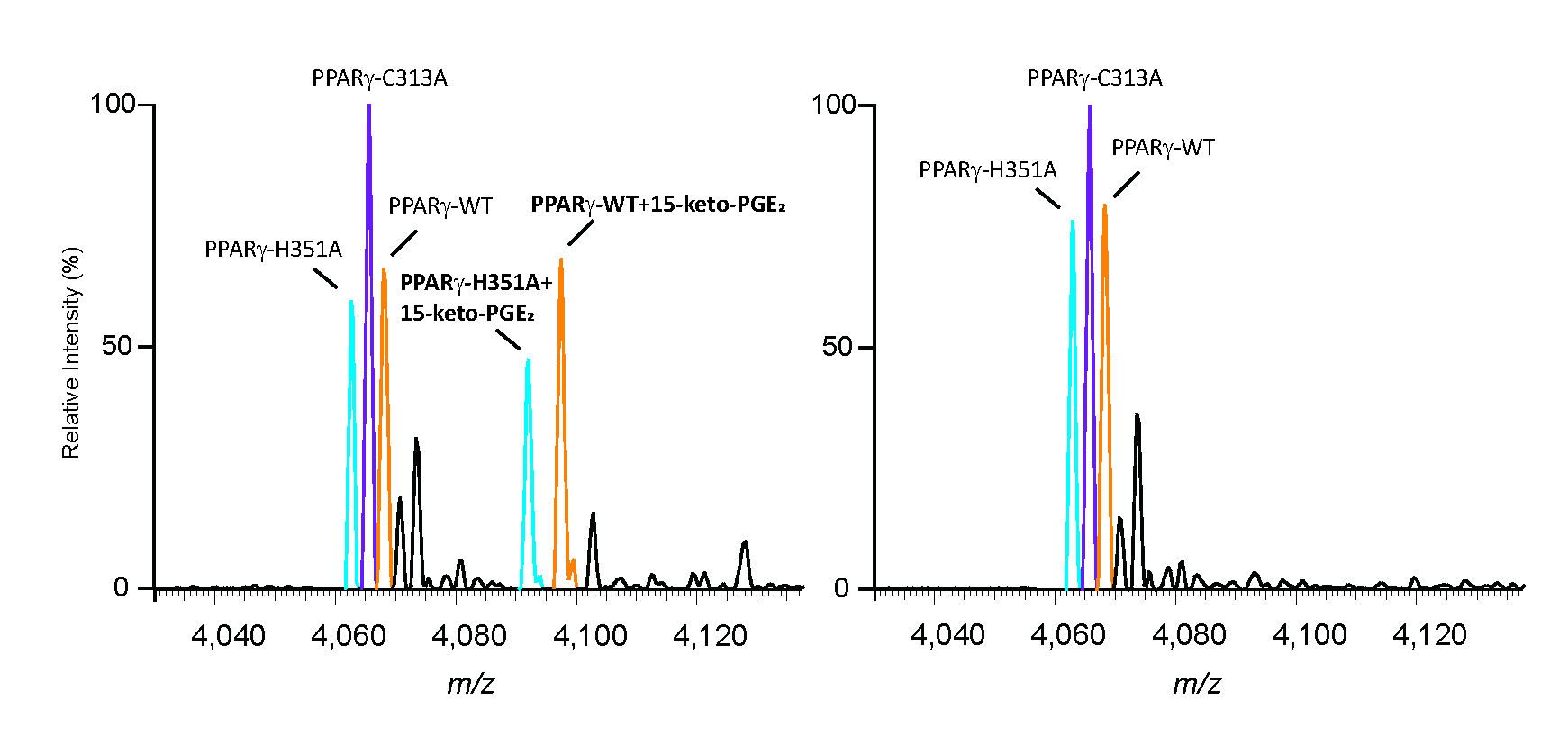

Supplement: Supplementary file 3 — Source data Fig. 1 [file 44321_2025_216_MOESM3_ESM.zip › Figure 1/Figure 1N.tif]

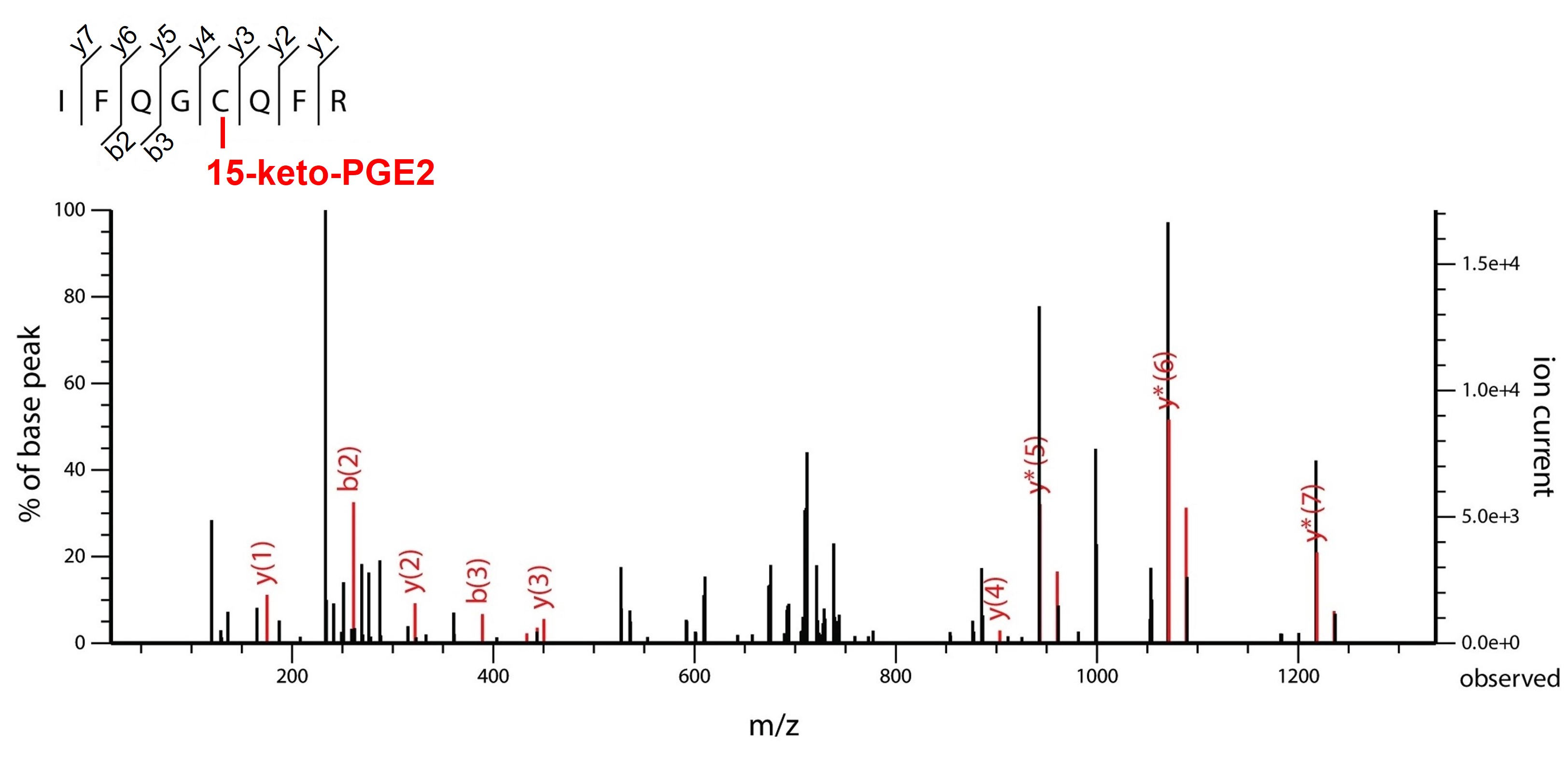

Supplement: Supplementary file 3 — Source data Fig. 1 [file 44321_2025_216_MOESM3_ESM.zip › Figure 1/1K.jpg]

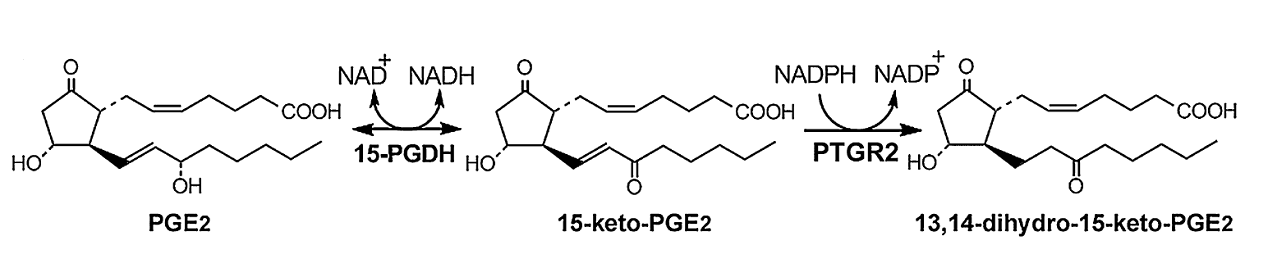

Supplement: Supplementary file 3 — Source data Fig. 1 [file 44321_2025_216_MOESM3_ESM.zip › Figure 1/Figure 1A.tif]

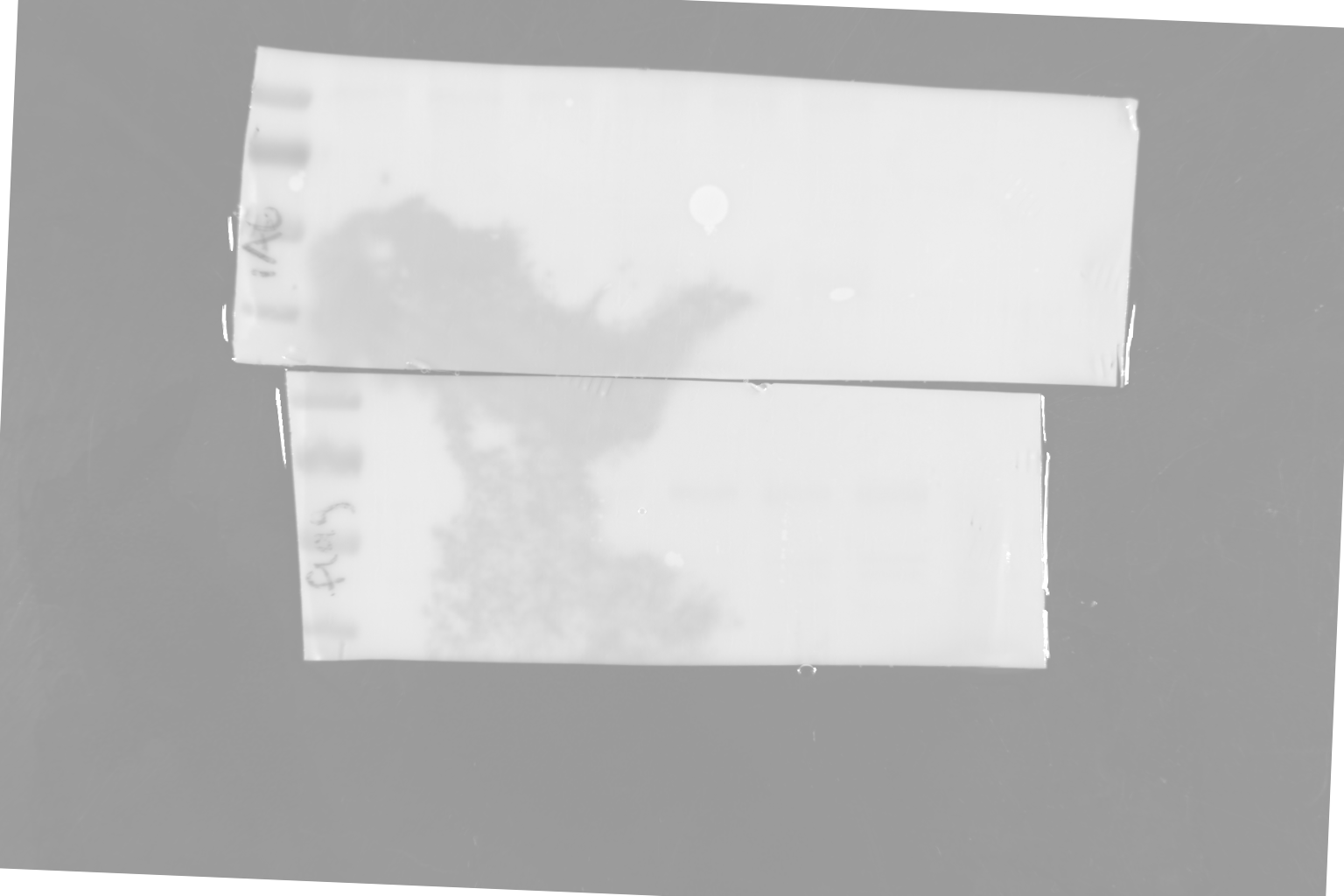

Supplement: Supplementary file 3 — Source data Fig. 1 [file 44321_2025_216_MOESM3_ESM.zip › Figure 1/Figure 1L/Figure 1L left panel blank and markers.tif]

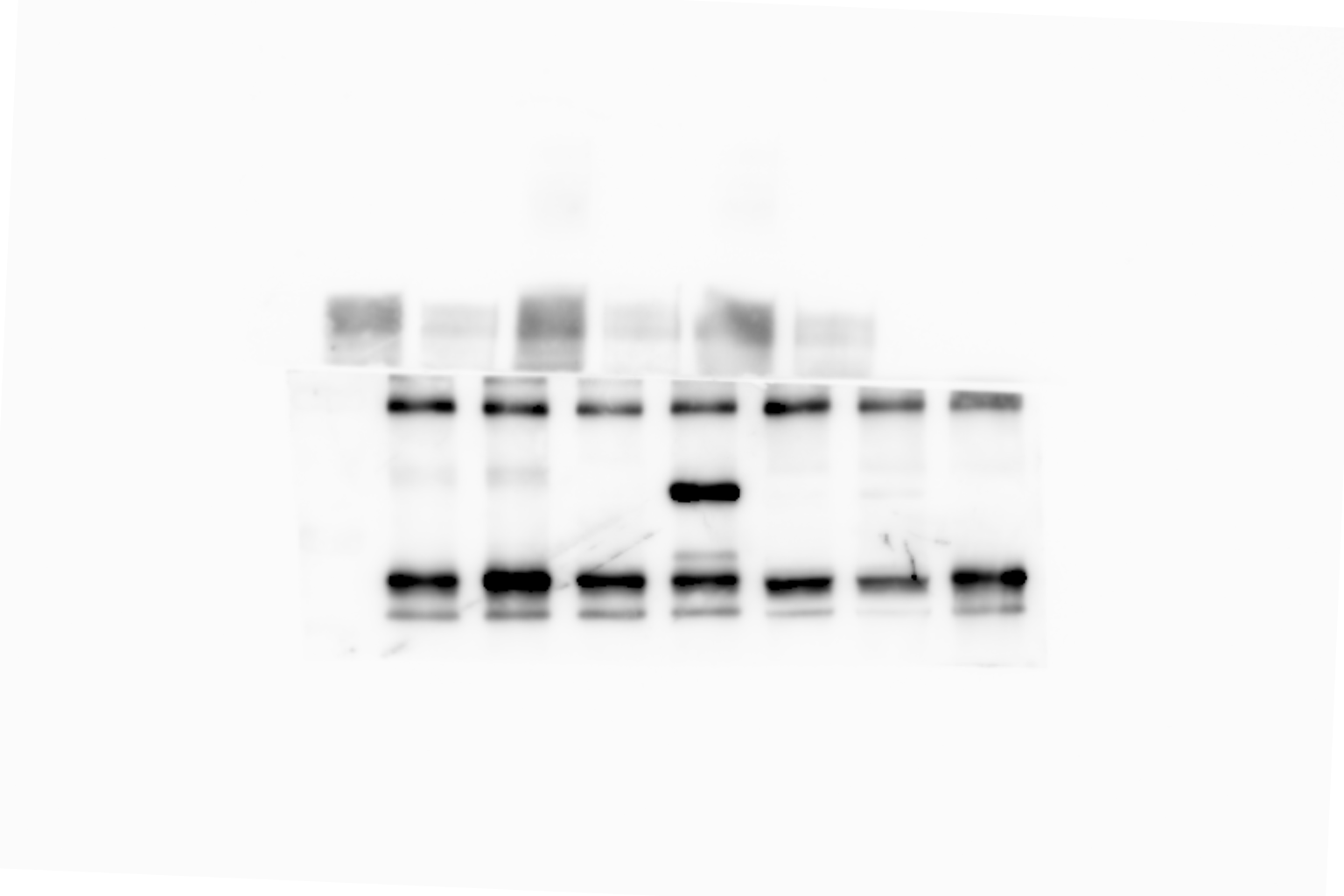

Supplement: Supplementary file 3 — Source data Fig. 1 [file 44321_2025_216_MOESM3_ESM.zip › Figure 1/Figure 1L/Figure 1L left panel.tif]

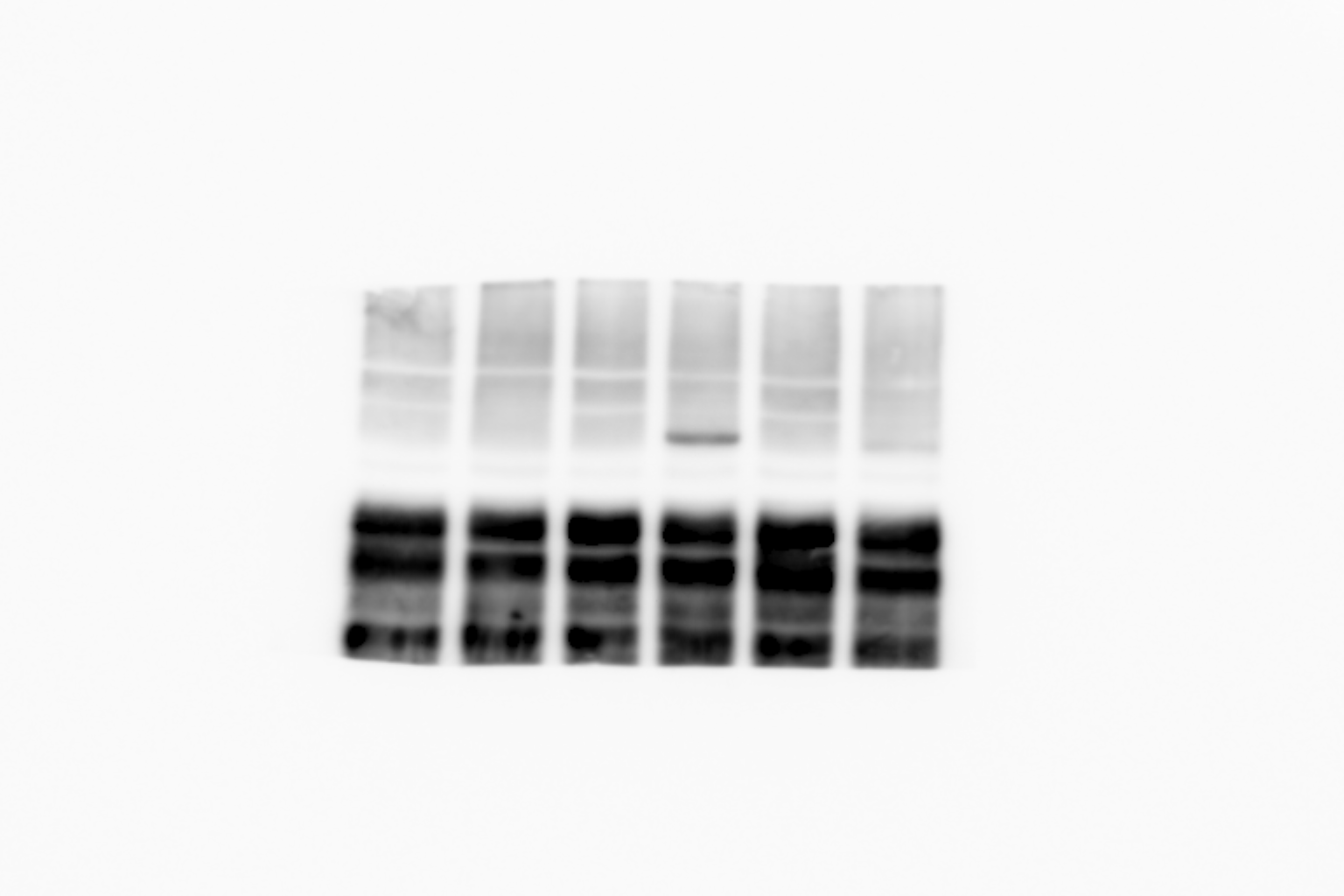

Supplement: Supplementary file 3 — Source data Fig. 1 [file 44321_2025_216_MOESM3_ESM.zip › Figure 1/Figure 1L/Figure 1L right panel.tif]

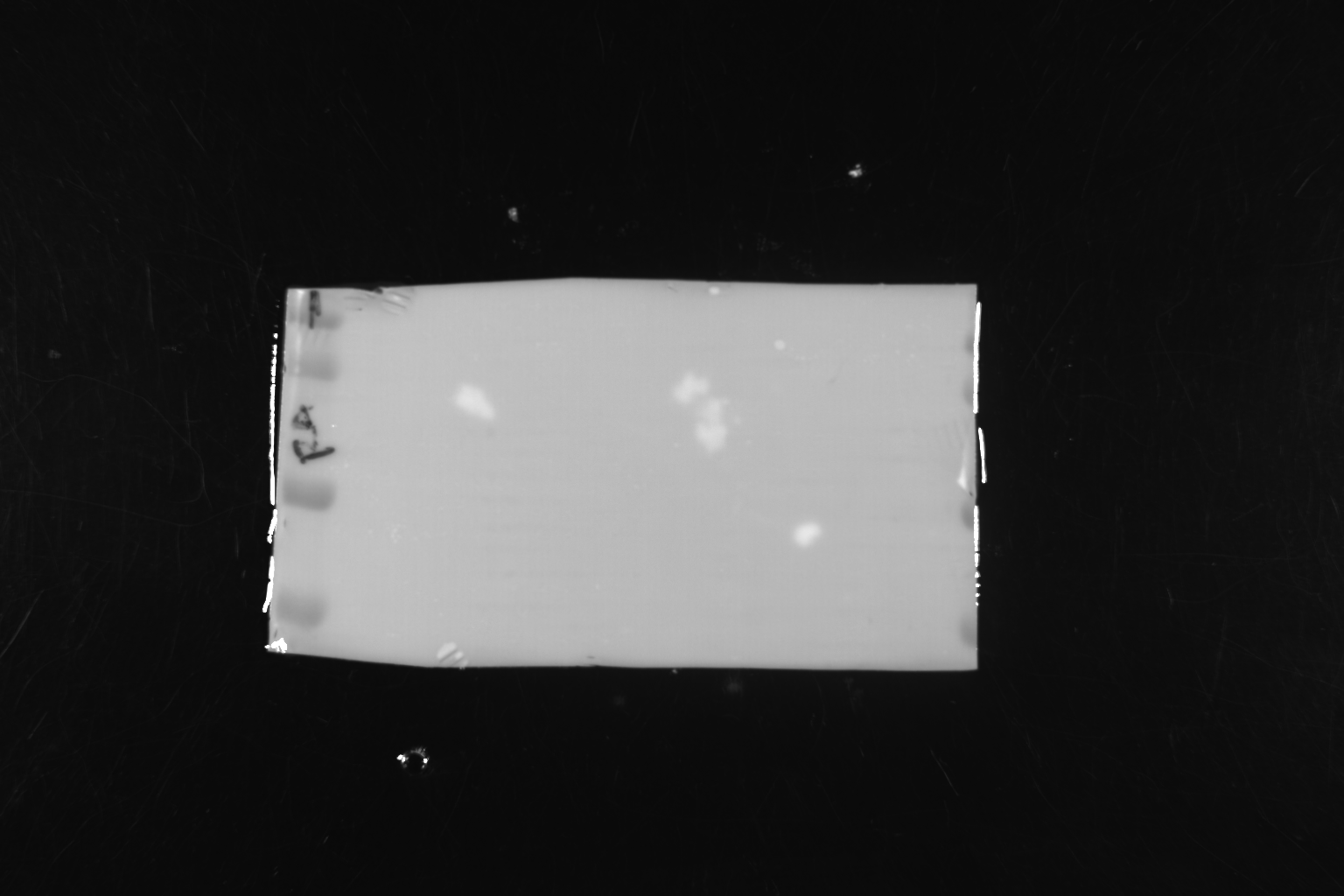

Supplement: Supplementary file 3 — Source data Fig. 1 [file 44321_2025_216_MOESM3_ESM.zip › Figure 1/Figure 1L/Figure 1L right panel blanks and markers.tif]

## Slide 1
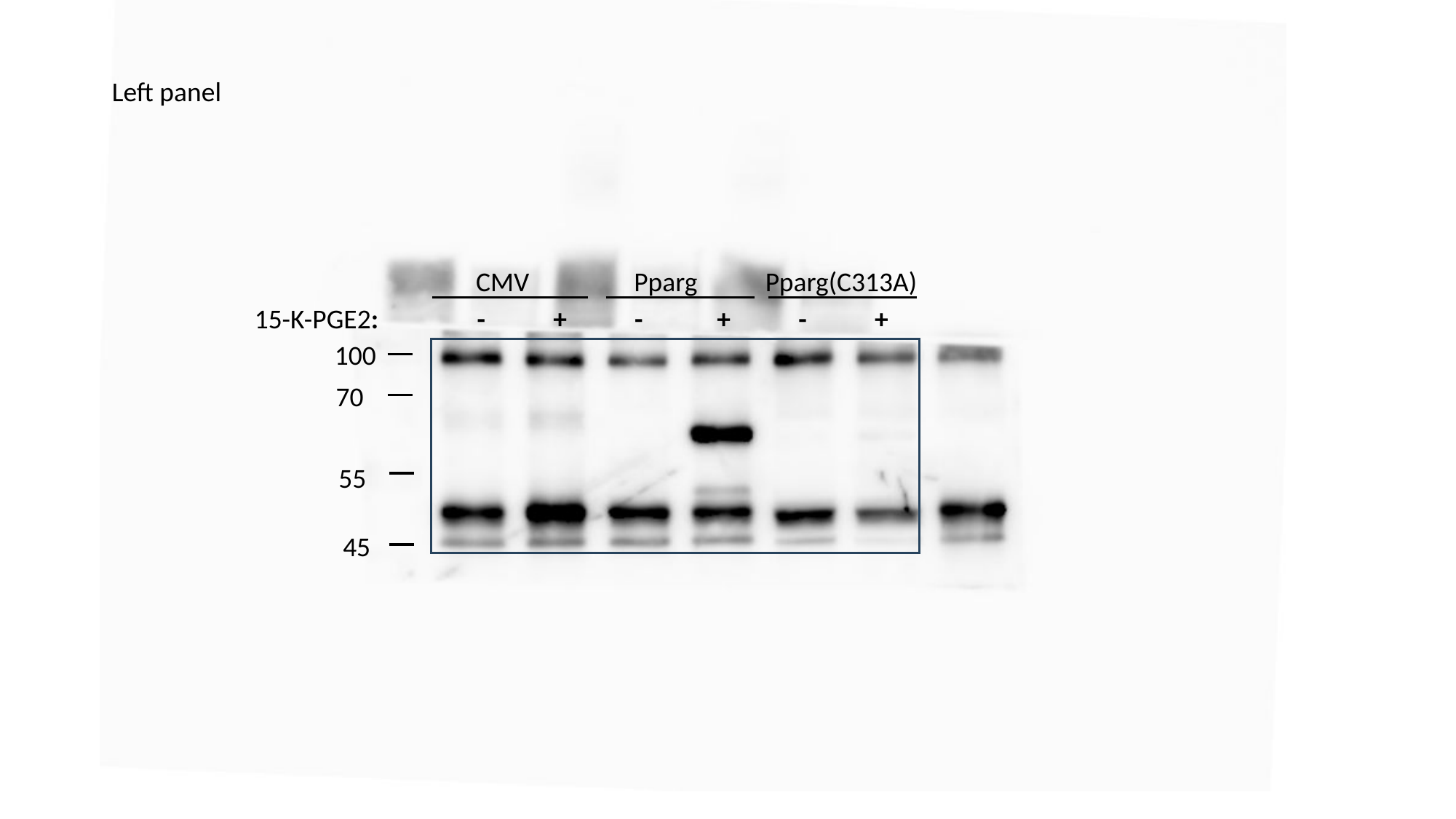

Left panel
#
CMV Pparg Pparg(C313A)
15-K-PGE2: - + - + - +
100
70
55
45

## Slide 2
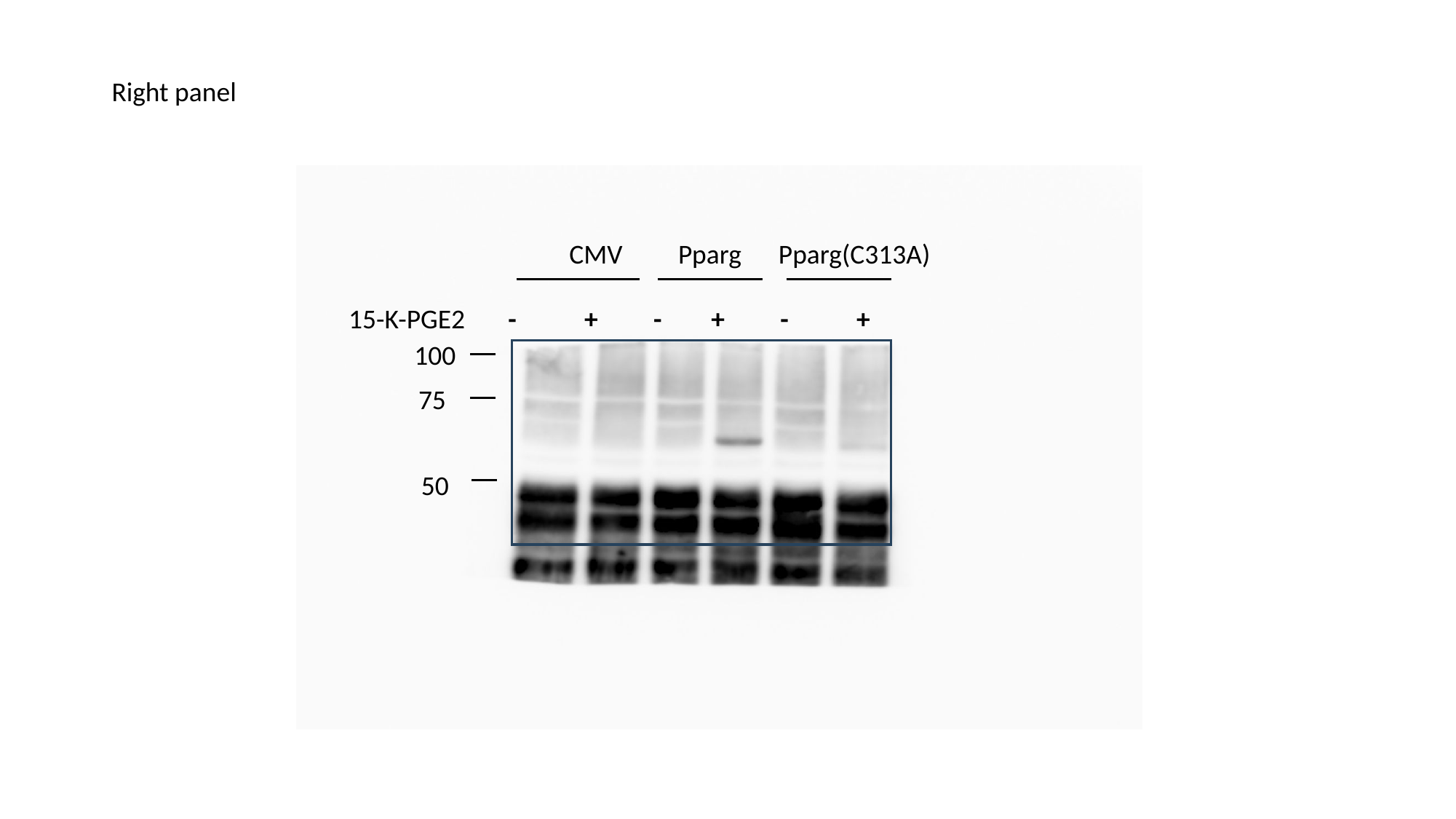

Right panel
CMV Pparg Pparg(C313A)
 15-K-PGE2 - + - + - +
100
75
50

Supplement: Supplementary file 3 — Source data Fig. 1 [file 44321_2025_216_MOESM3_ESM.zip › Figure 1/Figure 1L/Figure 1L.pptx]

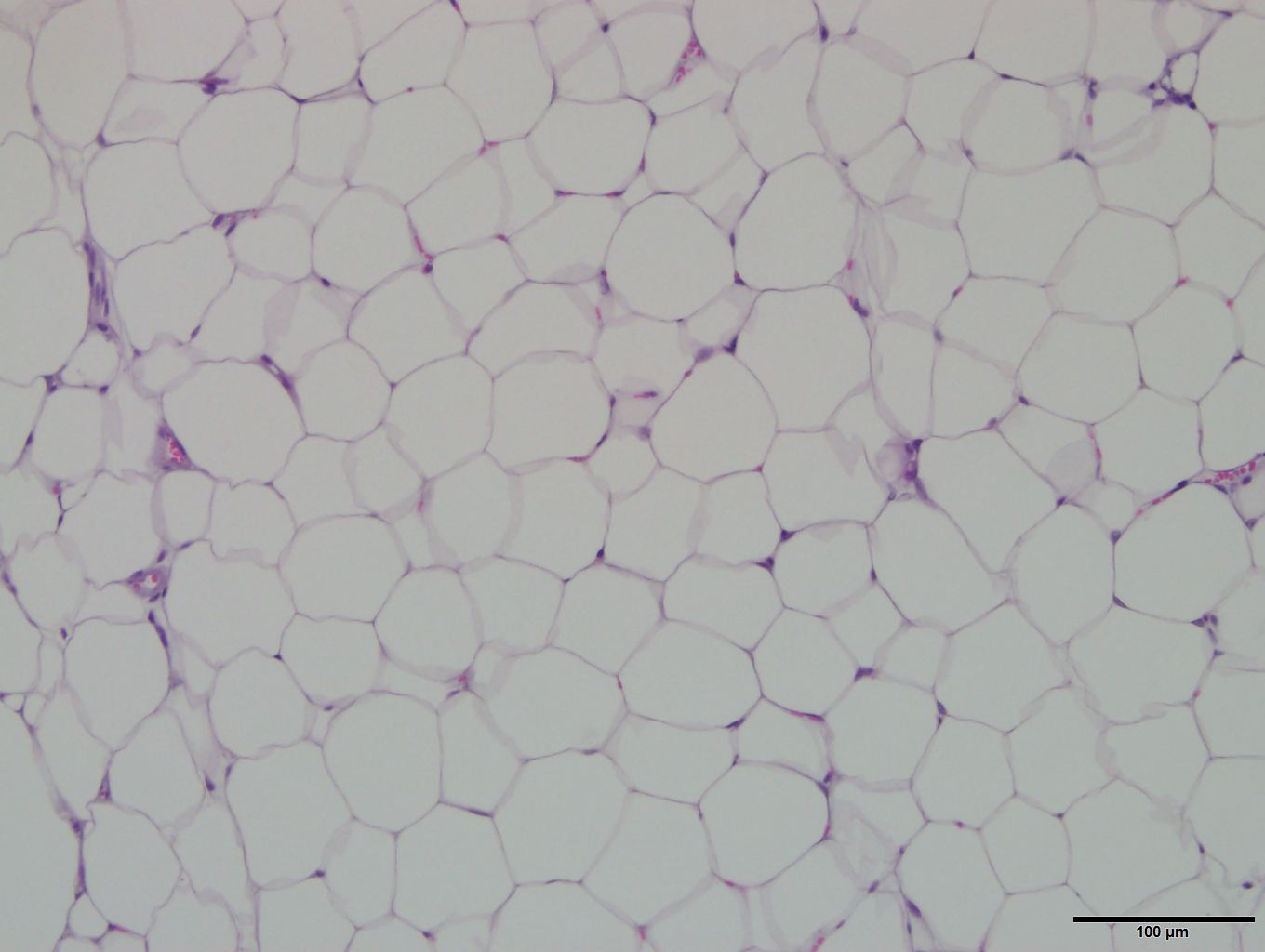

Supplement: Supplementary file 4 — Source data Fig. 2 [file 44321_2025_216_MOESM4_ESM.zip › Figure 2/Figure 2M lower panel.jpg]

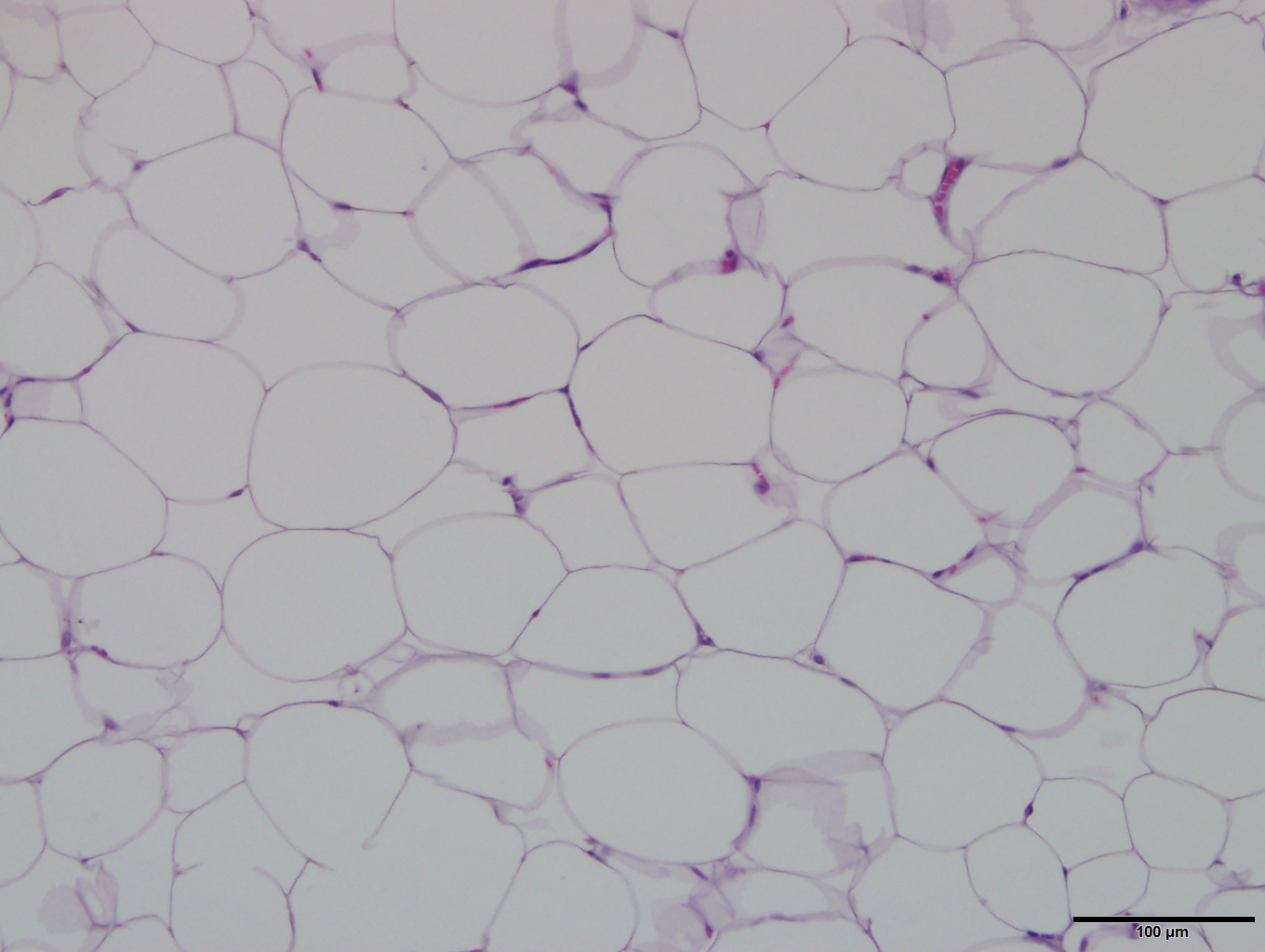

Supplement: Supplementary file 4 — Source data Fig. 2 [file 44321_2025_216_MOESM4_ESM.zip › Figure 2/Figure 2M upper panel.jpg]

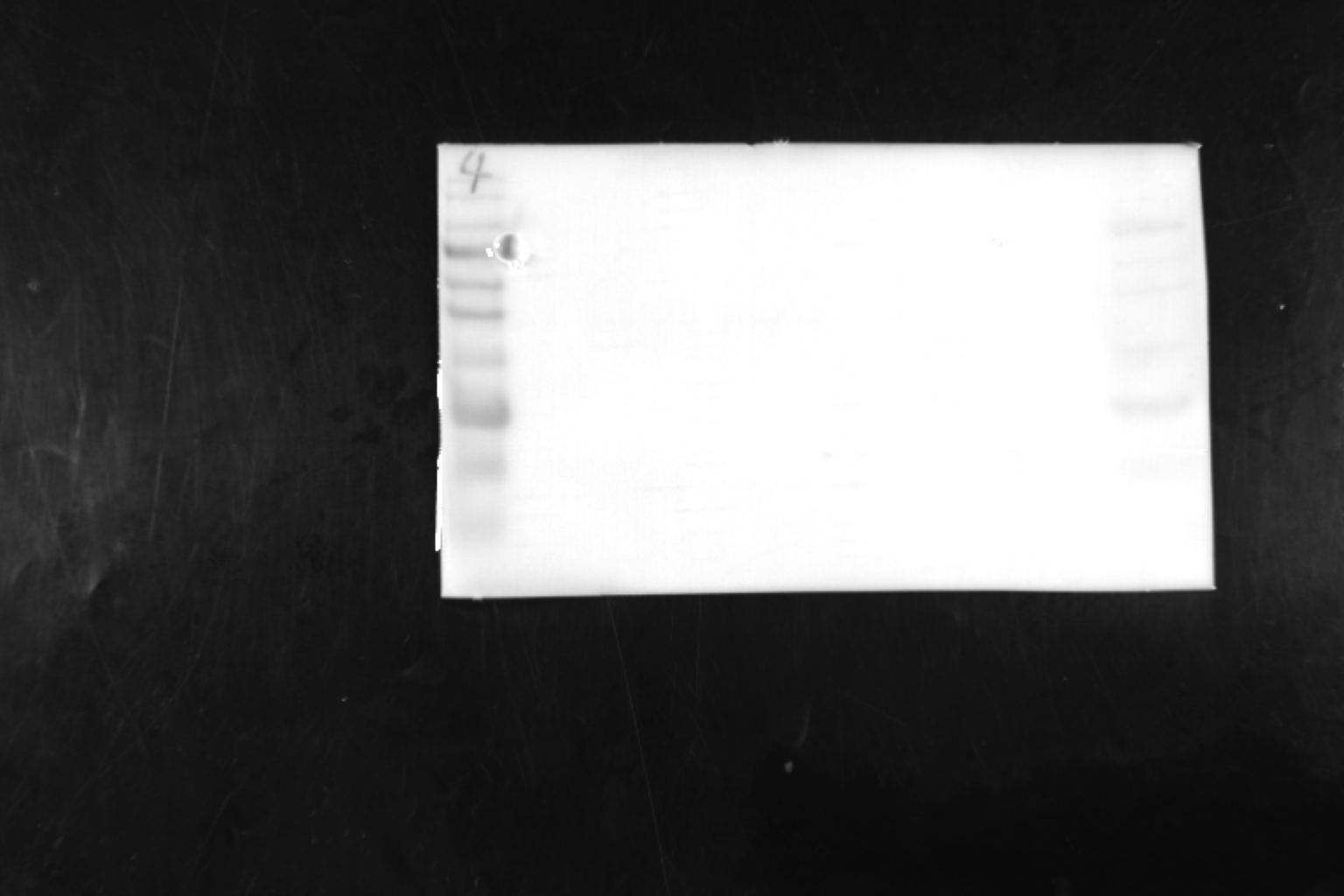

Supplement: Supplementary file 4 — Source data Fig. 2 [file 44321_2025_216_MOESM4_ESM.zip › Figure 2/Figure 2L/Skeletal muscle/akt/20170719_1000m.tif]

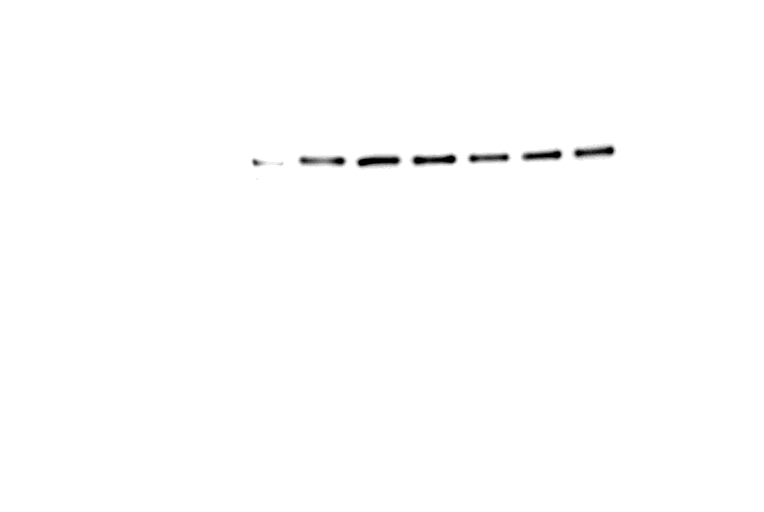

Supplement: Supplementary file 4 — Source data Fig. 2 [file 44321_2025_216_MOESM4_ESM.zip › Figure 2/Figure 2L/Skeletal muscle/akt/akt1.tif]

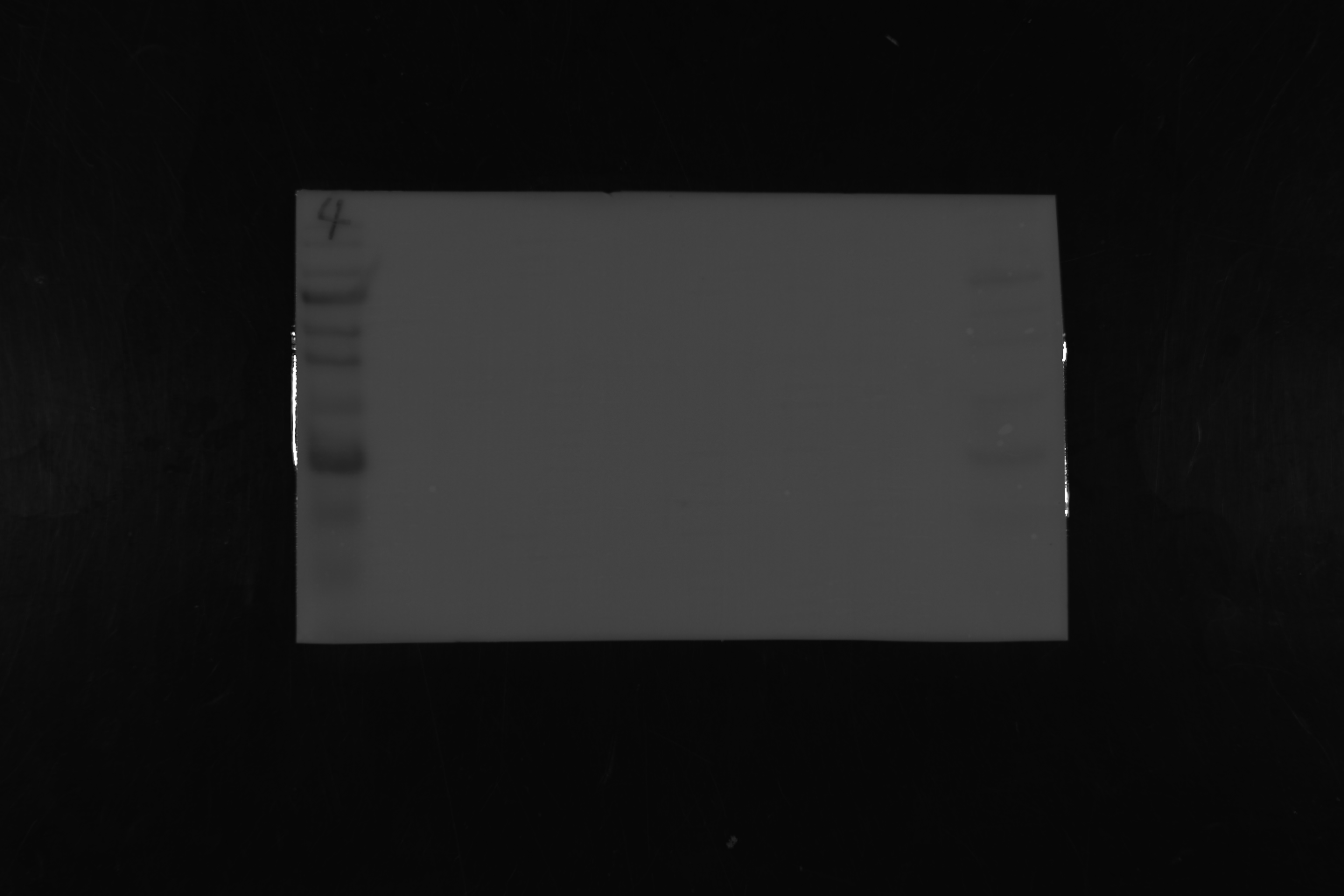

Supplement: Supplementary file 4 — Source data Fig. 2 [file 44321_2025_216_MOESM4_ESM.zip › Figure 2/Figure 2L/Skeletal muscle/p-akt/20170715_1252m.tif]

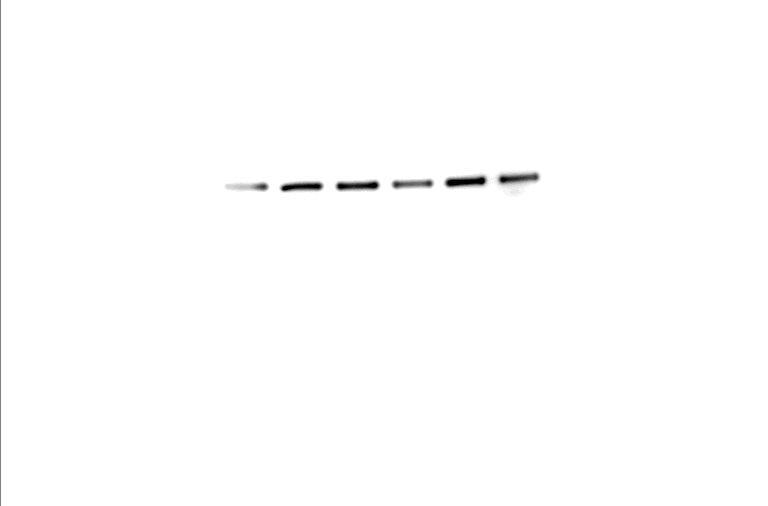

Supplement: Supplementary file 4 — Source data Fig. 2 [file 44321_2025_216_MOESM4_ESM.zip › Figure 2/Figure 2L/Skeletal muscle/p-akt/Untitled3.tif]

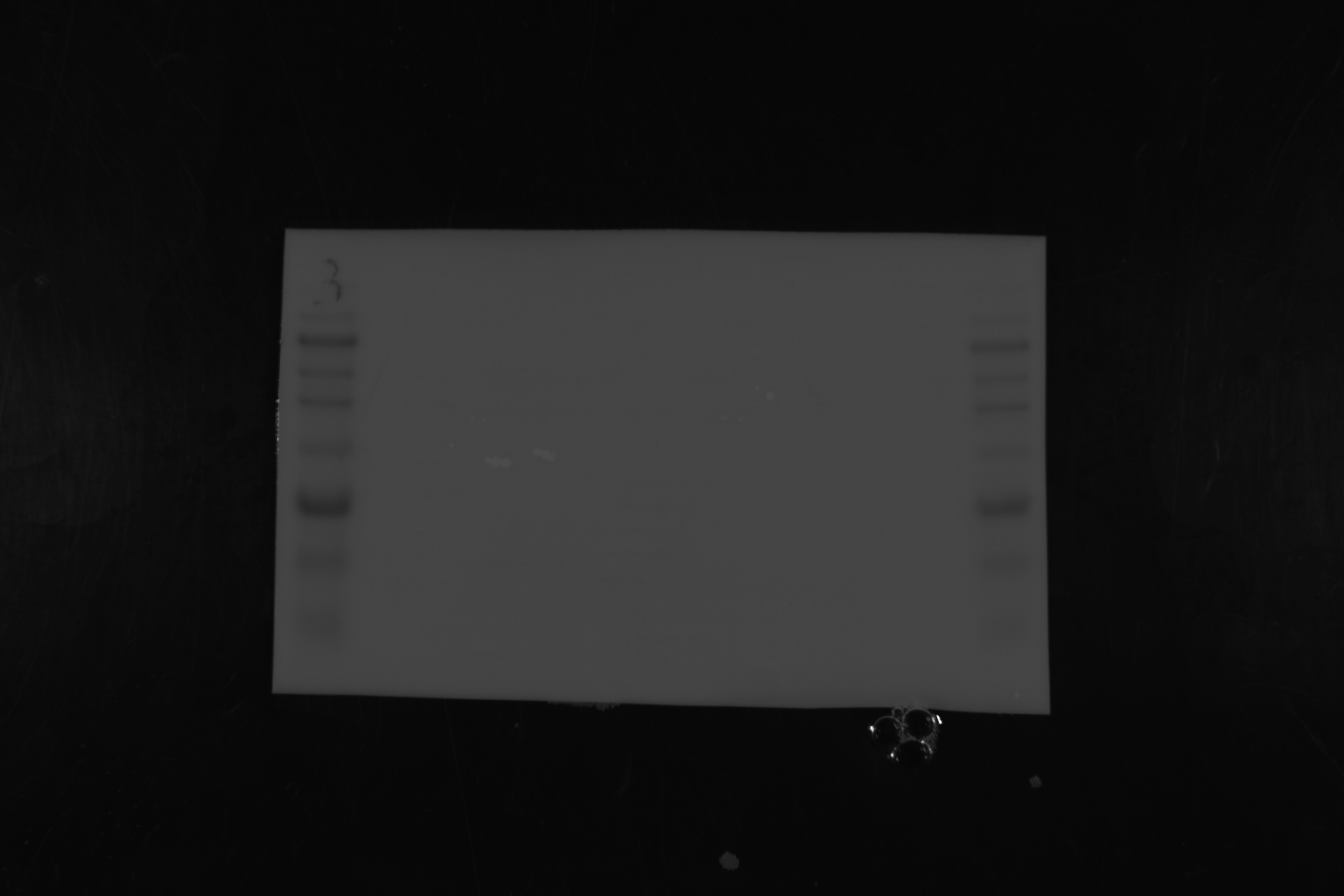

Supplement: Supplementary file 4 — Source data Fig. 2 [file 44321_2025_216_MOESM4_ESM.zip › Figure 2/Figure 2L/Brown adipose tissue/PAkt/20170715_1255m.tif]

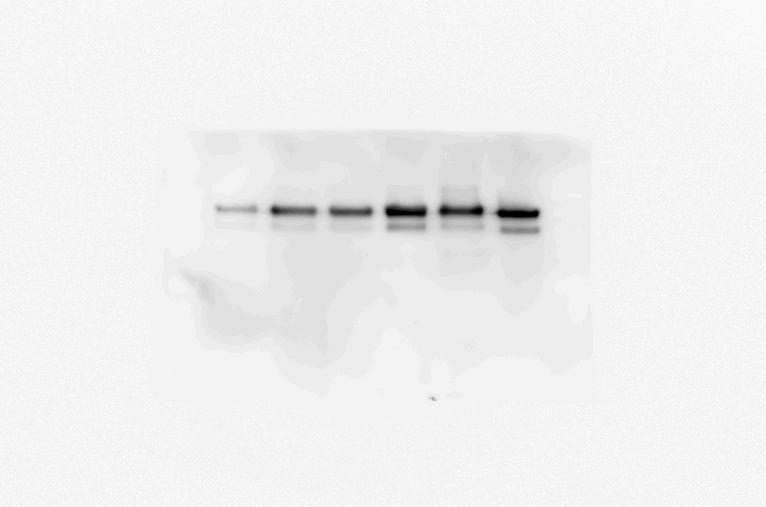

Supplement: Supplementary file 4 — Source data Fig. 2 [file 44321_2025_216_MOESM4_ESM.zip › Figure 2/Figure 2L/Brown adipose tissue/PAkt/Untitled1.tif]

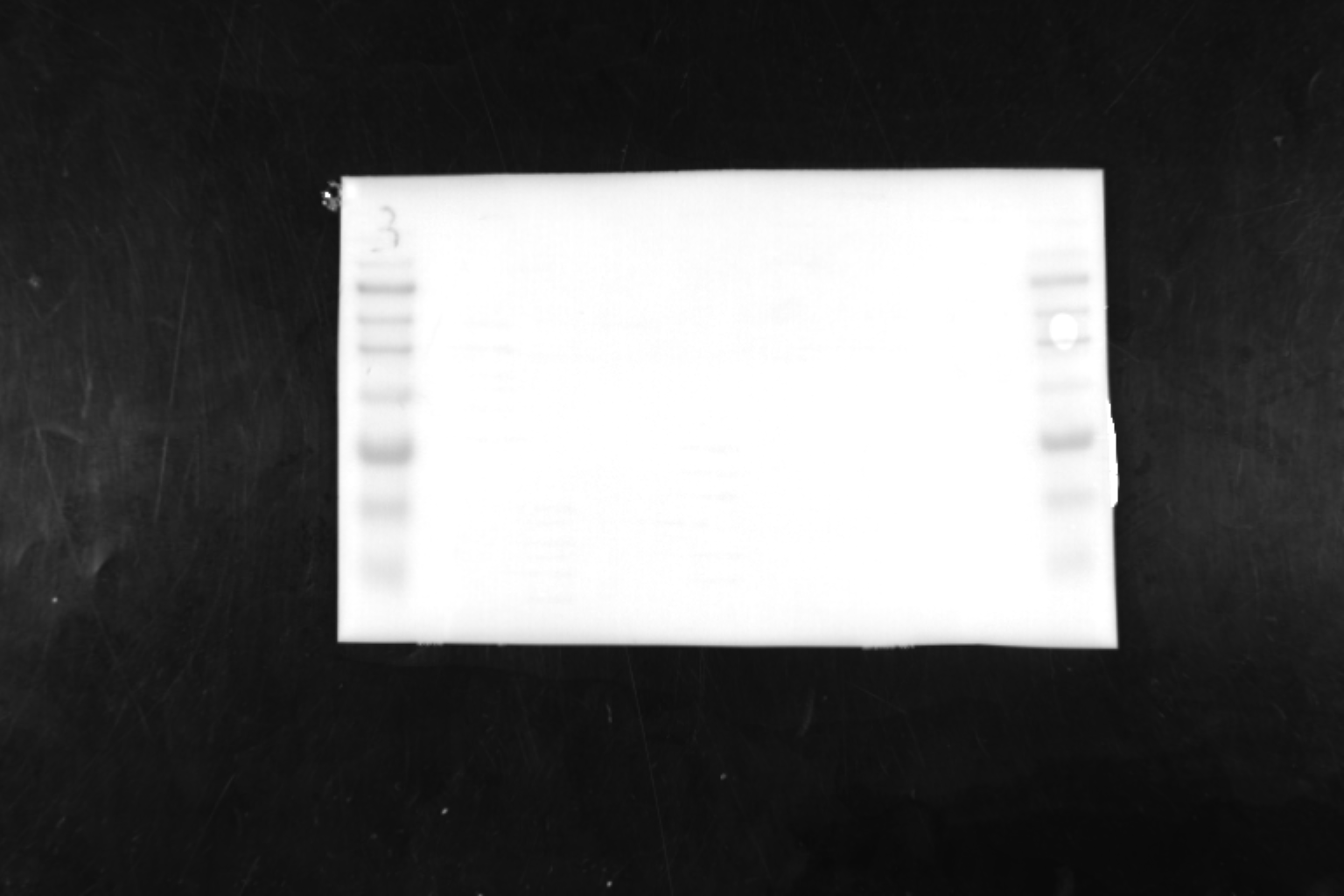

Supplement: Supplementary file 4 — Source data Fig. 2 [file 44321_2025_216_MOESM4_ESM.zip › Figure 2/Figure 2L/Brown adipose tissue/Akt/20170719_0946m.tif]

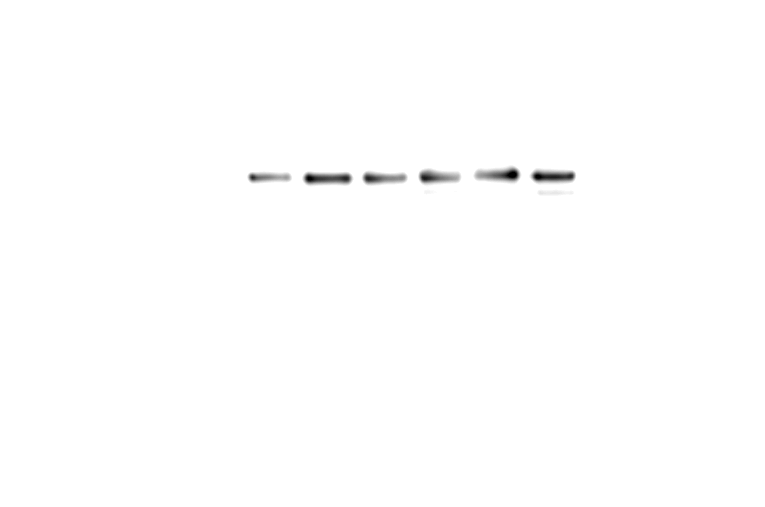

Supplement: Supplementary file 4 — Source data Fig. 2 [file 44321_2025_216_MOESM4_ESM.zip › Figure 2/Figure 2L/Brown adipose tissue/Akt/akt4.tif]

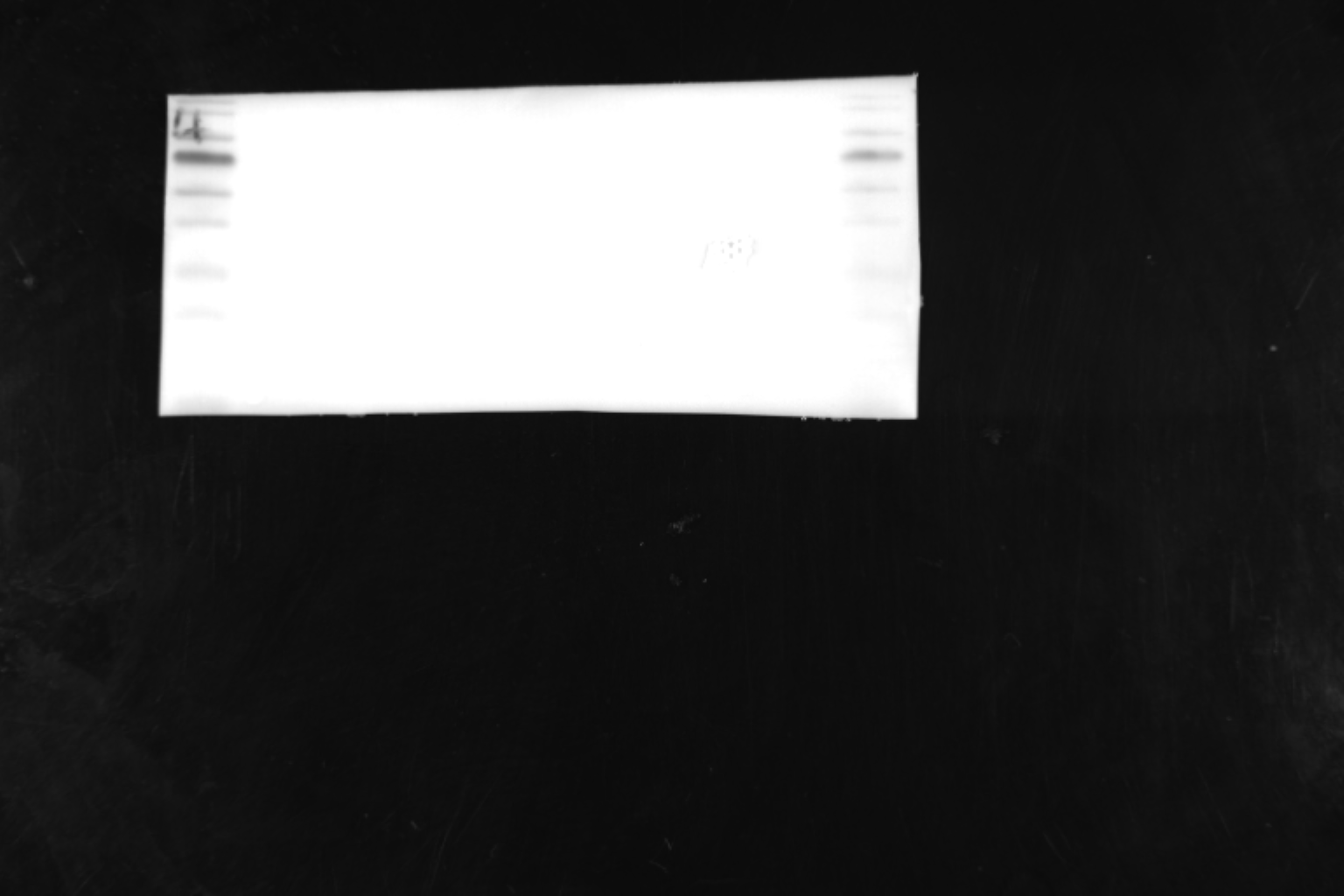

Supplement: Supplementary file 4 — Source data Fig. 2 [file 44321_2025_216_MOESM4_ESM.zip › Figure 2/Figure 2L/Inguinal fat/pAkt/20170805_1742m.tif]

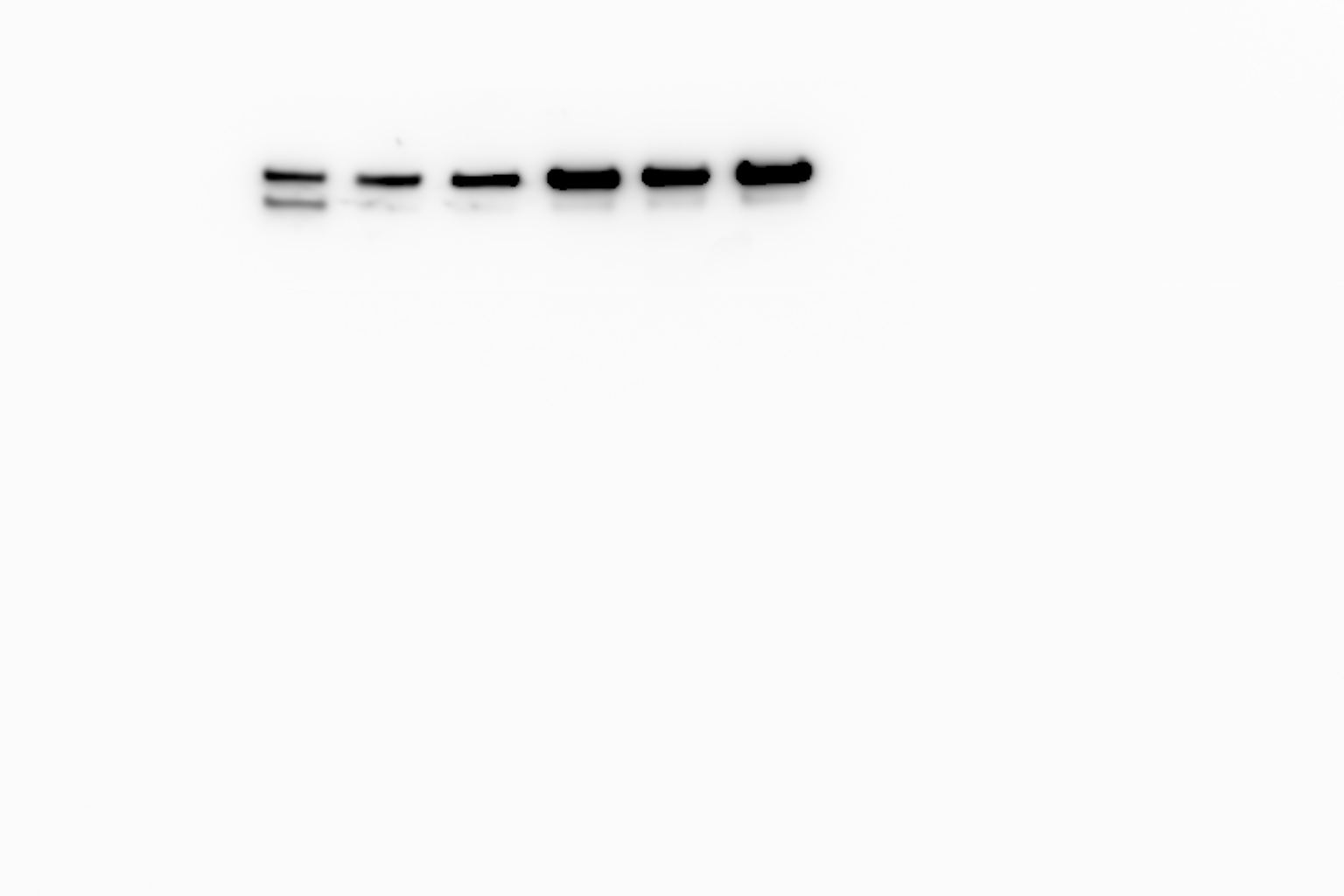

Supplement: Supplementary file 4 — Source data Fig. 2 [file 44321_2025_216_MOESM4_ESM.zip › Figure 2/Figure 2L/Inguinal fat/pAkt/20170805_1742_7.tif]

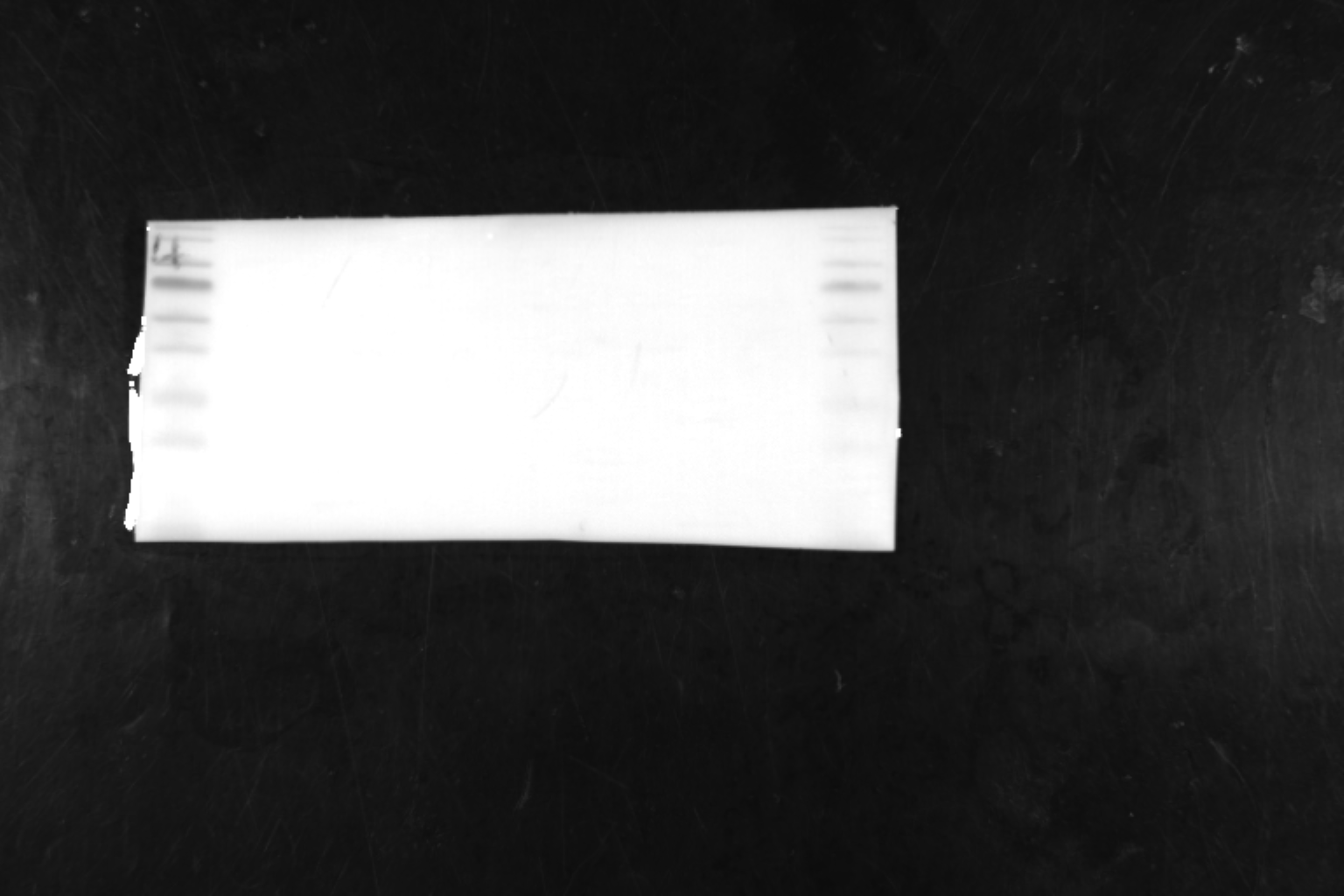

Supplement: Supplementary file 4 — Source data Fig. 2 [file 44321_2025_216_MOESM4_ESM.zip › Figure 2/Figure 2L/Inguinal fat/akt/20170810_1940m.tif]

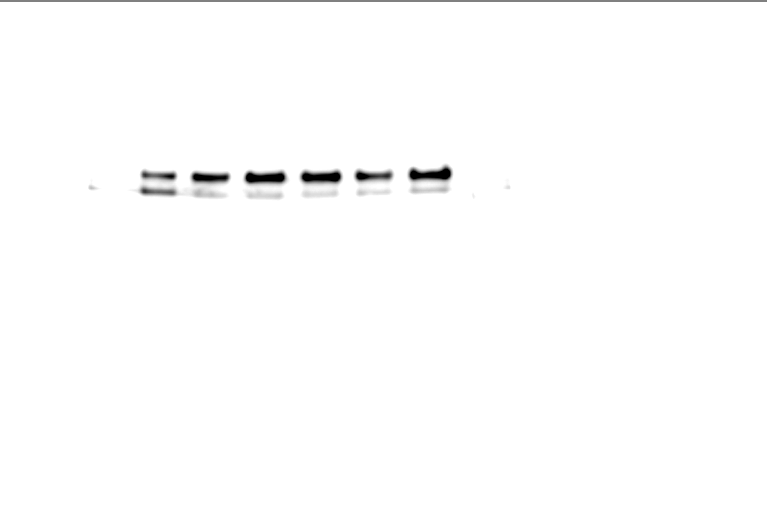

Supplement: Supplementary file 4 — Source data Fig. 2 [file 44321_2025_216_MOESM4_ESM.zip › Figure 2/Figure 2L/Inguinal fat/akt/akt2.tif]

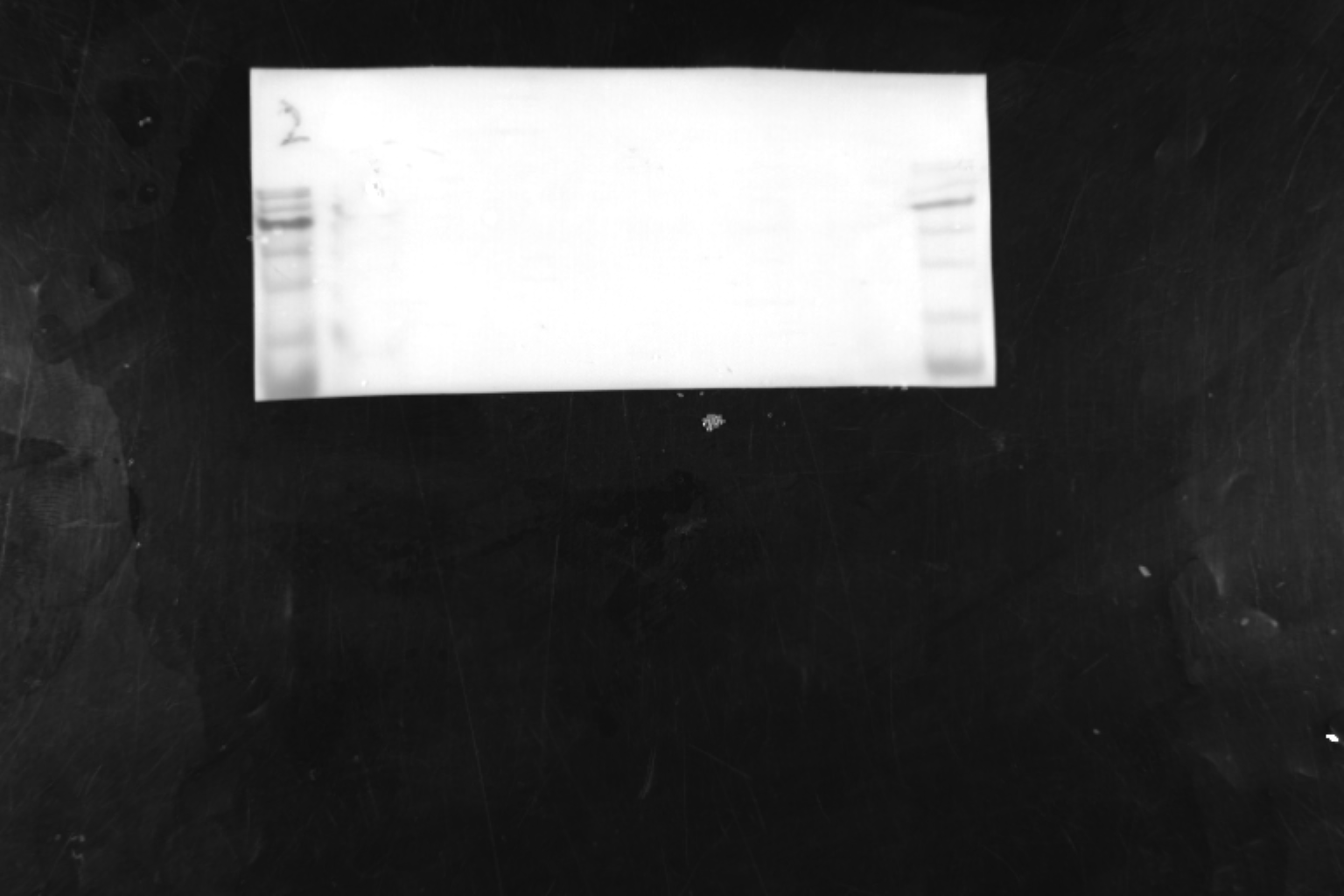

Supplement: Supplementary file 4 — Source data Fig. 2 [file 44321_2025_216_MOESM4_ESM.zip › Figure 2/Figure 2L/Perigonadal fat/akt/Marker.tif]

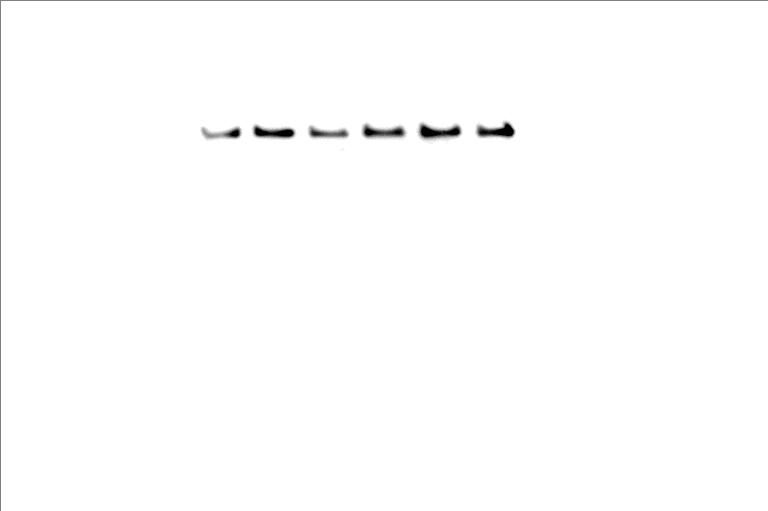

Supplement: Supplementary file 4 — Source data Fig. 2 [file 44321_2025_216_MOESM4_ESM.zip › Figure 2/Figure 2L/Perigonadal fat/akt/Akt.tif]

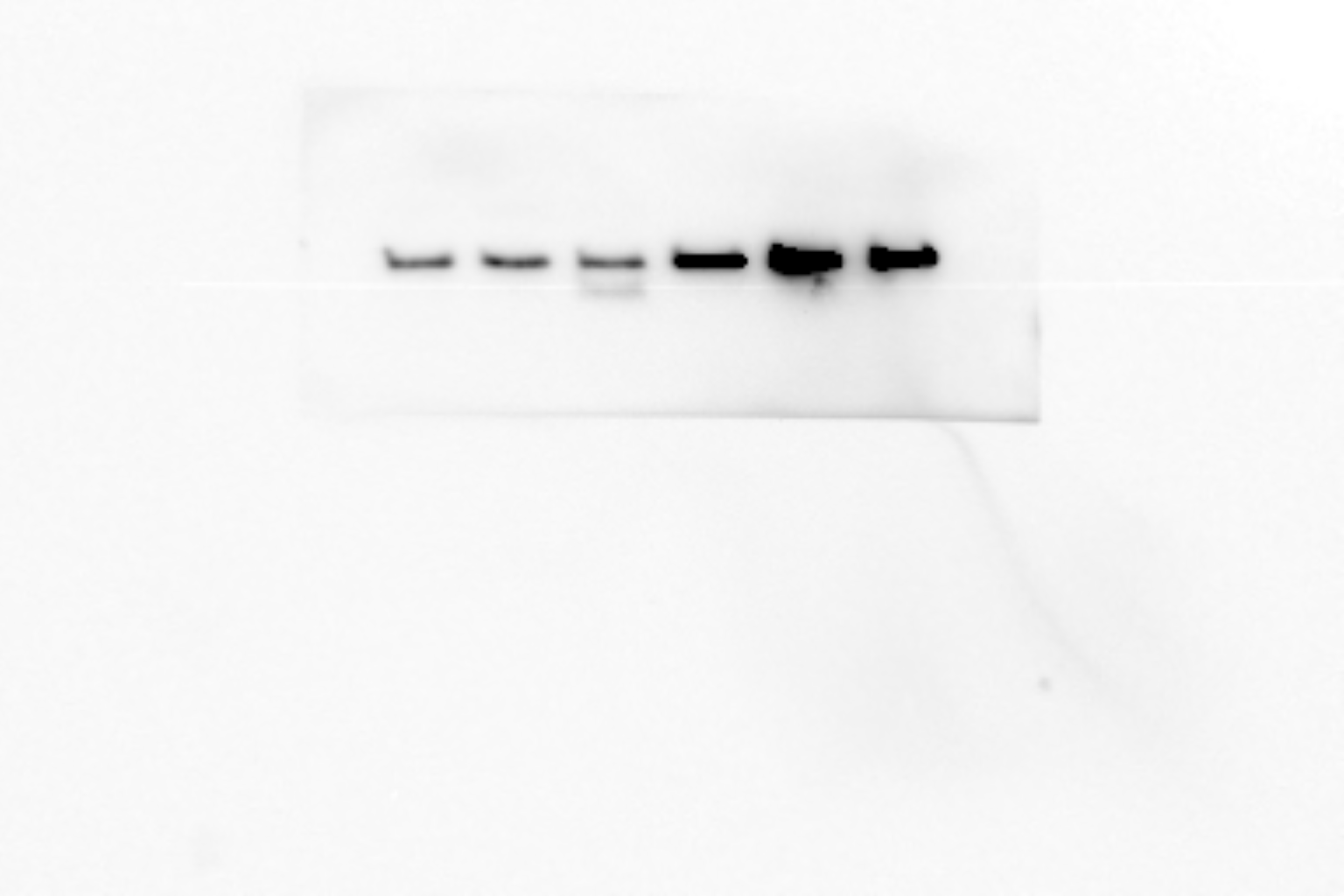

Supplement: Supplementary file 4 — Source data Fig. 2 [file 44321_2025_216_MOESM4_ESM.zip › Figure 2/Figure 2L/Perigonadal fat/p-akt/pAkt.tif]

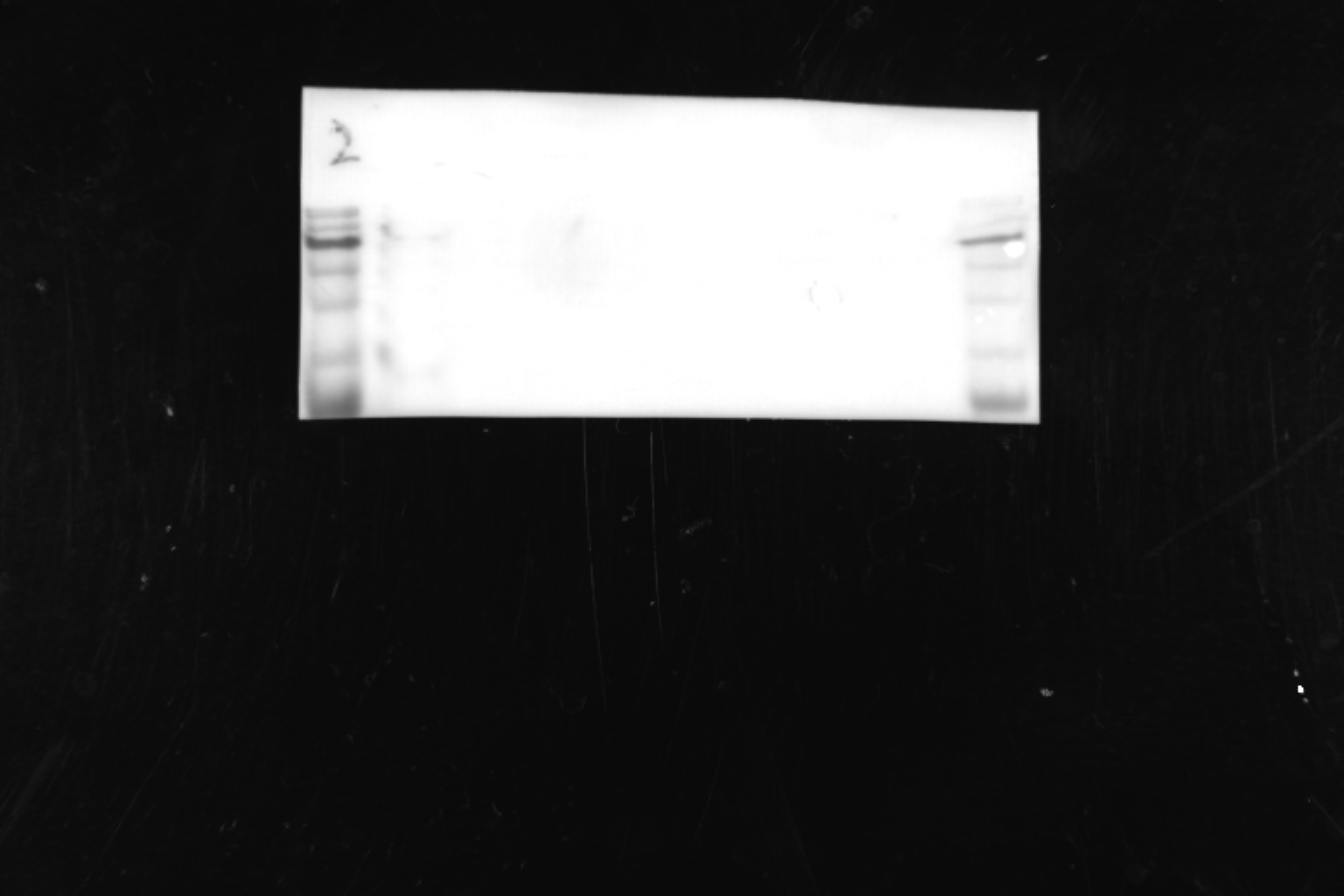

Supplement: Supplementary file 4 — Source data Fig. 2 [file 44321_2025_216_MOESM4_ESM.zip › Figure 2/Figure 2L/Perigonadal fat/p-akt/Marker.tif]

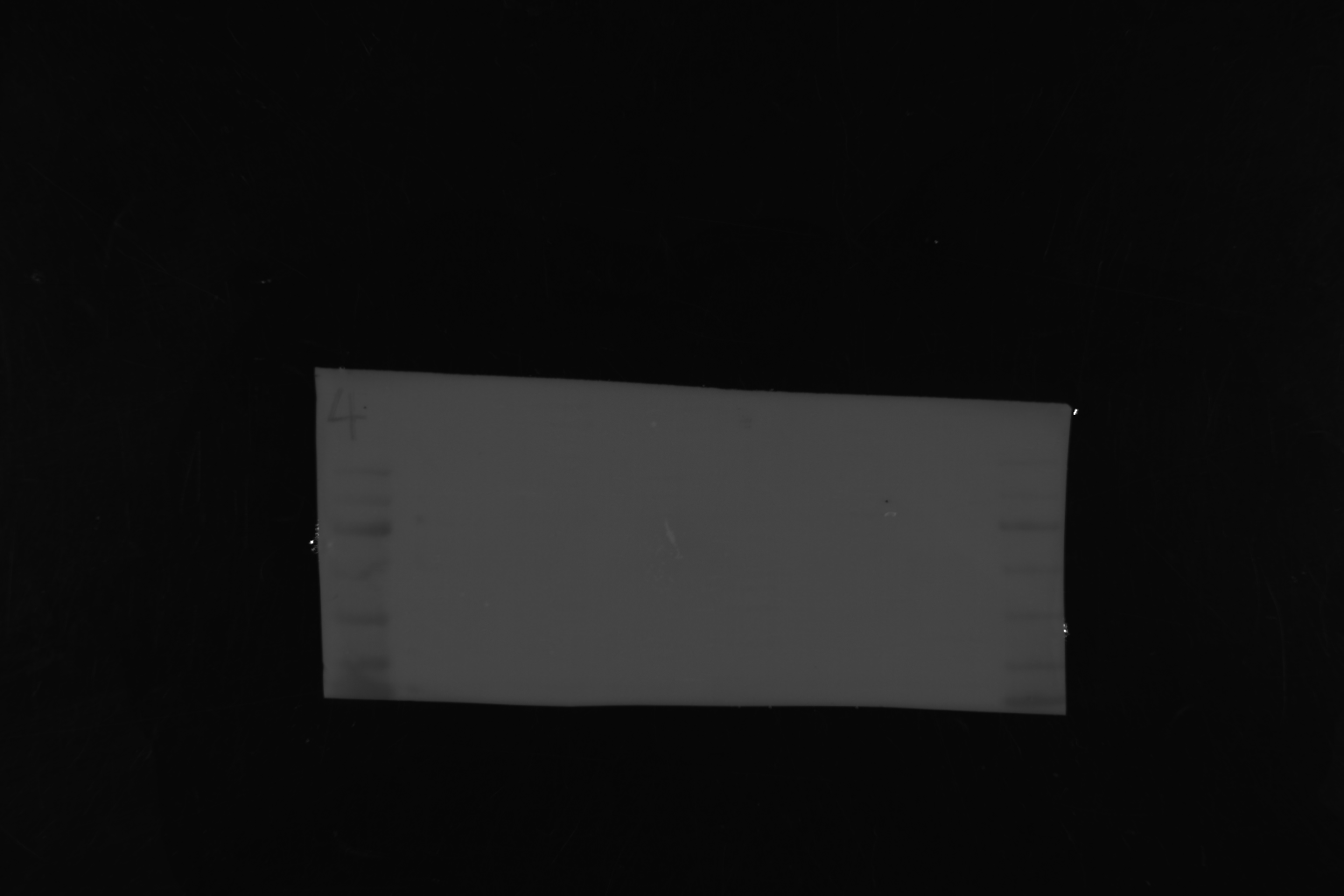

Supplement: Supplementary file 4 — Source data Fig. 2 [file 44321_2025_216_MOESM4_ESM.zip › Figure 2/Figure 2L/Liver/akt/20170708_1109m.tif]

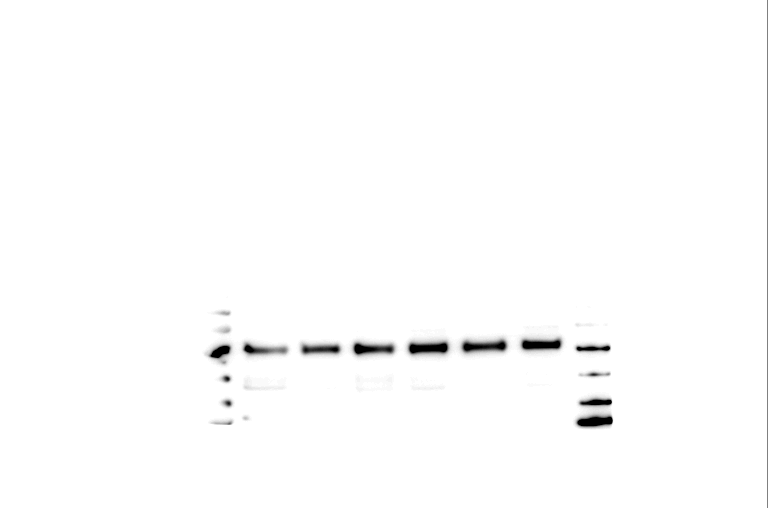

Supplement: Supplementary file 4 — Source data Fig. 2 [file 44321_2025_216_MOESM4_ESM.zip › Figure 2/Figure 2L/Liver/akt/Untitled3.tif]

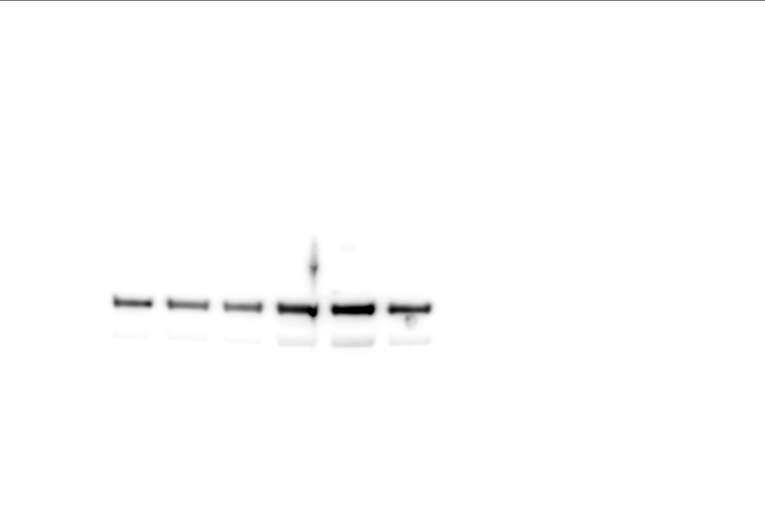

Supplement: Supplementary file 4 — Source data Fig. 2 [file 44321_2025_216_MOESM4_ESM.zip › Figure 2/Figure 2L/Liver/p-akt/Untitled7.tif]

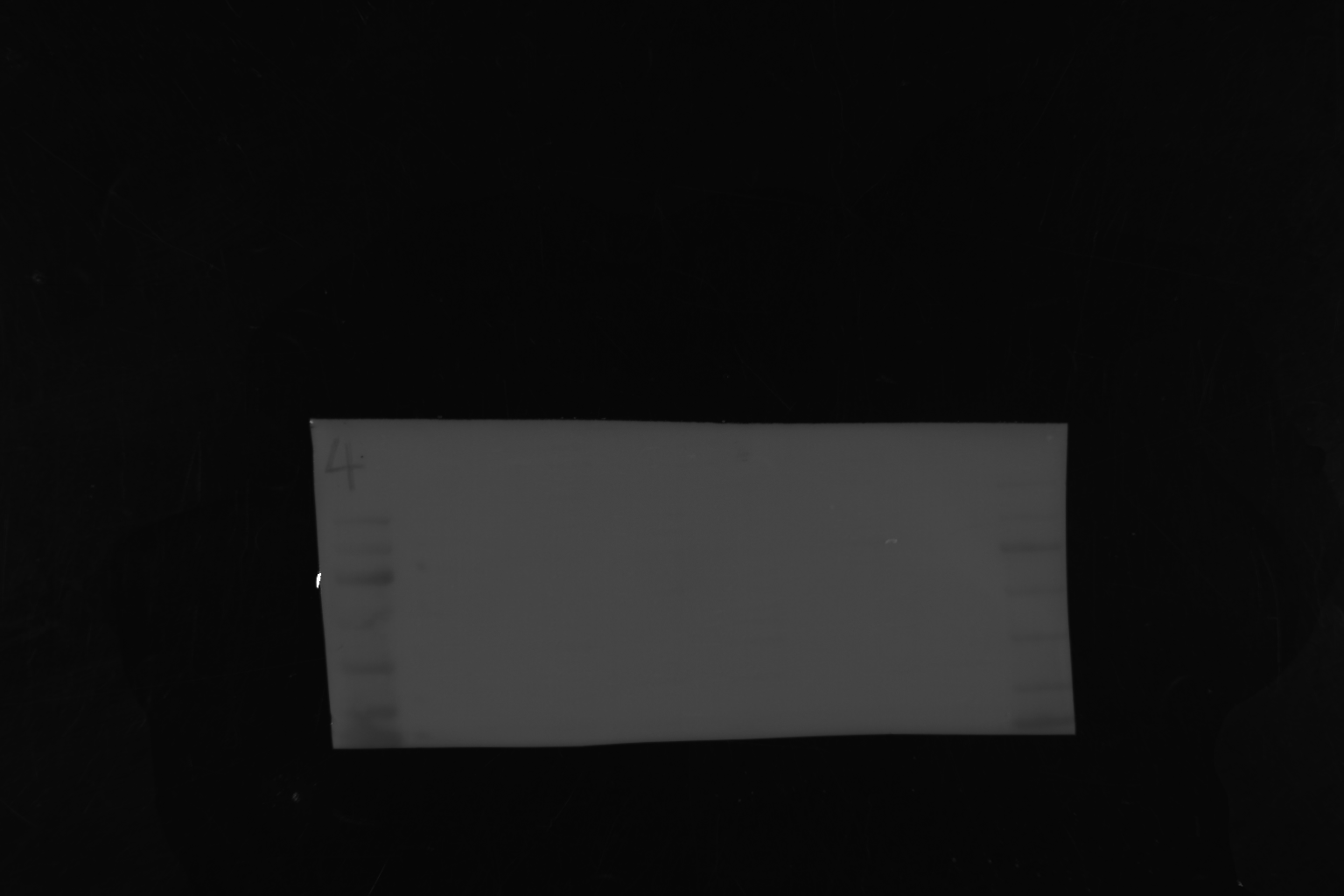

Supplement: Supplementary file 4 — Source data Fig. 2 [file 44321_2025_216_MOESM4_ESM.zip › Figure 2/Figure 2L/Liver/p-akt/20170704_1034m.tif]

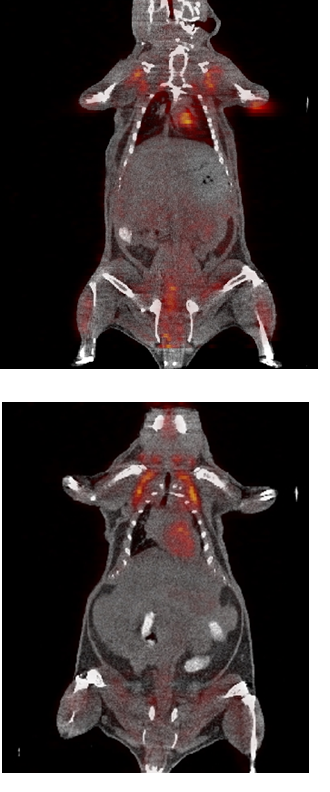

Supplement: Supplementary file 5 — Source data Fig. 3 [file 44321_2025_216_MOESM5_ESM.zip › Figure 3/Figure 3E.tif]

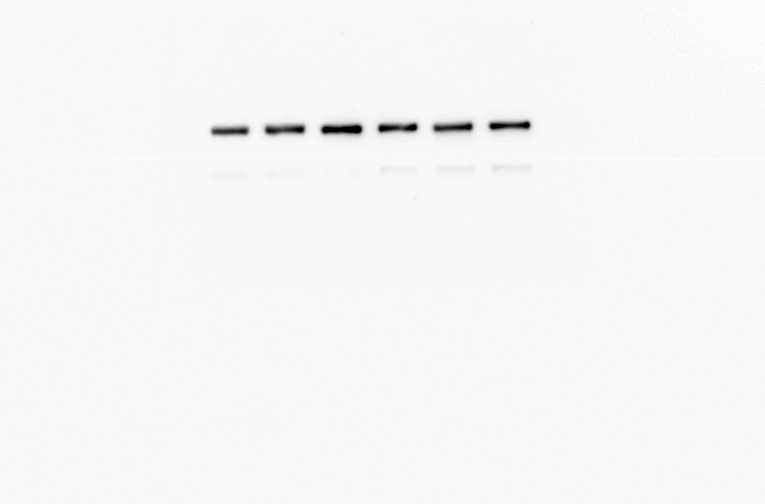

Supplement: Supplementary file 5 — Source data Fig. 3 [file 44321_2025_216_MOESM5_ESM.zip › Figure 3/Figure 3G/Brown adipose tissue/Akt/Akt.tif]

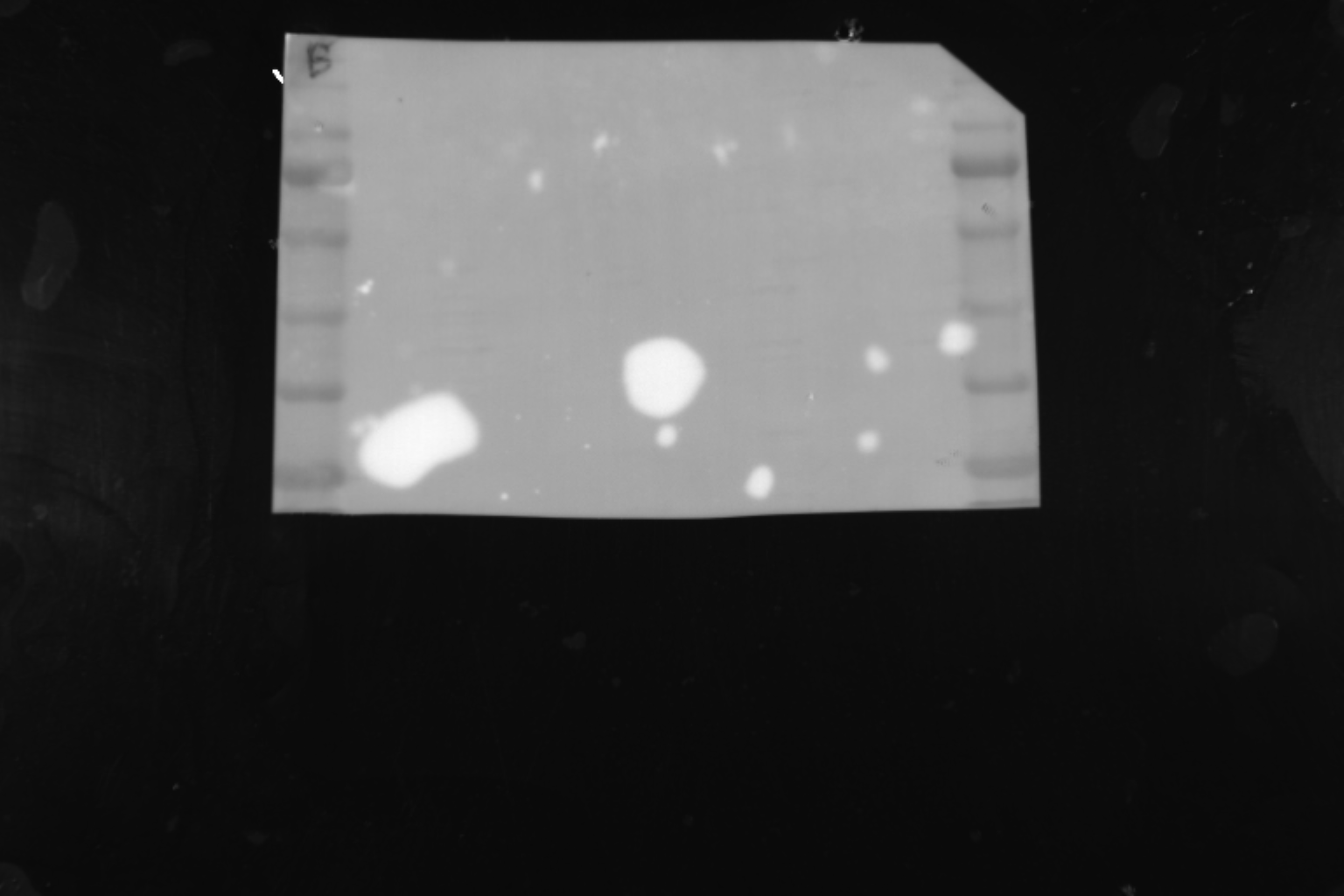

Supplement: Supplementary file 5 — Source data Fig. 3 [file 44321_2025_216_MOESM5_ESM.zip › Figure 3/Figure 3G/Brown adipose tissue/Akt/Marker.tif]

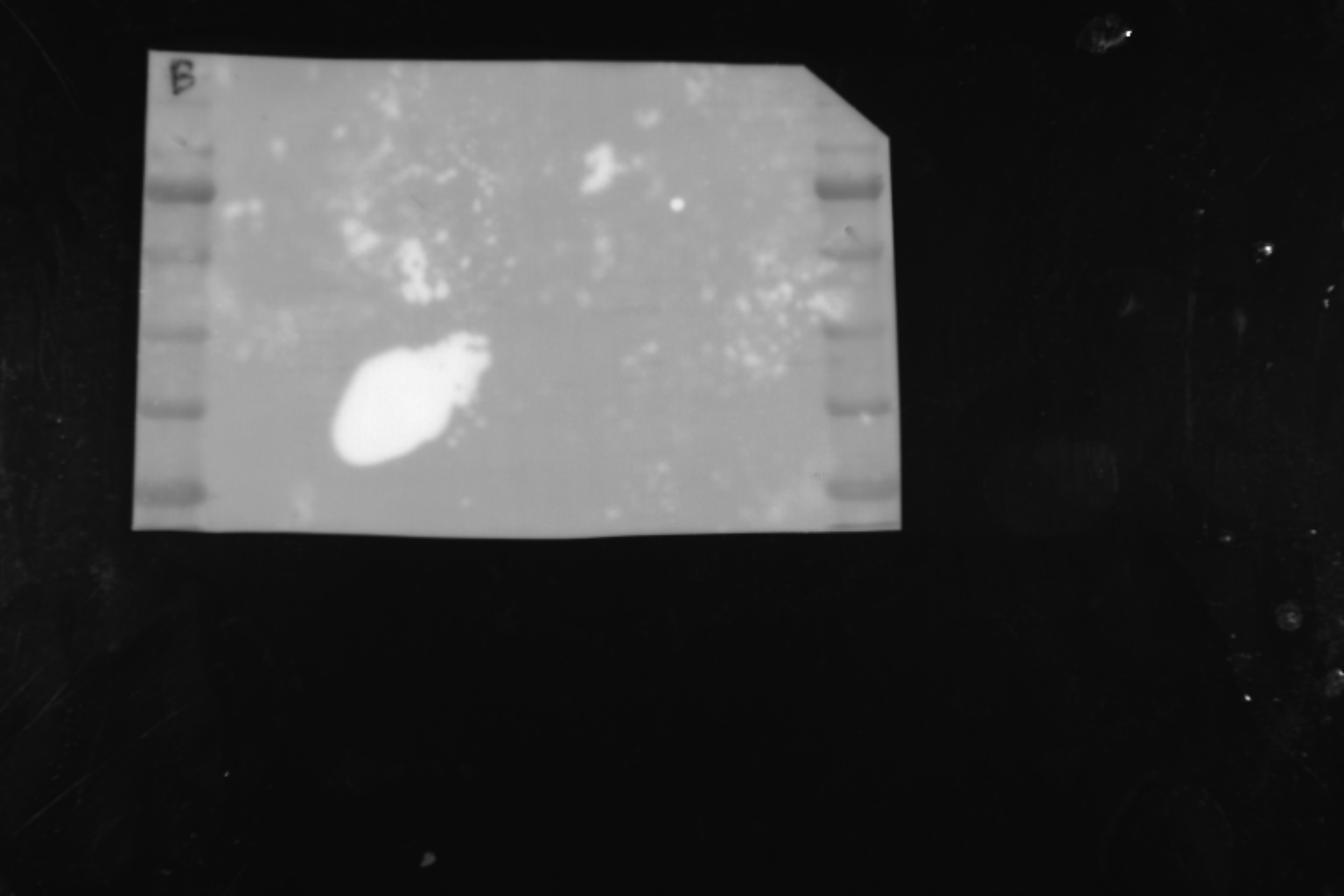

Supplement: Supplementary file 5 — Source data Fig. 3 [file 44321_2025_216_MOESM5_ESM.zip › Figure 3/Figure 3G/Brown adipose tissue/PAkt/Marker.tif]

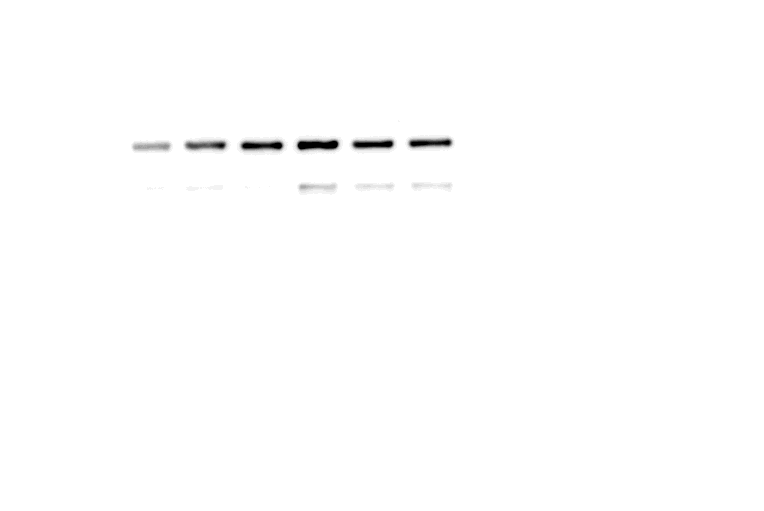

Supplement: Supplementary file 5 — Source data Fig. 3 [file 44321_2025_216_MOESM5_ESM.zip › Figure 3/Figure 3G/Brown adipose tissue/PAkt/pAkt.tif]

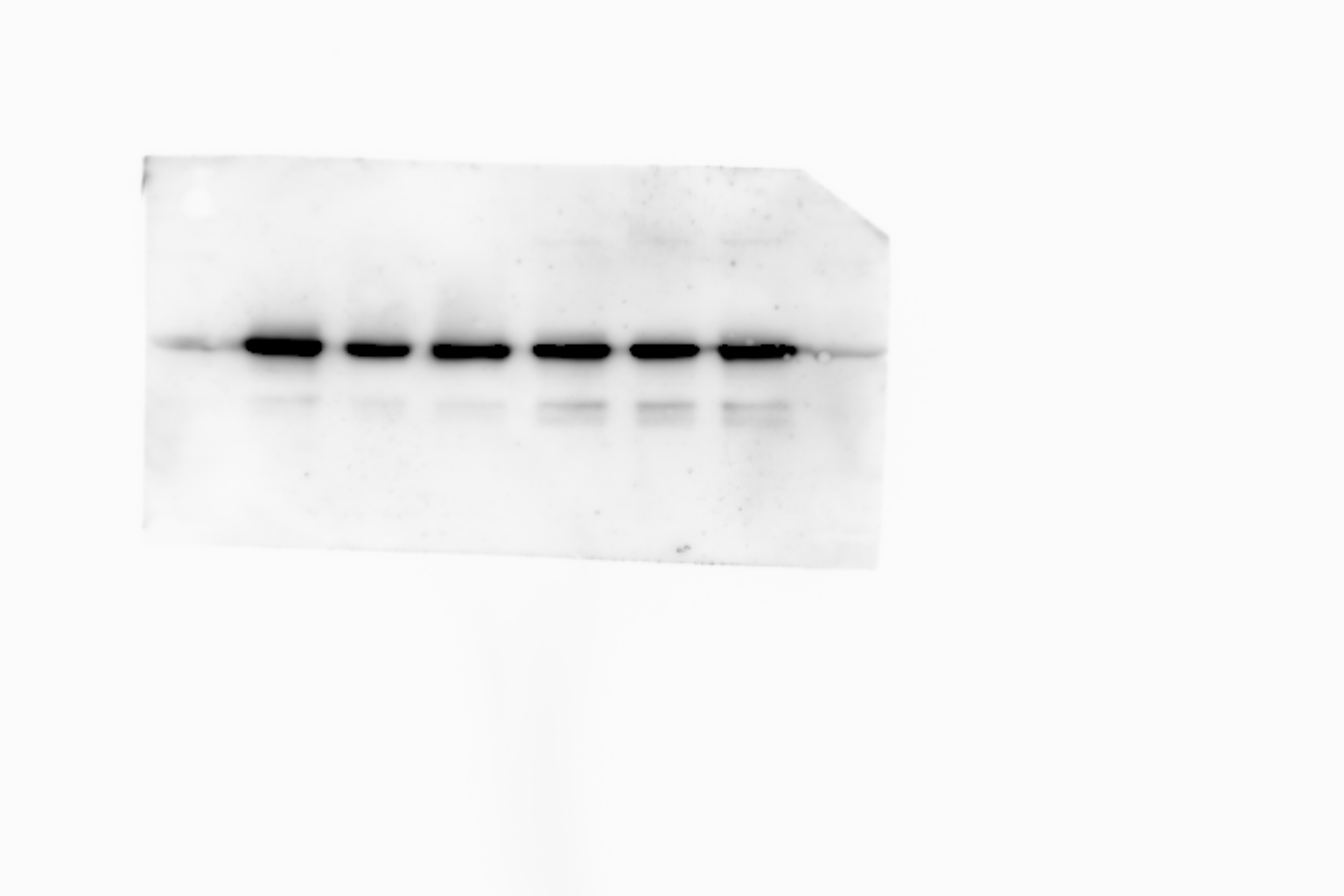

Supplement: Supplementary file 5 — Source data Fig. 3 [file 44321_2025_216_MOESM5_ESM.zip › Figure 3/Figure 3G/Inguinal fat/akt/akt.tif]

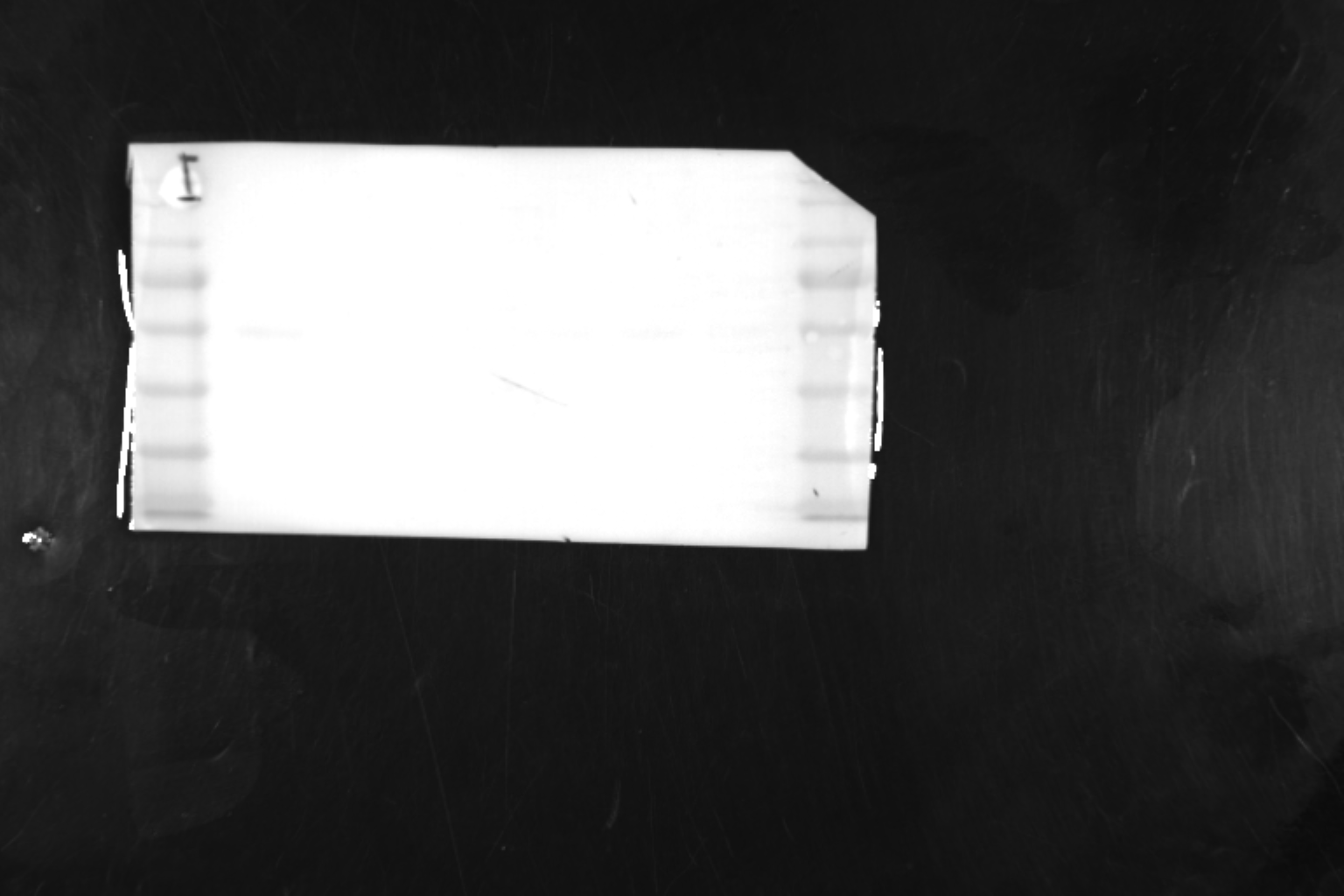

Supplement: Supplementary file 5 — Source data Fig. 3 [file 44321_2025_216_MOESM5_ESM.zip › Figure 3/Figure 3G/Inguinal fat/akt/marker.tif]

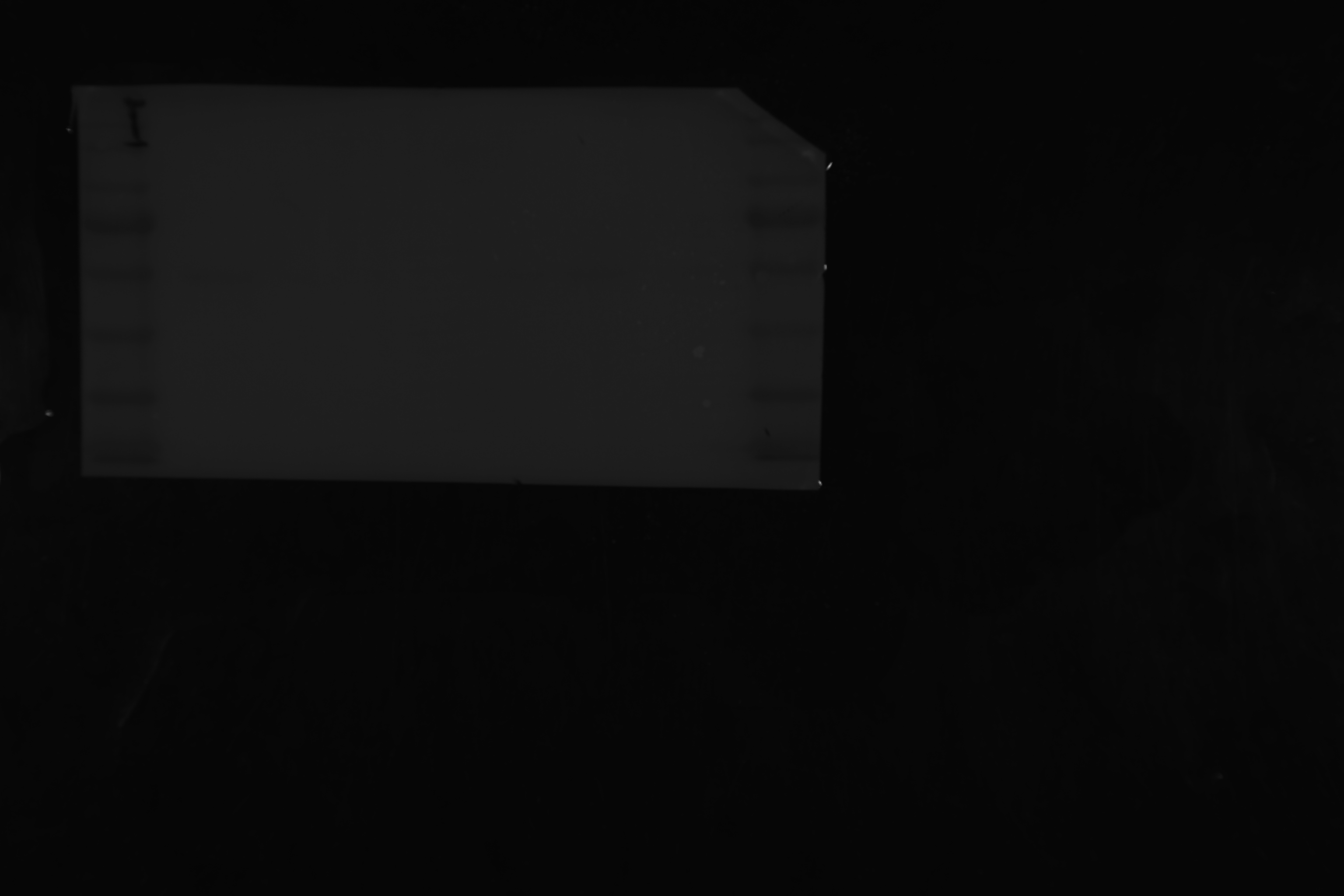

Supplement: Supplementary file 5 — Source data Fig. 3 [file 44321_2025_216_MOESM5_ESM.zip › Figure 3/Figure 3G/Inguinal fat/pAkt/marker.tif]

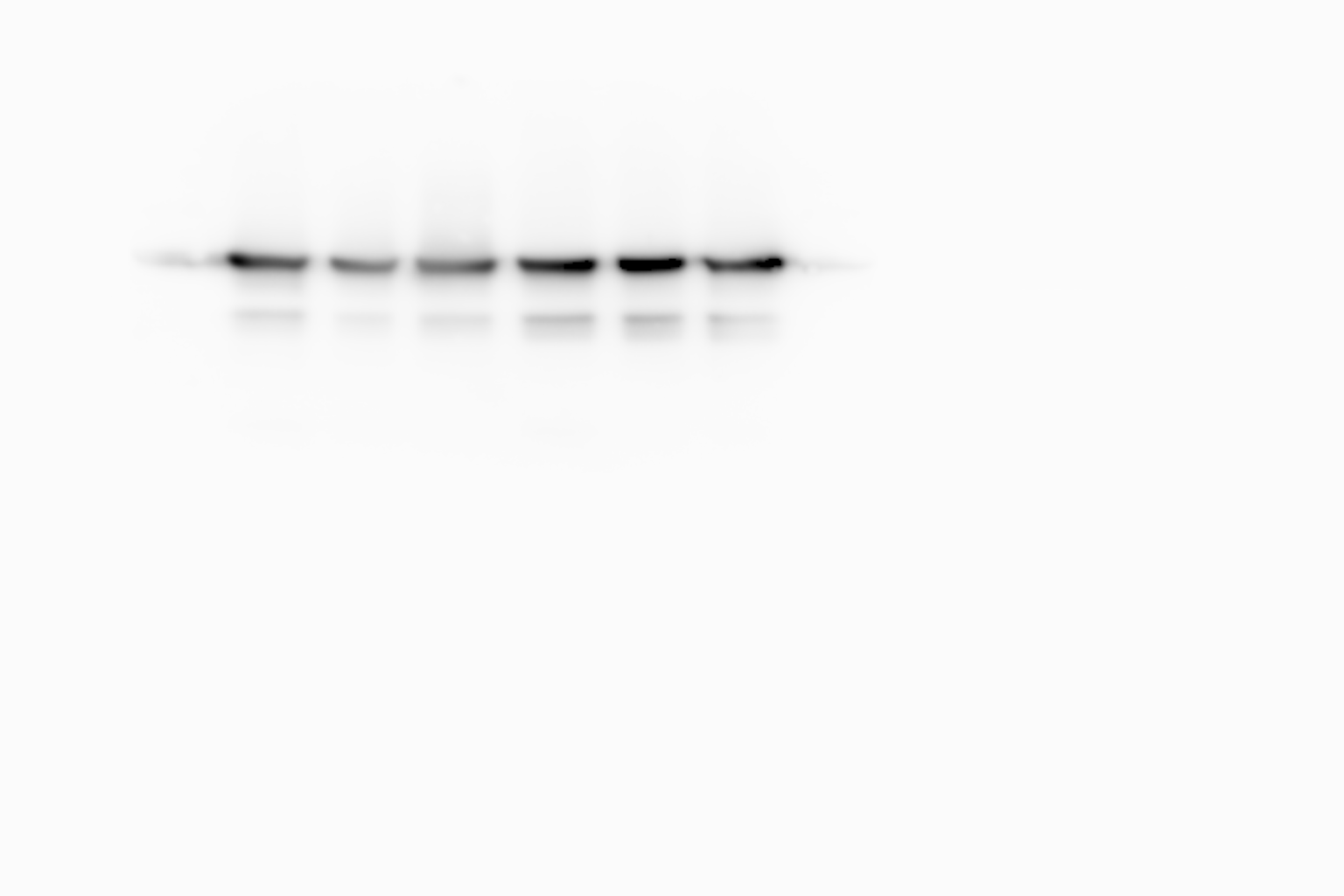

Supplement: Supplementary file 5 — Source data Fig. 3 [file 44321_2025_216_MOESM5_ESM.zip › Figure 3/Figure 3G/Inguinal fat/pAkt/pAkt.tif]

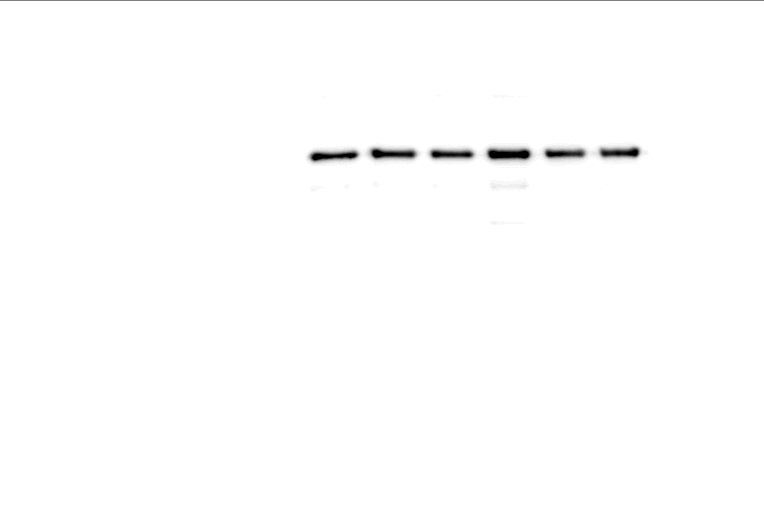

Supplement: Supplementary file 5 — Source data Fig. 3 [file 44321_2025_216_MOESM5_ESM.zip › Figure 3/Figure 3G/Liver/akt/akt.tif]

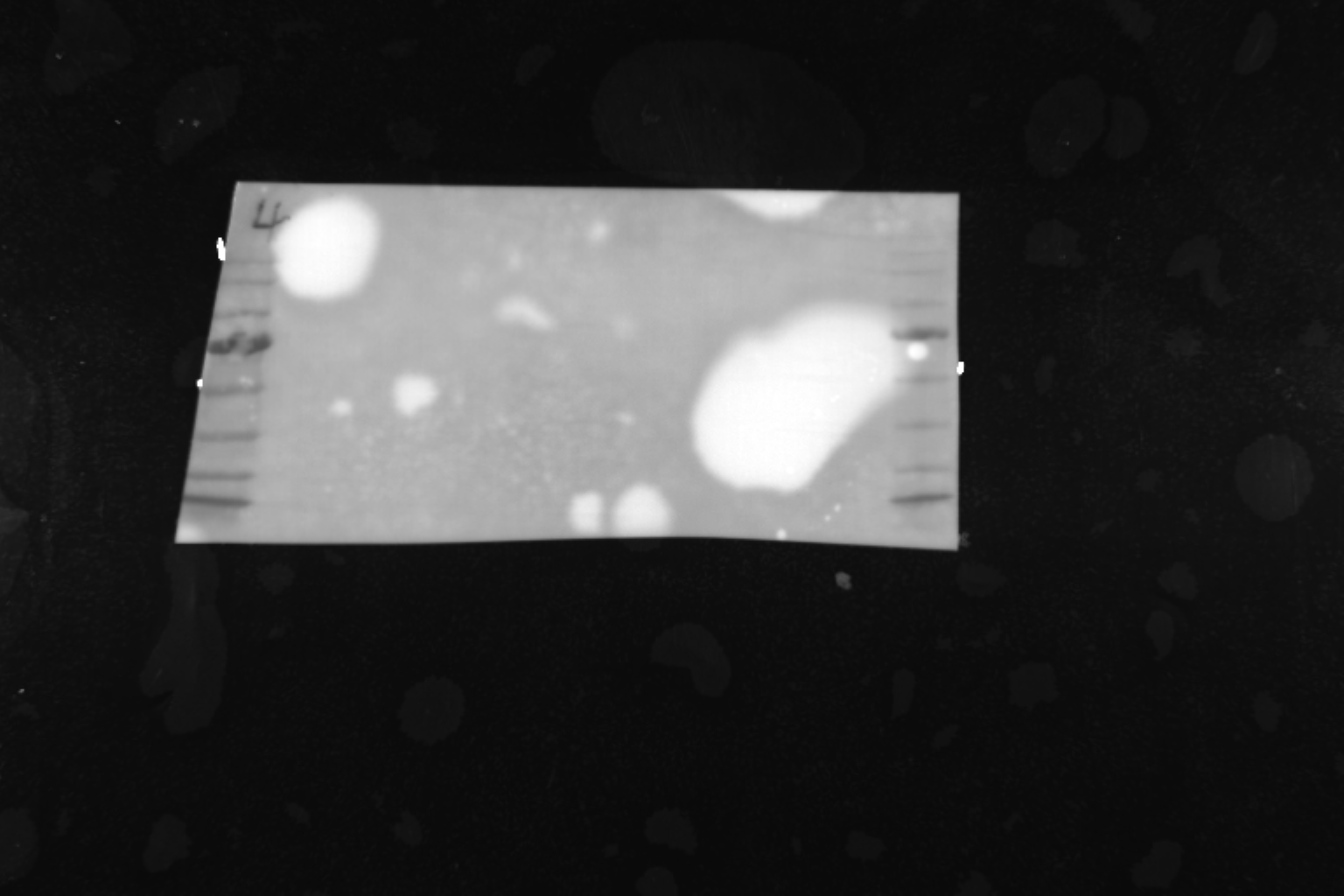

Supplement: Supplementary file 5 — Source data Fig. 3 [file 44321_2025_216_MOESM5_ESM.zip › Figure 3/Figure 3G/Liver/akt/marker.tif]

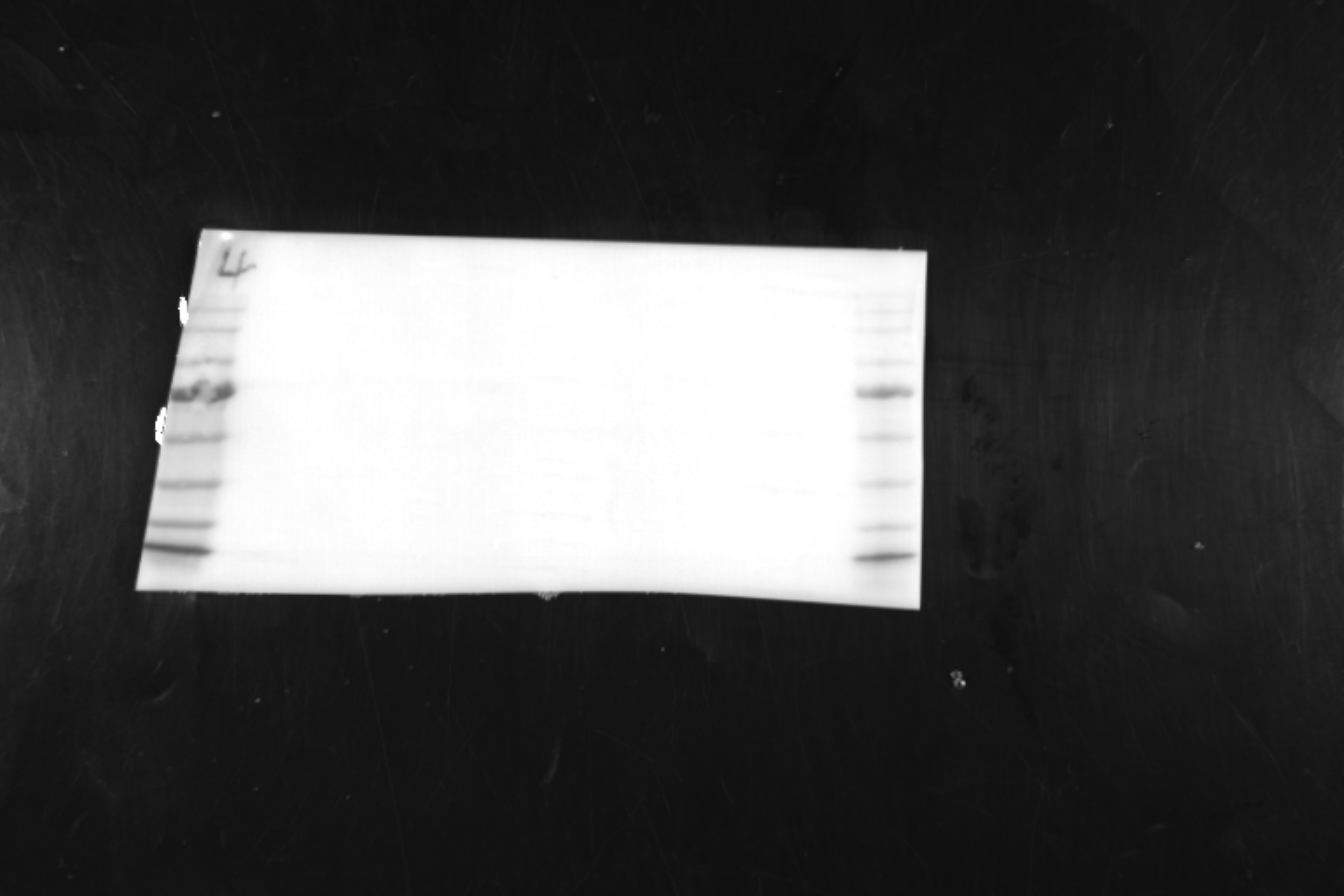

Supplement: Supplementary file 5 — Source data Fig. 3 [file 44321_2025_216_MOESM5_ESM.zip › Figure 3/Figure 3G/Liver/pAkt/marker.tif]

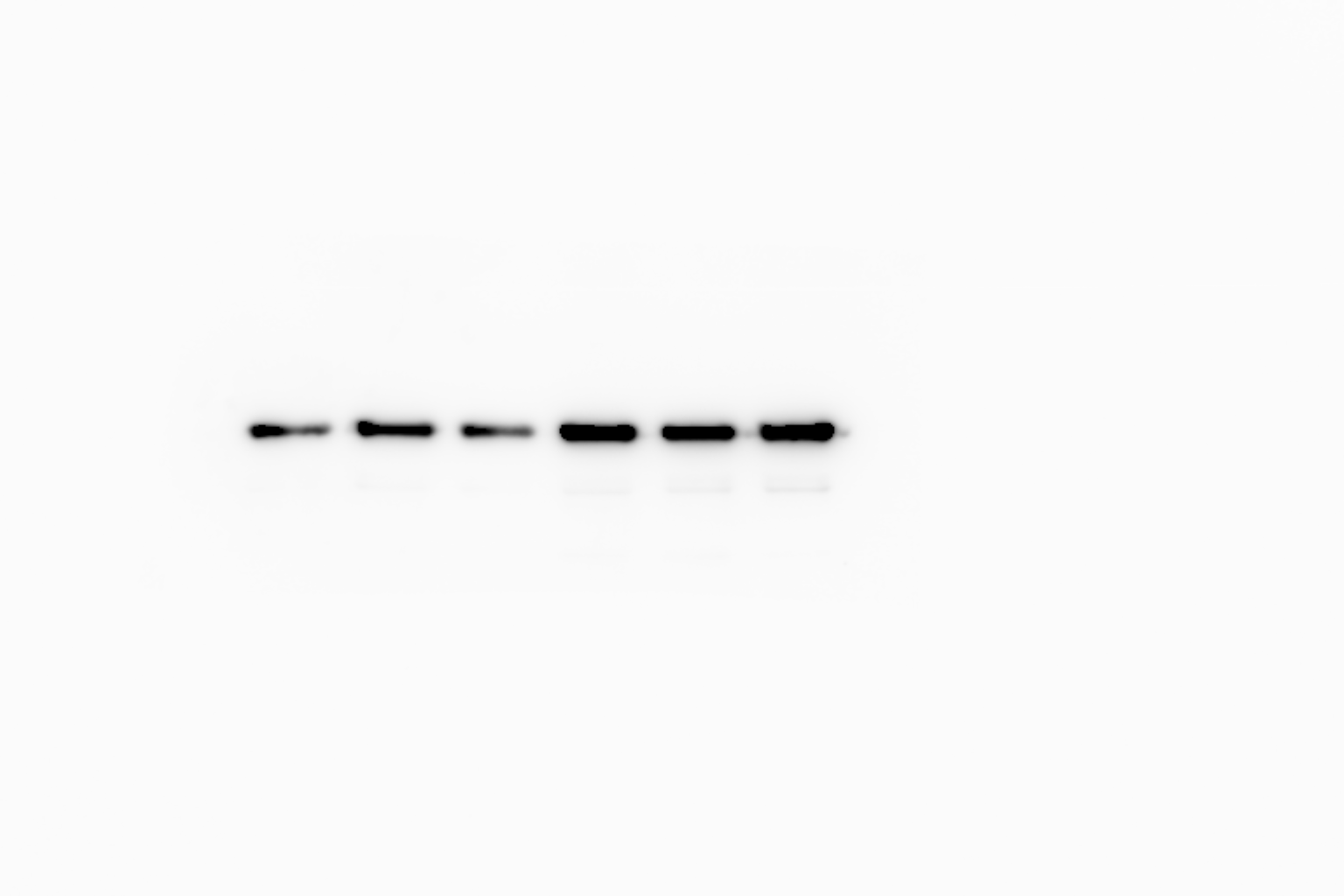

Supplement: Supplementary file 5 — Source data Fig. 3 [file 44321_2025_216_MOESM5_ESM.zip › Figure 3/Figure 3G/Liver/pAkt/Pakt.tif]

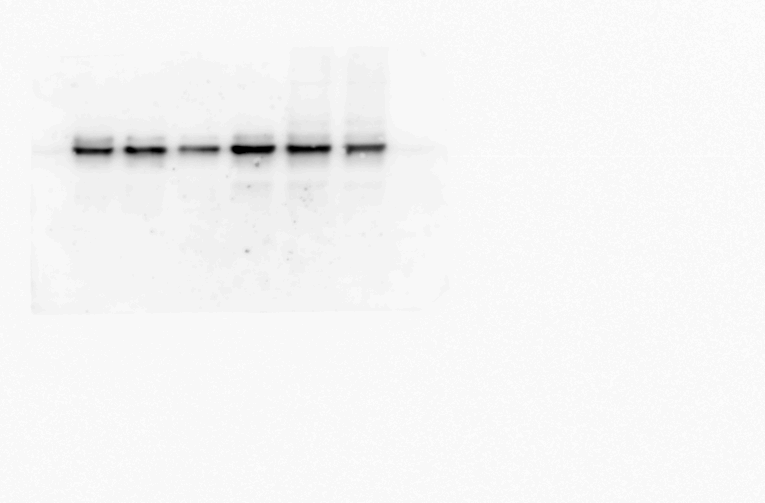

Supplement: Supplementary file 5 — Source data Fig. 3 [file 44321_2025_216_MOESM5_ESM.zip › Figure 3/Figure 3G/Perigonadal fat/akt/akt.tif]

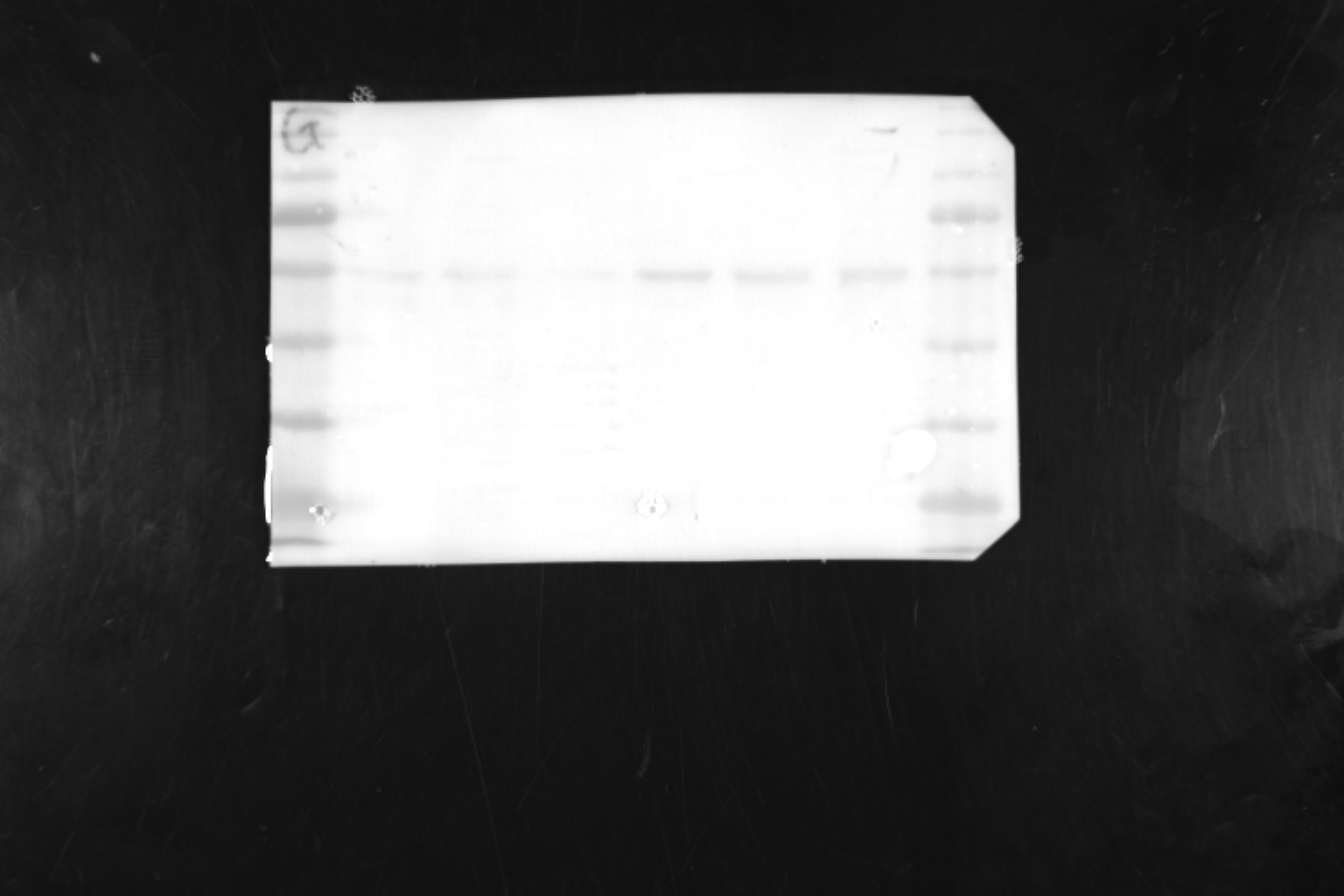

Supplement: Supplementary file 5 — Source data Fig. 3 [file 44321_2025_216_MOESM5_ESM.zip › Figure 3/Figure 3G/Perigonadal fat/akt/marker.tif]

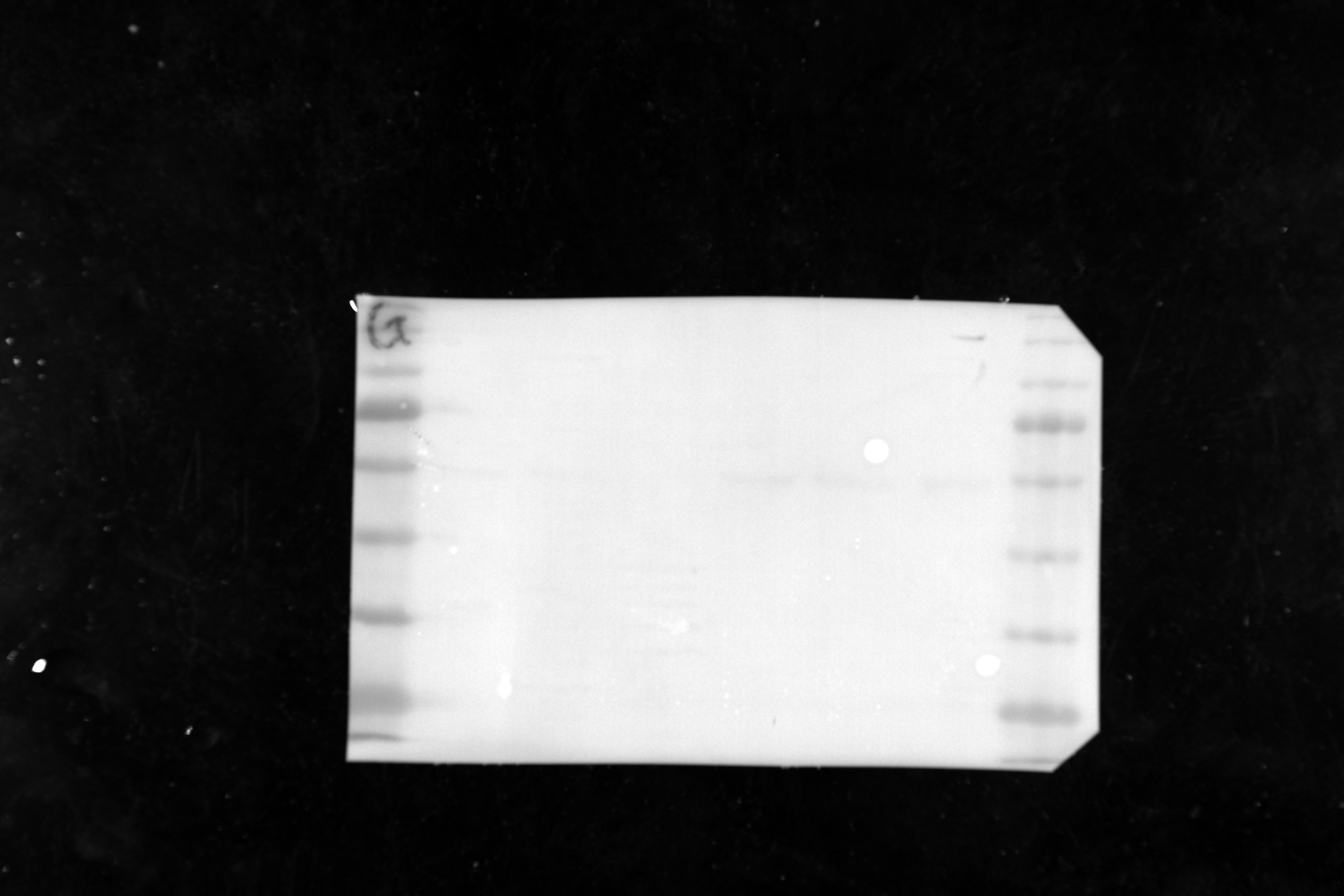

Supplement: Supplementary file 5 — Source data Fig. 3 [file 44321_2025_216_MOESM5_ESM.zip › Figure 3/Figure 3G/Perigonadal fat/pAkt/Marker.tif]

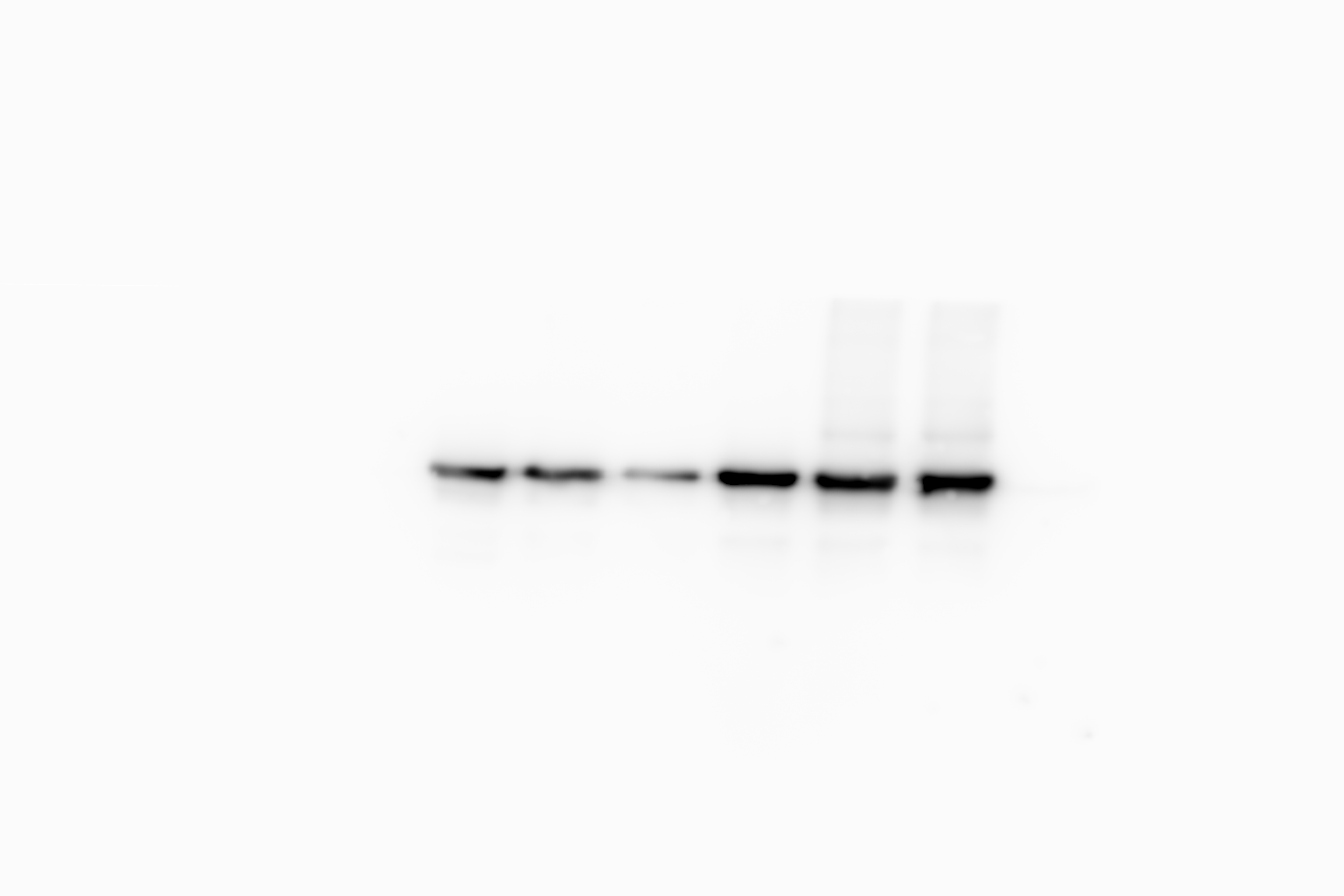

Supplement: Supplementary file 5 — Source data Fig. 3 [file 44321_2025_216_MOESM5_ESM.zip › Figure 3/Figure 3G/Perigonadal fat/pAkt/pAkt.tif]

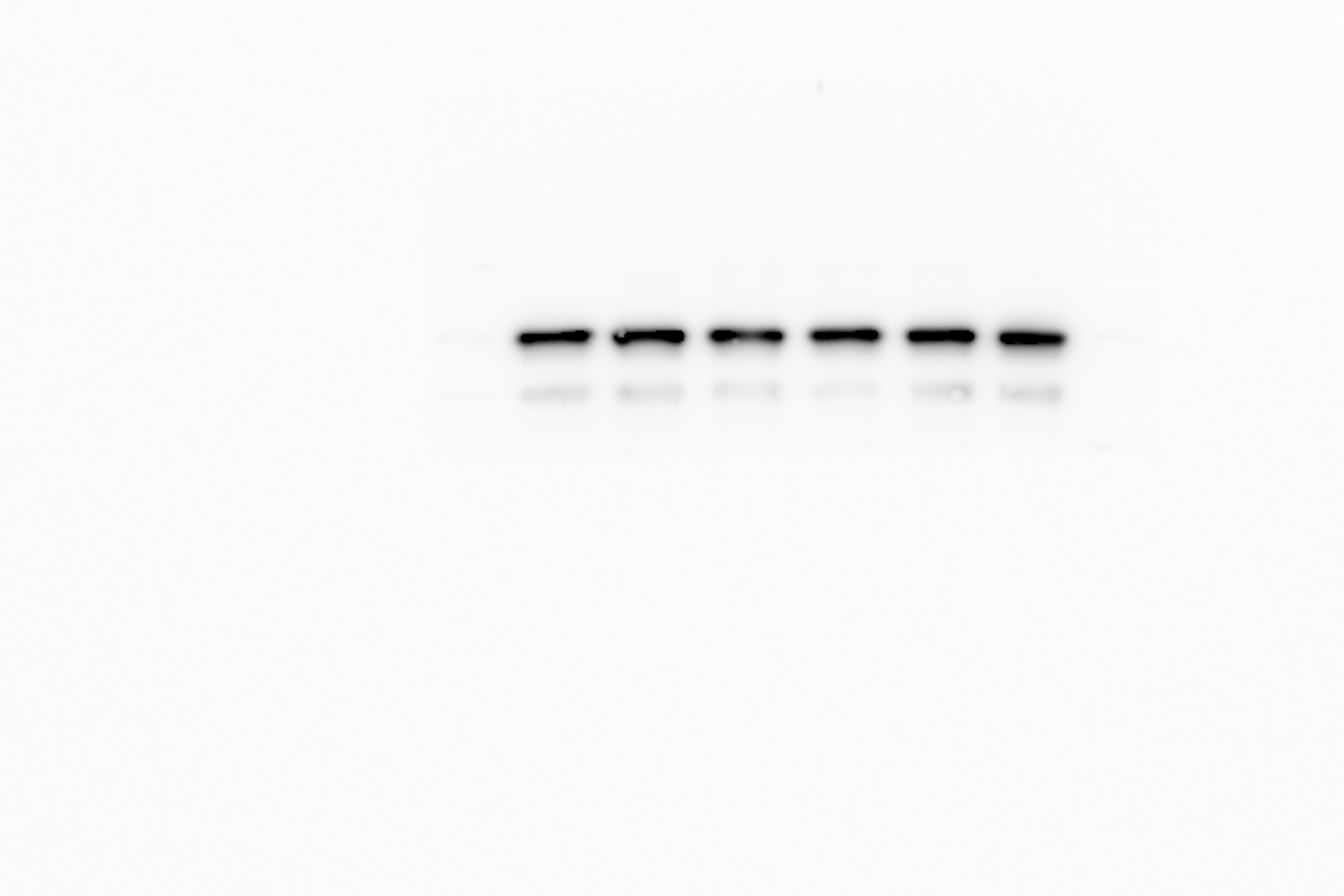

Supplement: Supplementary file 5 — Source data Fig. 3 [file 44321_2025_216_MOESM5_ESM.zip › Figure 3/Figure 3G/Skeletal muscle/akt/Akt.tif]

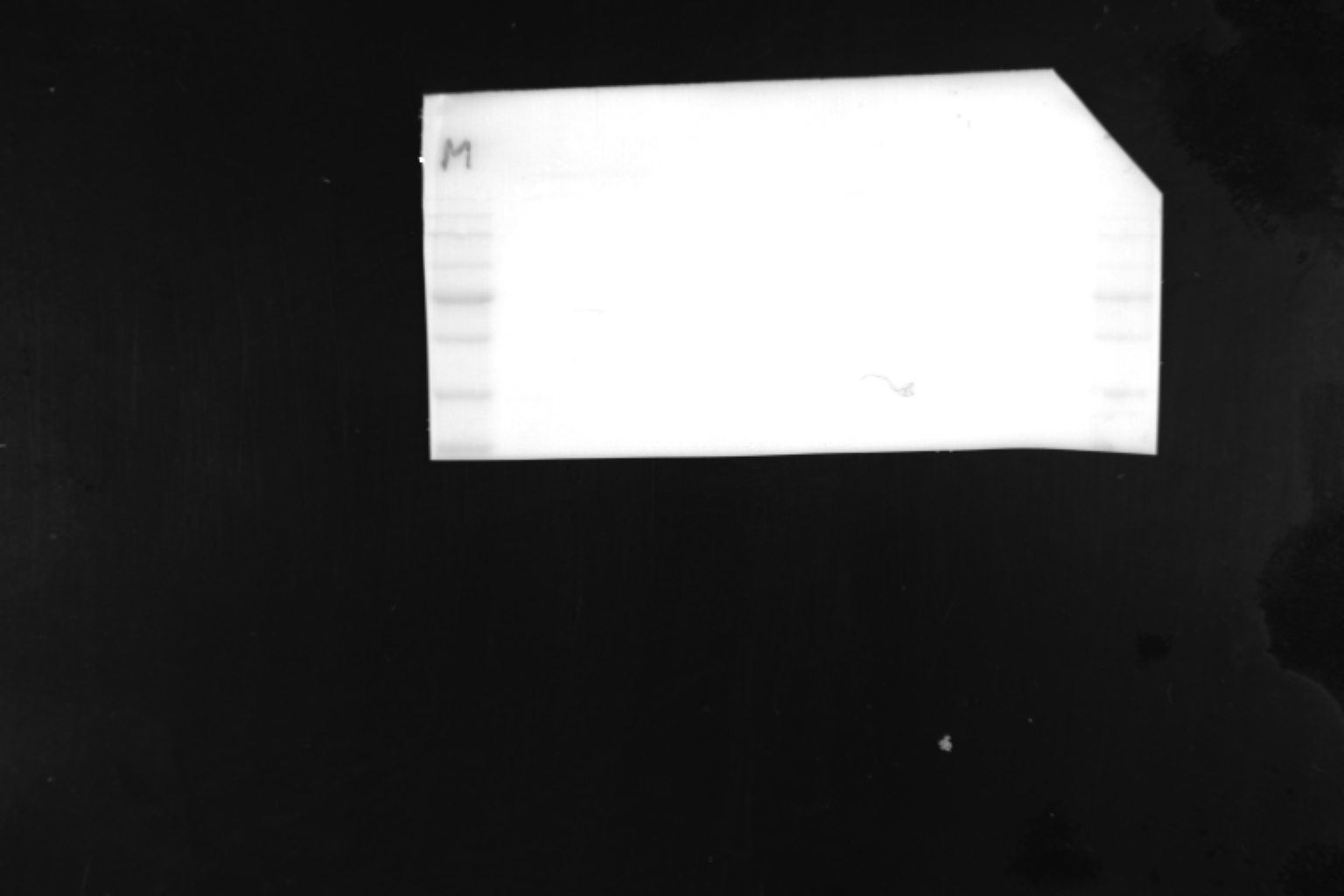

Supplement: Supplementary file 5 — Source data Fig. 3 [file 44321_2025_216_MOESM5_ESM.zip › Figure 3/Figure 3G/Skeletal muscle/akt/Market.tif]

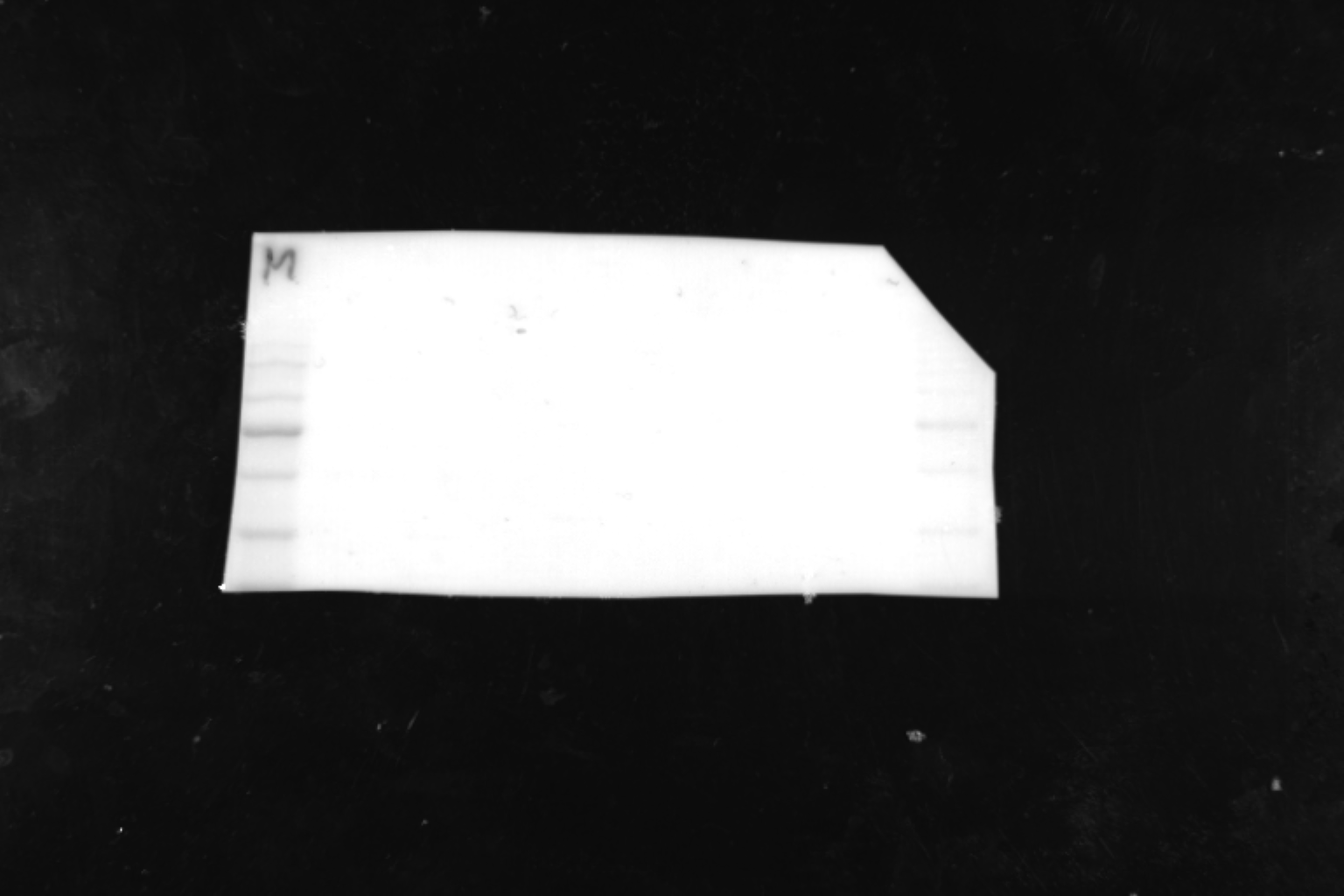

Supplement: Supplementary file 5 — Source data Fig. 3 [file 44321_2025_216_MOESM5_ESM.zip › Figure 3/Figure 3G/Skeletal muscle/pAkt/Marker.tif]

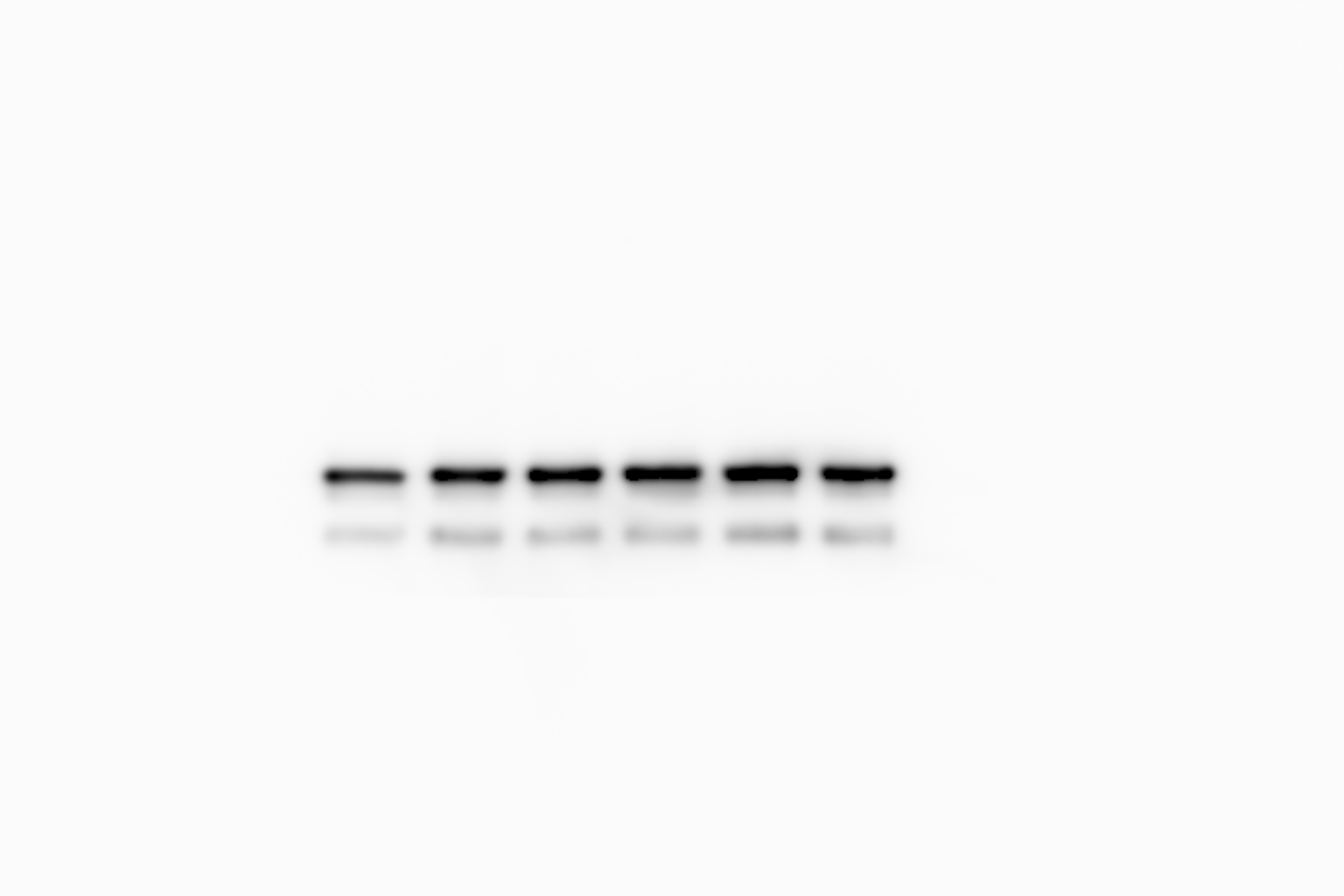

Supplement: Supplementary file 5 — Source data Fig. 3 [file 44321_2025_216_MOESM5_ESM.zip › Figure 3/Figure 3G/Skeletal muscle/pAkt/pAkt.tif]

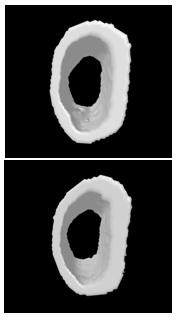

Supplement: Supplementary file 5 — Source data Fig. 3 [file 44321_2025_216_MOESM5_ESM.zip › Figure 3/Figure 3K.jpg]

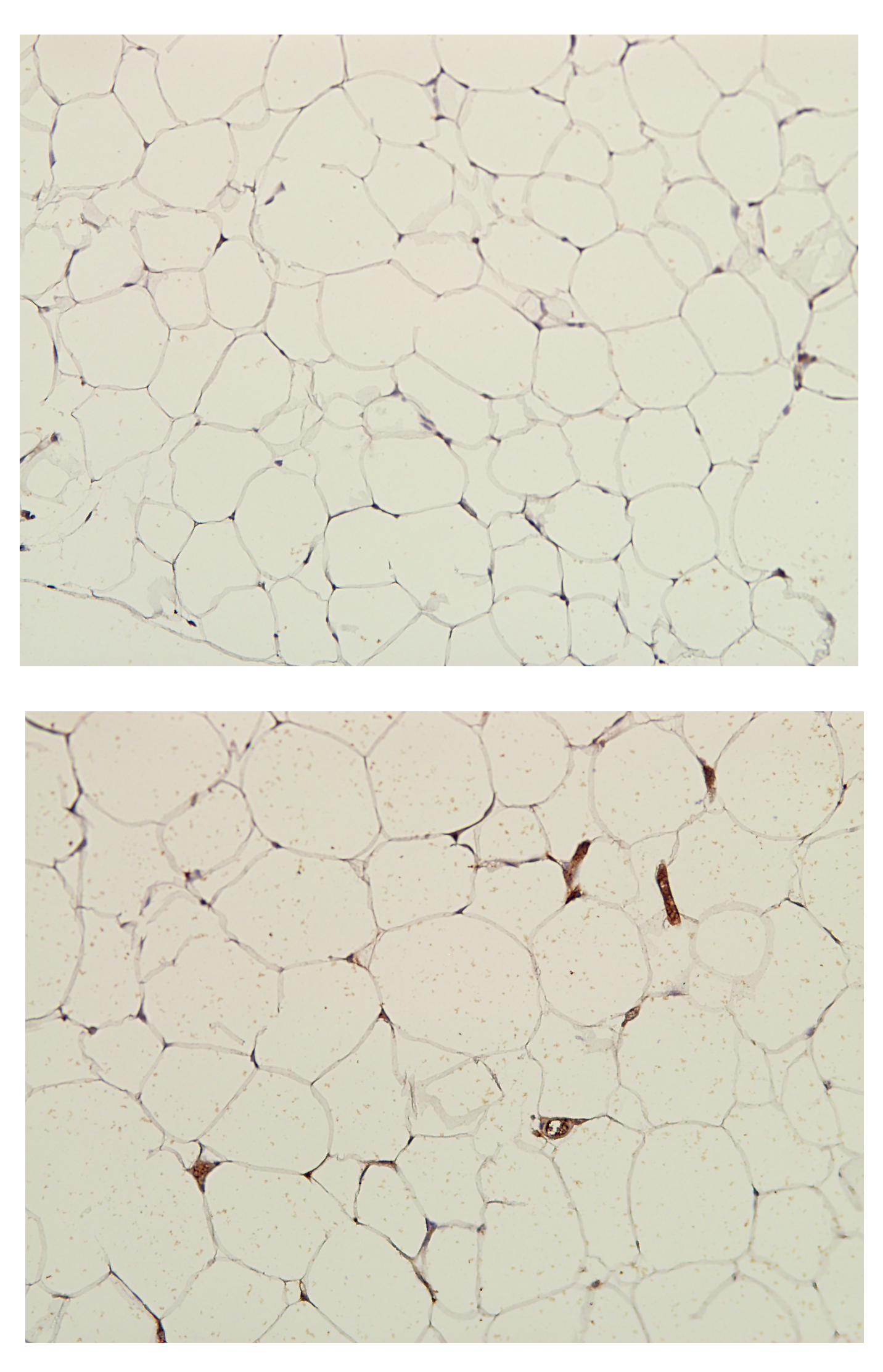

Supplement: Supplementary file 5 — Source data Fig. 3 [file 44321_2025_216_MOESM5_ESM.zip › Figure 3/Figure 3M.tif]

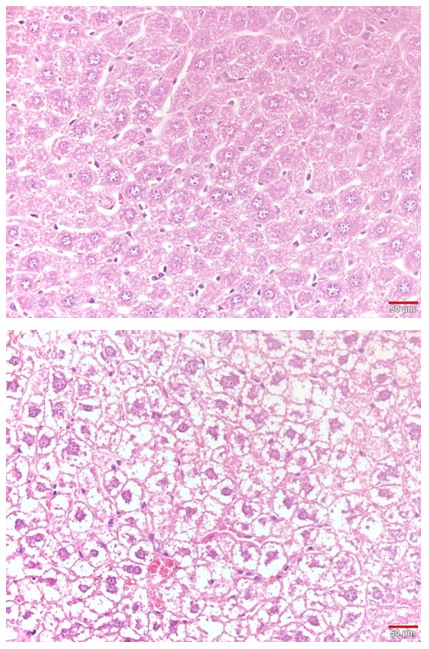

Supplement: Supplementary file 5 — Source data Fig. 3 [file 44321_2025_216_MOESM5_ESM.zip › Figure 3/Figure 3O.jpg]

## Slide 1
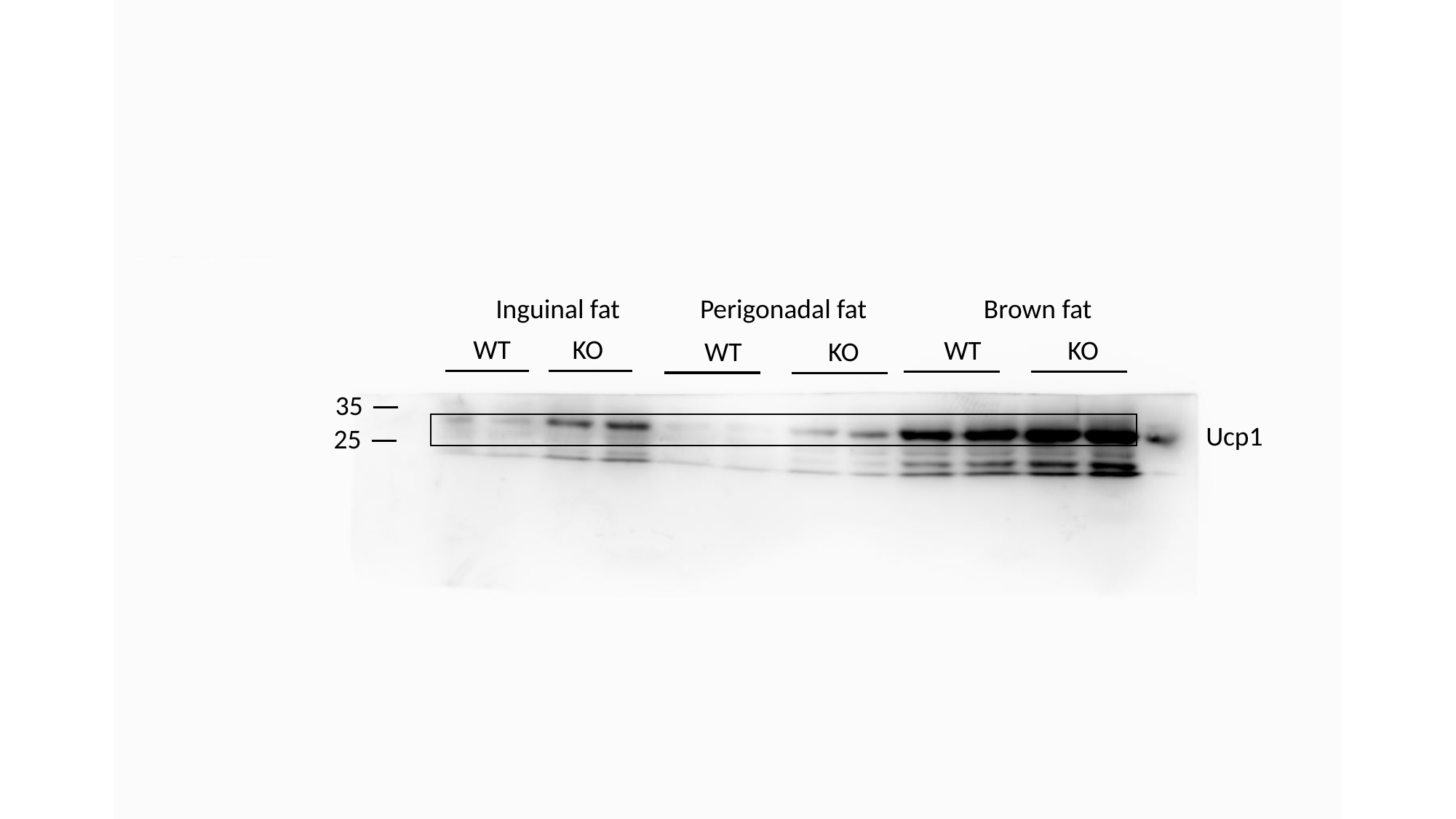

Inguinal fat Perigonadal fat Brown fat
WT KO
 WT KO
 WT KO
35
Ucp1
25

## Slide 2
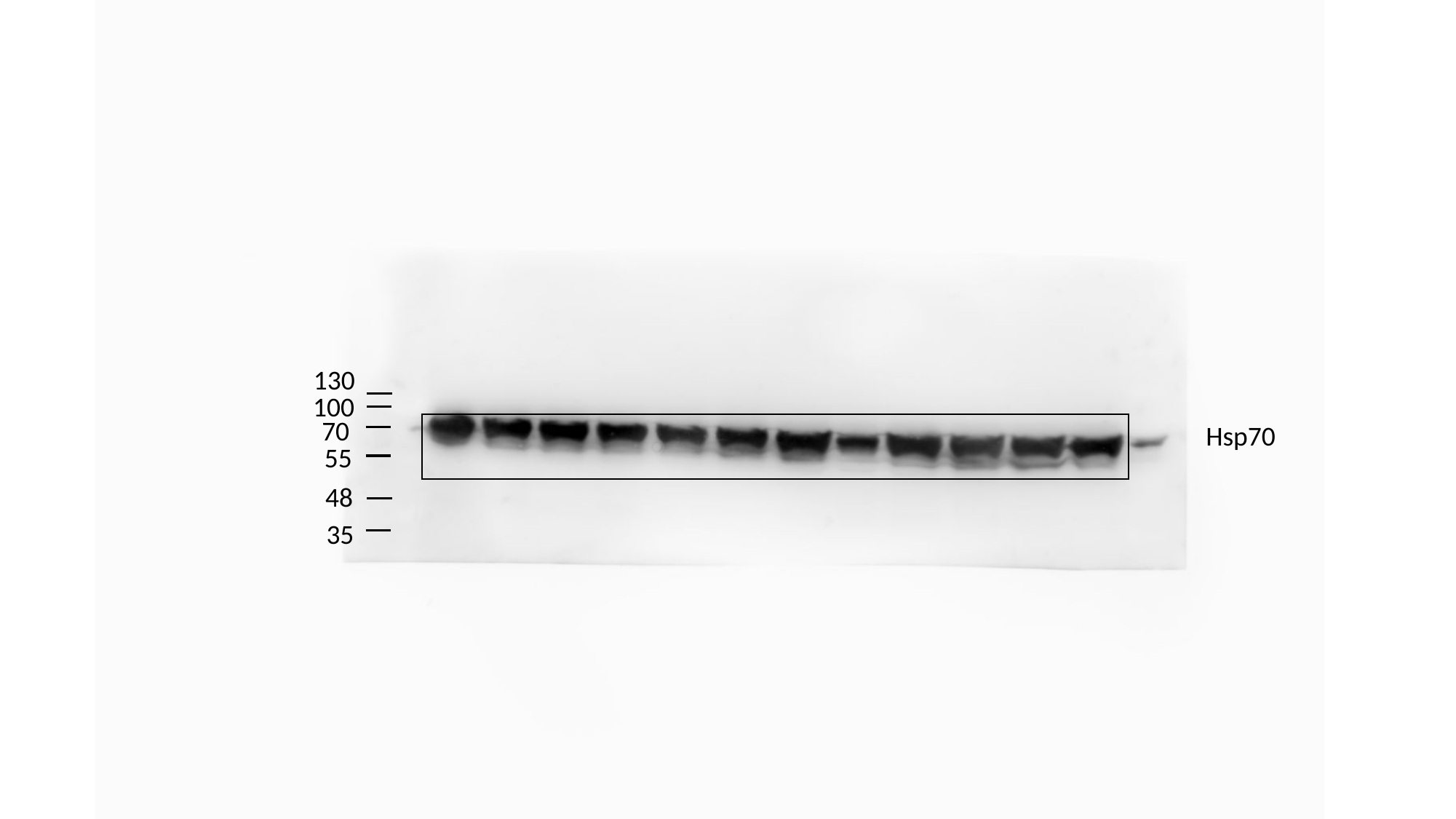

130
100
70
Hsp70
55
48
35

Supplement: Supplementary file 6 — Source data Fig. 4 [file 44321_2025_216_MOESM6_ESM.zip › Figure 4/Figure 4B/Figure 4B.pptx]

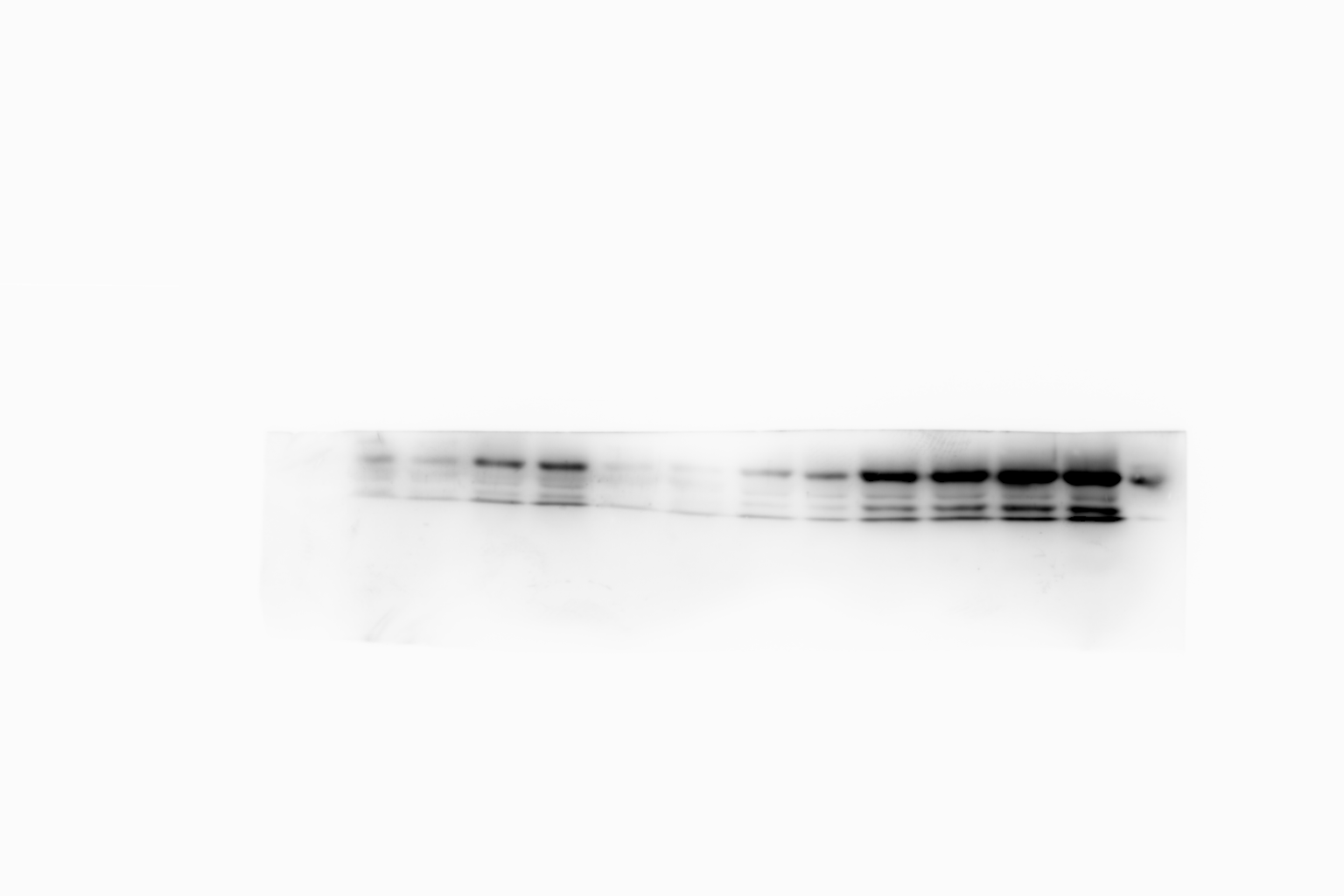

Supplement: Supplementary file 6 — Source data Fig. 4 [file 44321_2025_216_MOESM6_ESM.zip › Figure 4/Figure 4B/Ucp1/PGR2 inguinal gonadal fat BAT UCP1 expression (UCP1) long exposure.tif]

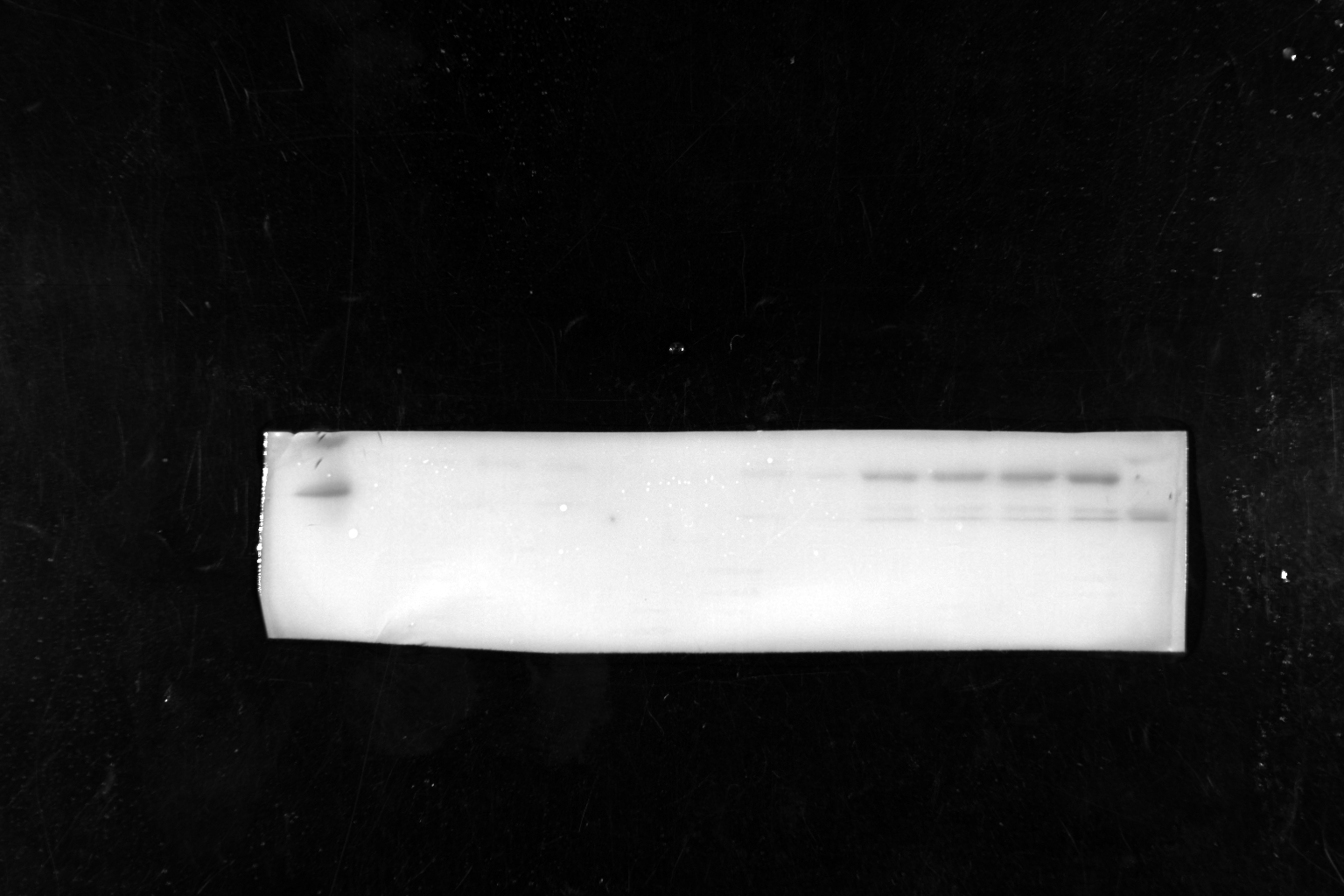

Supplement: Supplementary file 6 — Source data Fig. 4 [file 44321_2025_216_MOESM6_ESM.zip › Figure 4/Figure 4B/Ucp1/20170102_1511.tif]

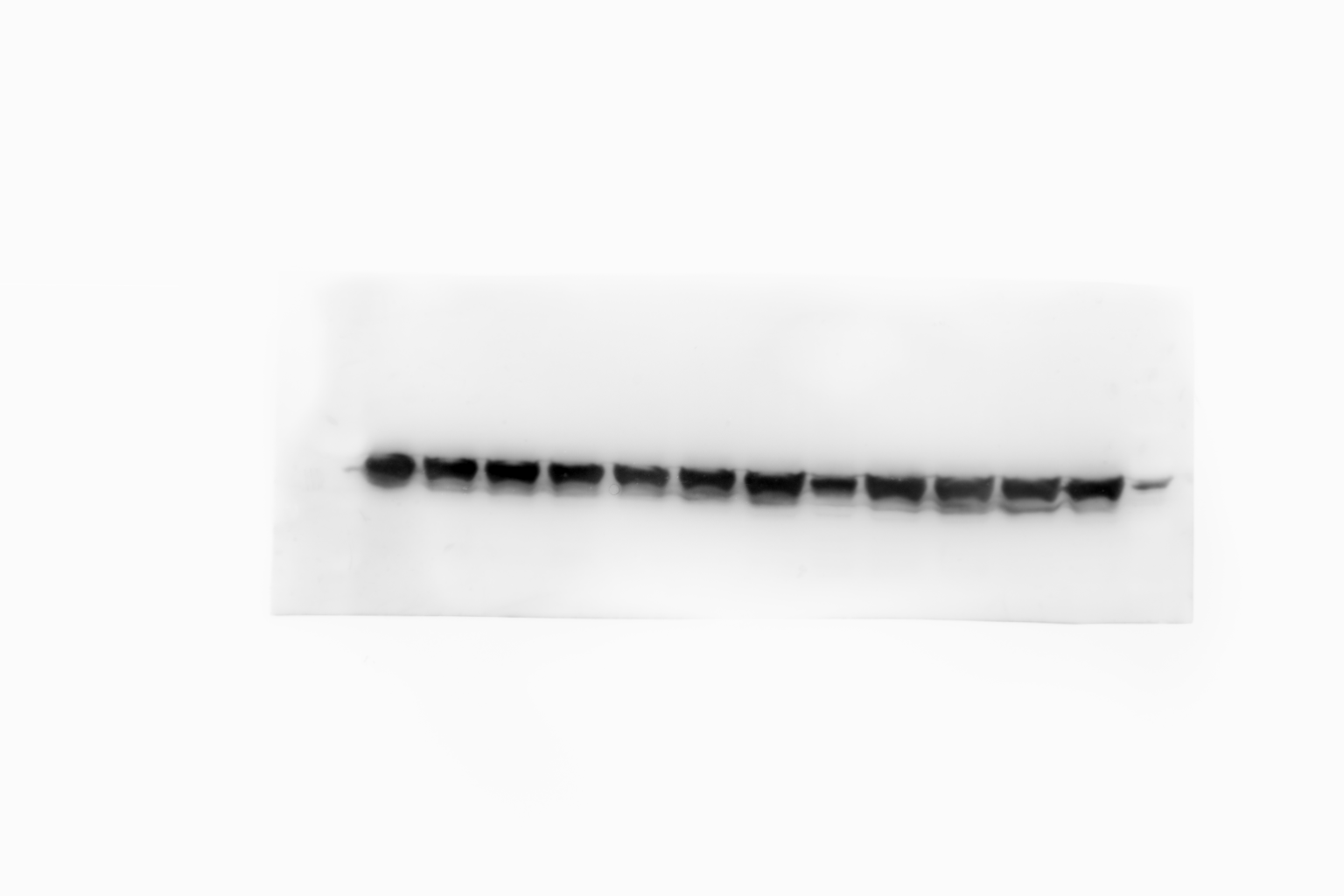

Supplement: Supplementary file 6 — Source data Fig. 4 [file 44321_2025_216_MOESM6_ESM.zip › Figure 4/Figure 4B/Hsp70/PGR2 inguinal gonadal fat BAT UCP1 expression (HSP70).tif]

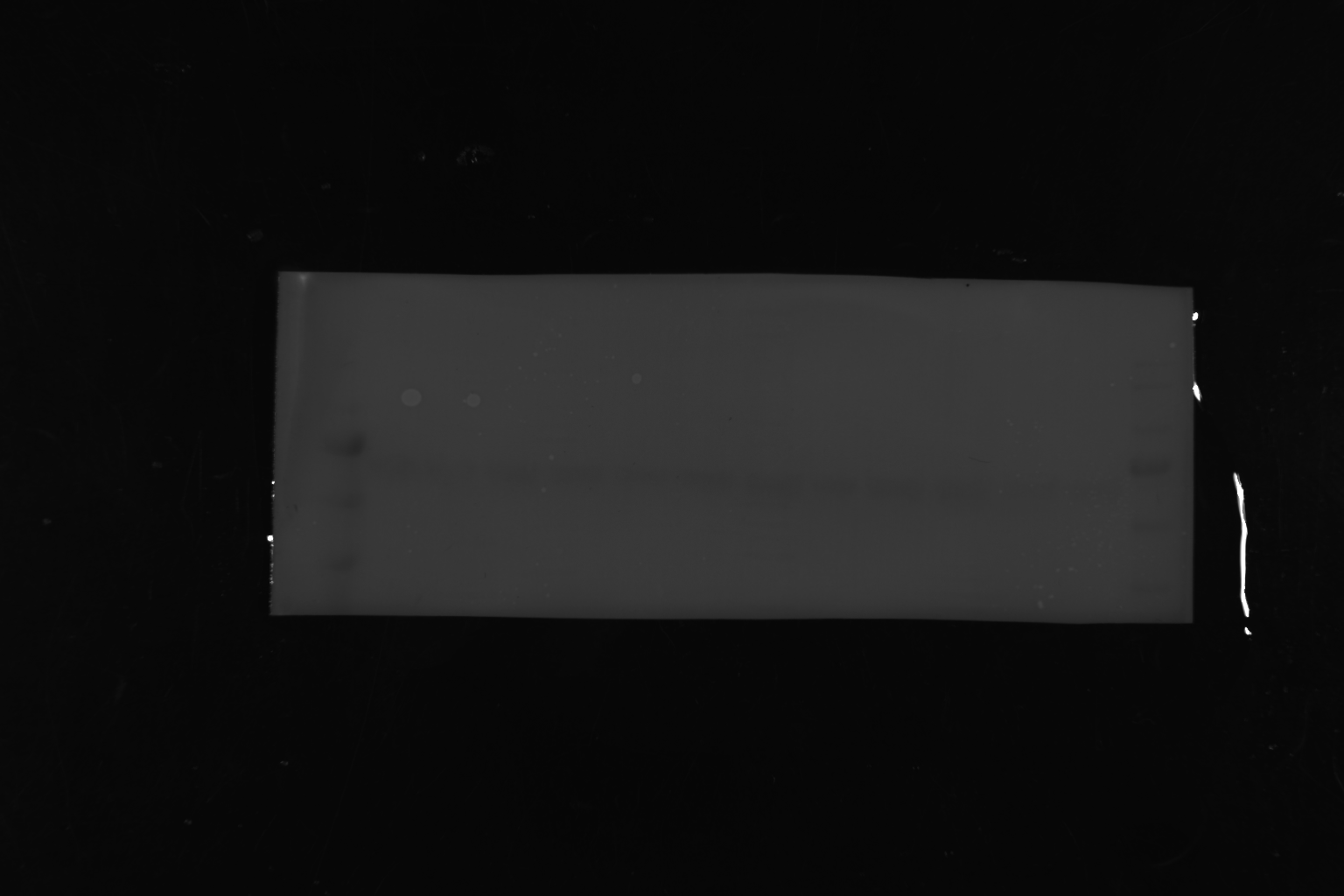

Supplement: Supplementary file 6 — Source data Fig. 4 [file 44321_2025_216_MOESM6_ESM.zip › Figure 4/Figure 4B/Hsp70/PGR2 inguinal gonadal fat BAT UCP1 expression marker (HSP70).tif]

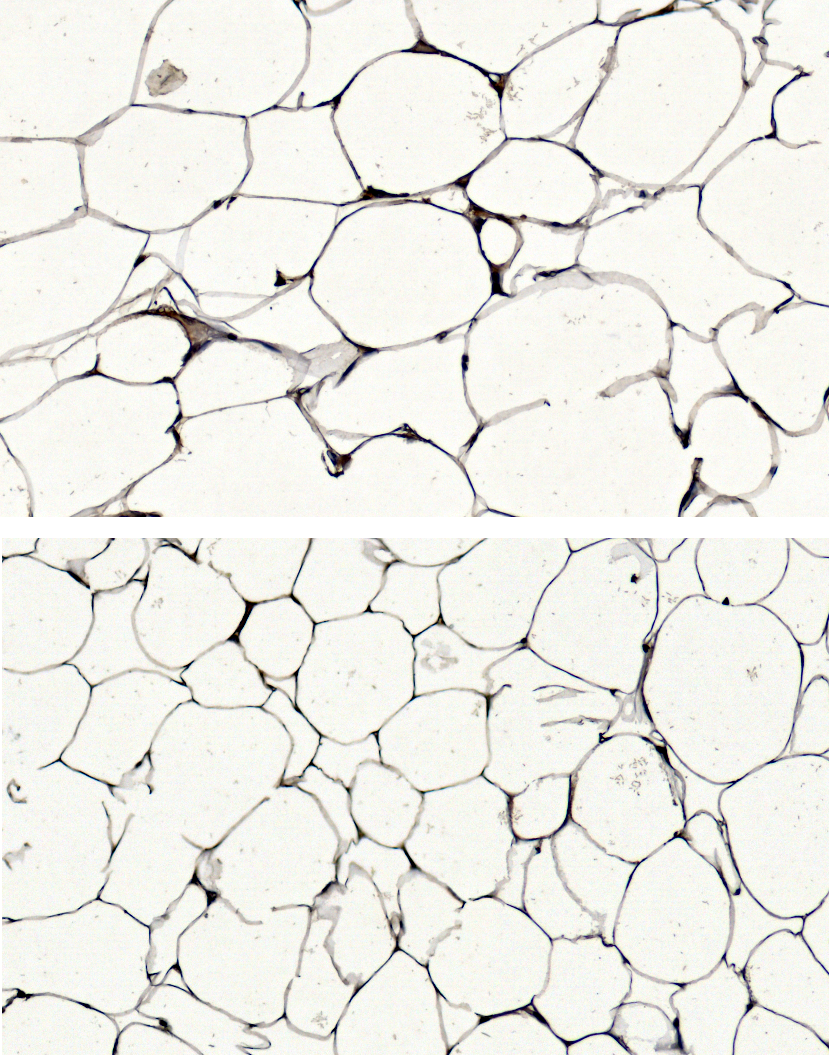

Supplement: Supplementary file 7 — Source data Fig. 5 [file 44321_2025_216_MOESM7_ESM.zip › Figure 5/Figure 5P.tif]

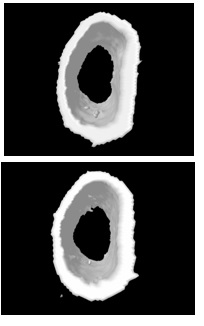

Supplement: Supplementary file 7 — Source data Fig. 5 [file 44321_2025_216_MOESM7_ESM.zip › Figure 5/Figure 5S.jpg]

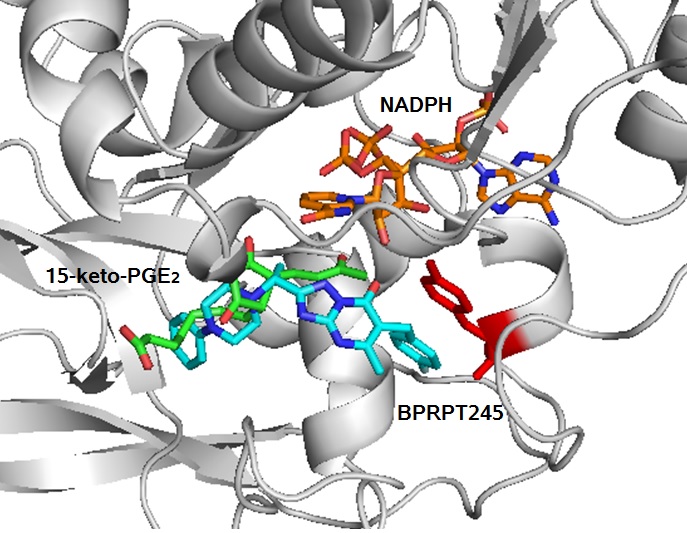

Supplement: Supplementary file 7 — Source data Fig. 5 [file 44321_2025_216_MOESM7_ESM.zip › Figure 5/Figure 5F.jpg]

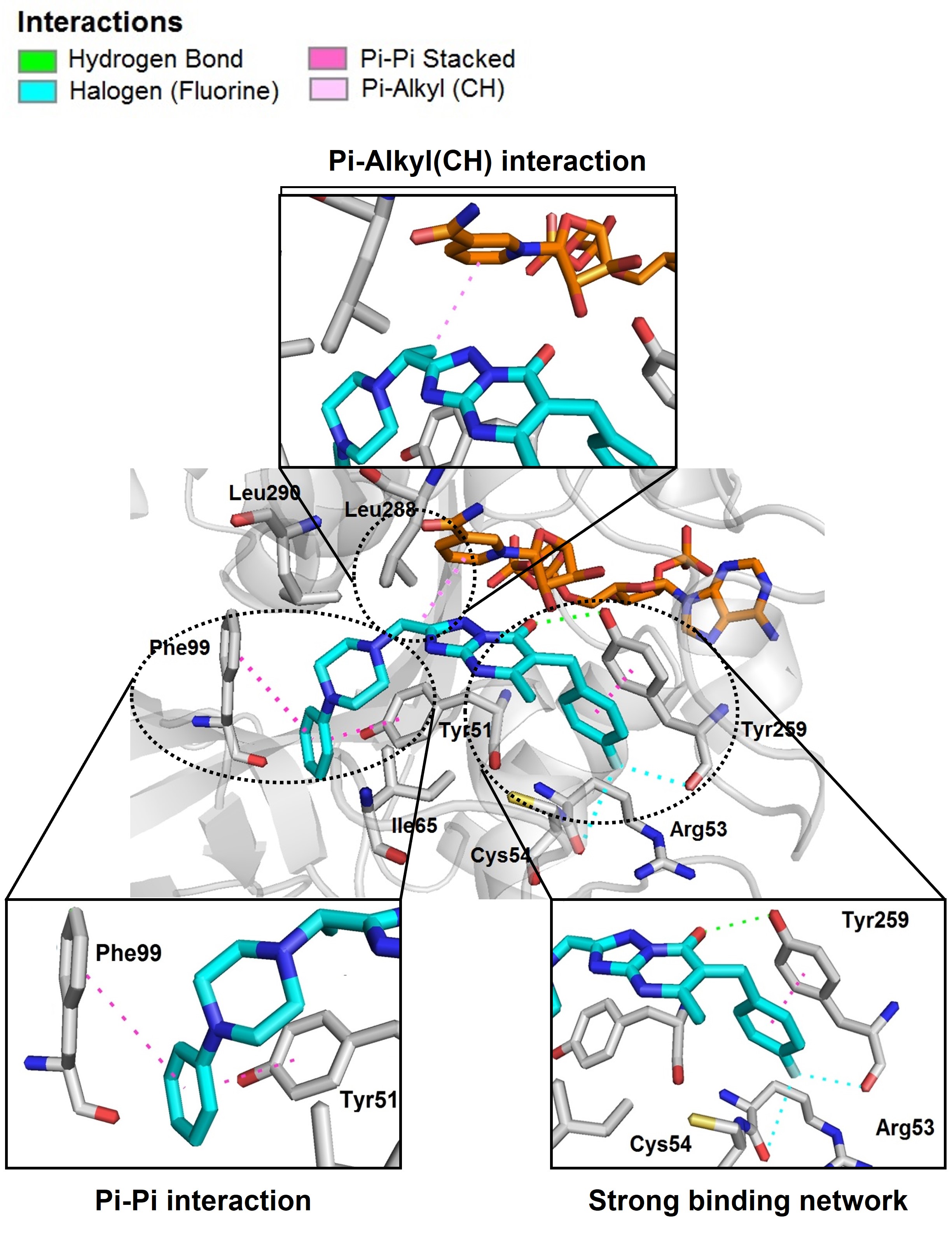

Supplement: Supplementary file 7 — Source data Fig. 5 [file 44321_2025_216_MOESM7_ESM.zip › Figure 5/Figure 5G.jpg]

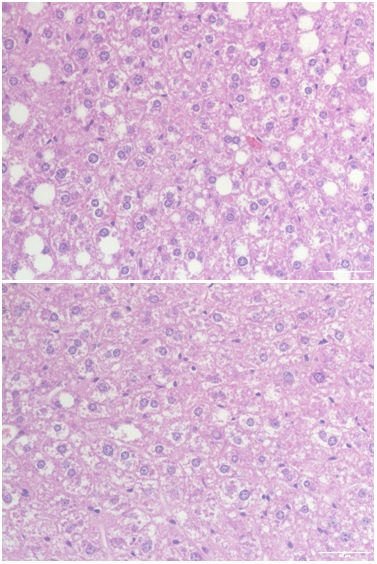

Supplement: Supplementary file 7 — Source data Fig. 5 [file 44321_2025_216_MOESM7_ESM.zip › Figure 5/Figure 5U.jpg]

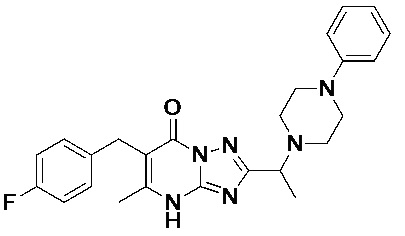

Supplement: Supplementary file 7 — Source data Fig. 5 [file 44321_2025_216_MOESM7_ESM.zip › Figure 5/Figure 5A.jpg]

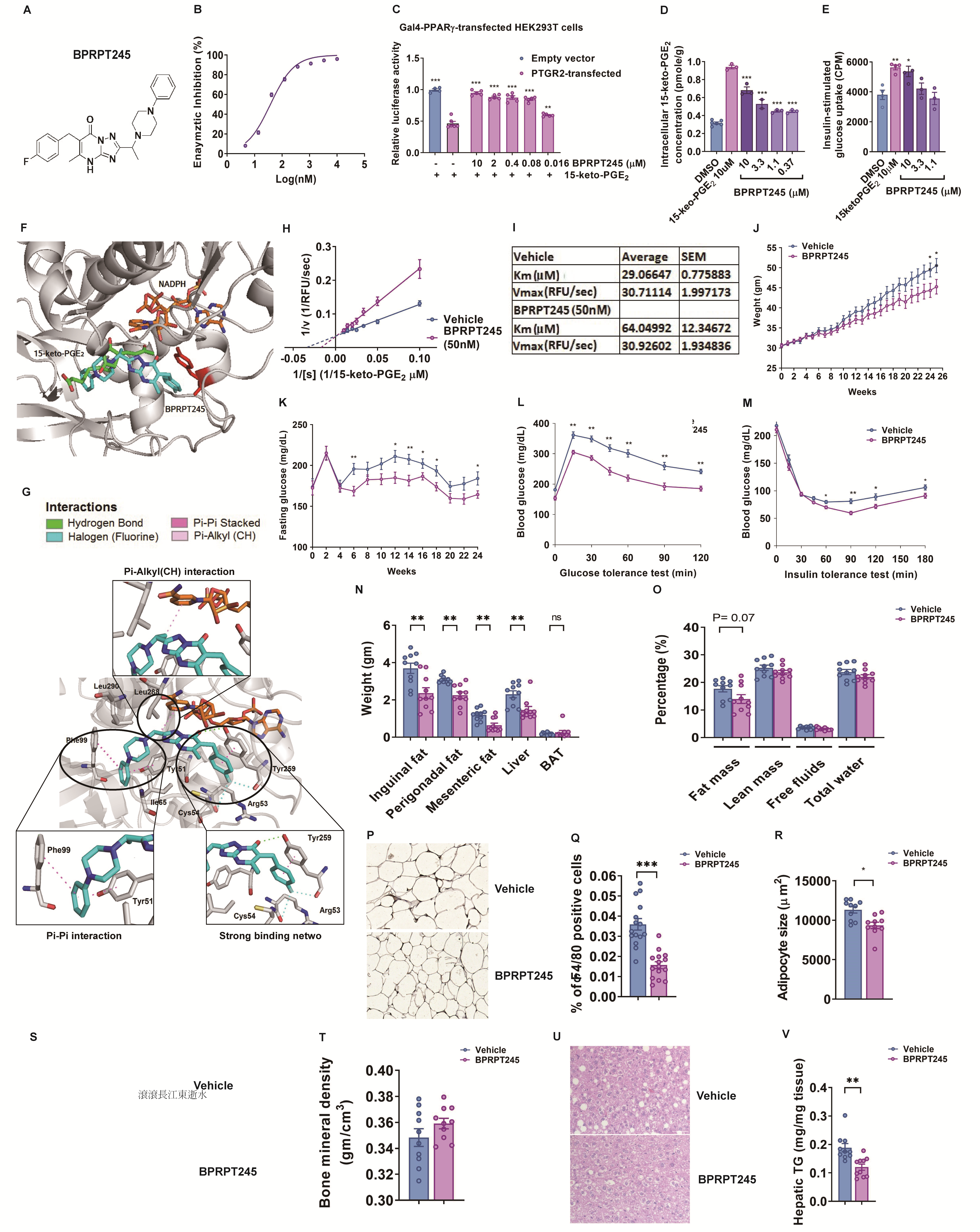

Supplement: Supplementary file 7 — Source data Fig. 5 [file 44321_2025_216_MOESM7_ESM.zip › Figure 5/Figure5.tif]

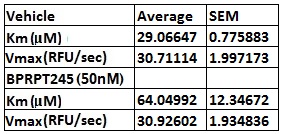

Supplement: Supplementary file 7 — Source data Fig. 5 [file 44321_2025_216_MOESM7_ESM.zip › Figure 5/Figure 5I.jpg]
