# Supplementary material for: Identification of PTGR2 inhibitors as a new therapeutic strategy for diabetes and obesity
Source: EMBO Mol Med. 2025 Mar 21;17(5):938–66. doi: 10.1038/s44321-025-00216-4 (PMC12081876; doi:10.1038/s44321-025-00216-4)
Supplement: Supplementary file 4 — Source data Fig. 2 [file 44321_2025_216_MOESM4_ESM.zip › Figure 2/Figure 2L/Figure 2L.pptx]

## Slide 1
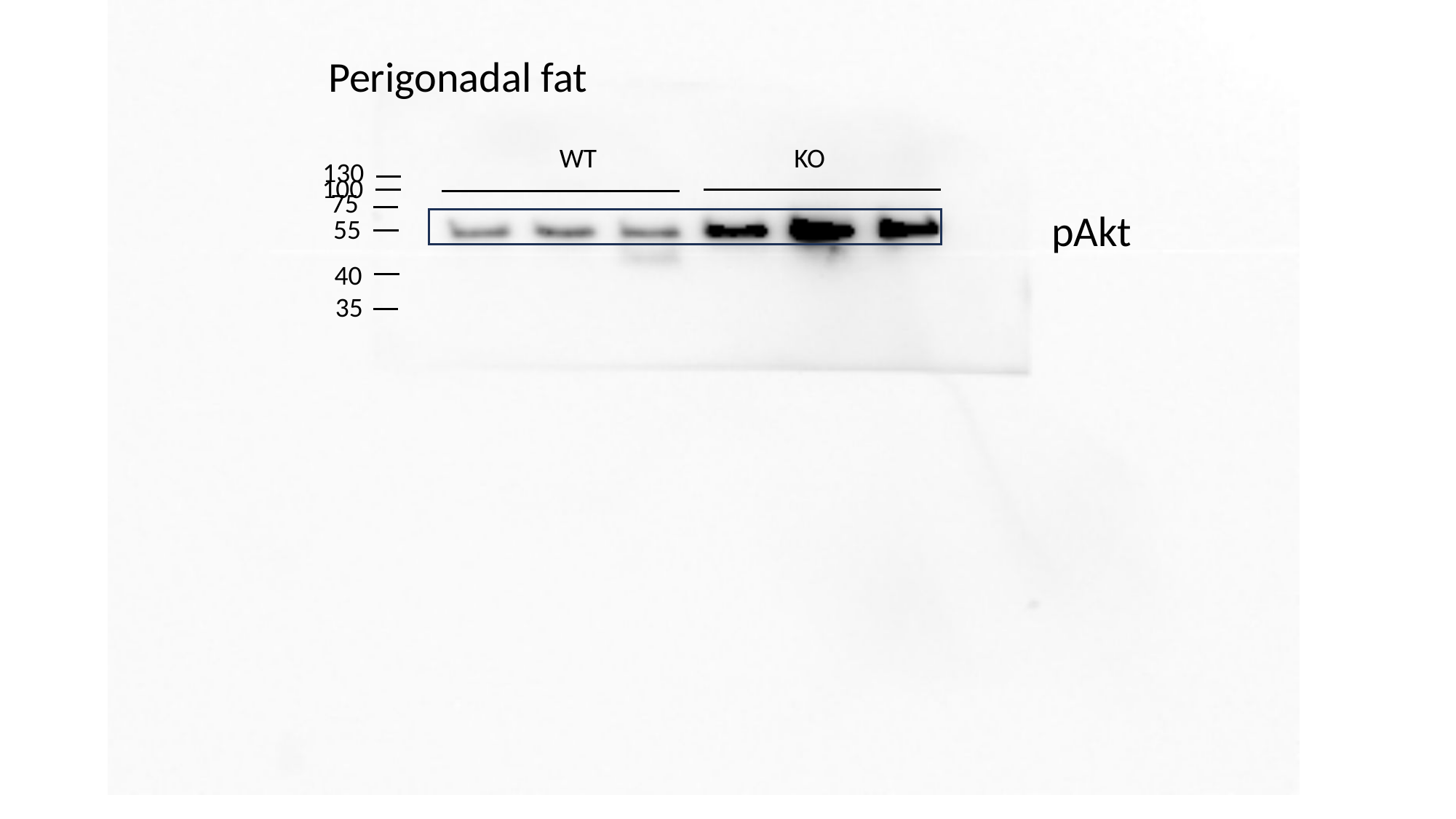

Perigonadal fat
WT KO
130
100
75
pAkt
55
40
35

## Slide 2
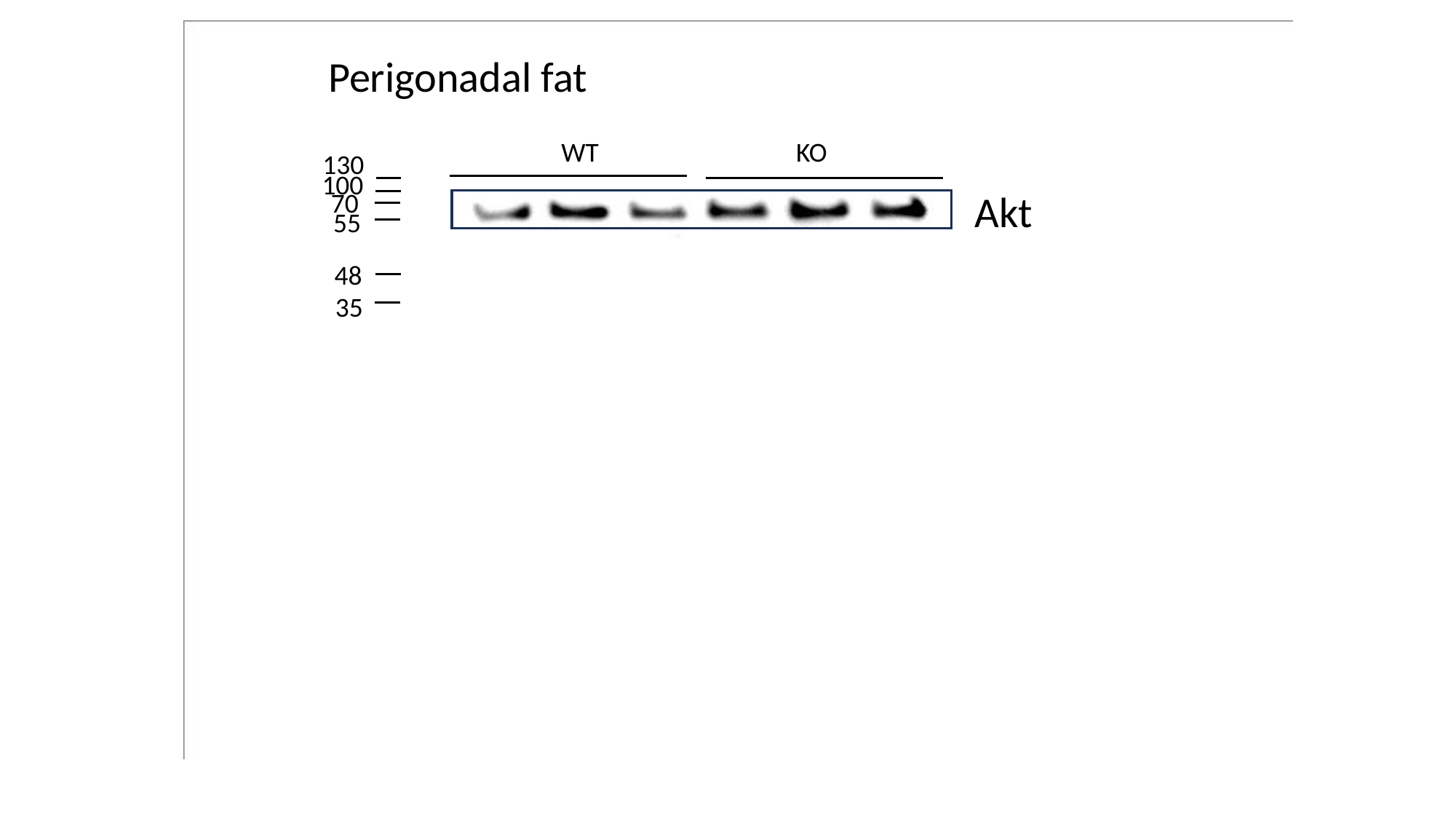

Perigonadal fat
WT KO
130
100
Akt
70
55
48
35

## Slide 3
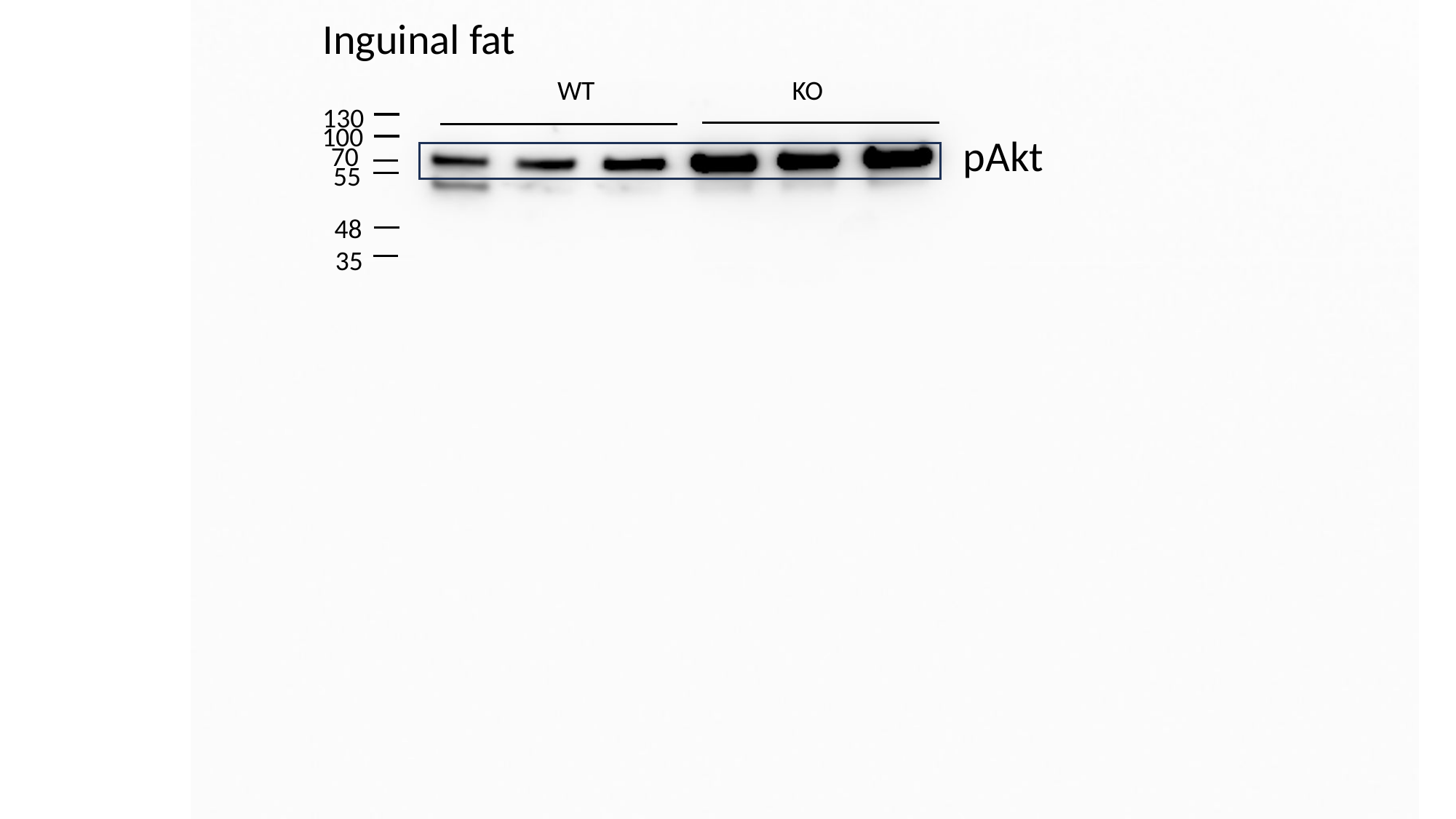

Inguinal fat
WT KO
130
100
pAkt
70
55
48
35

## Slide 4
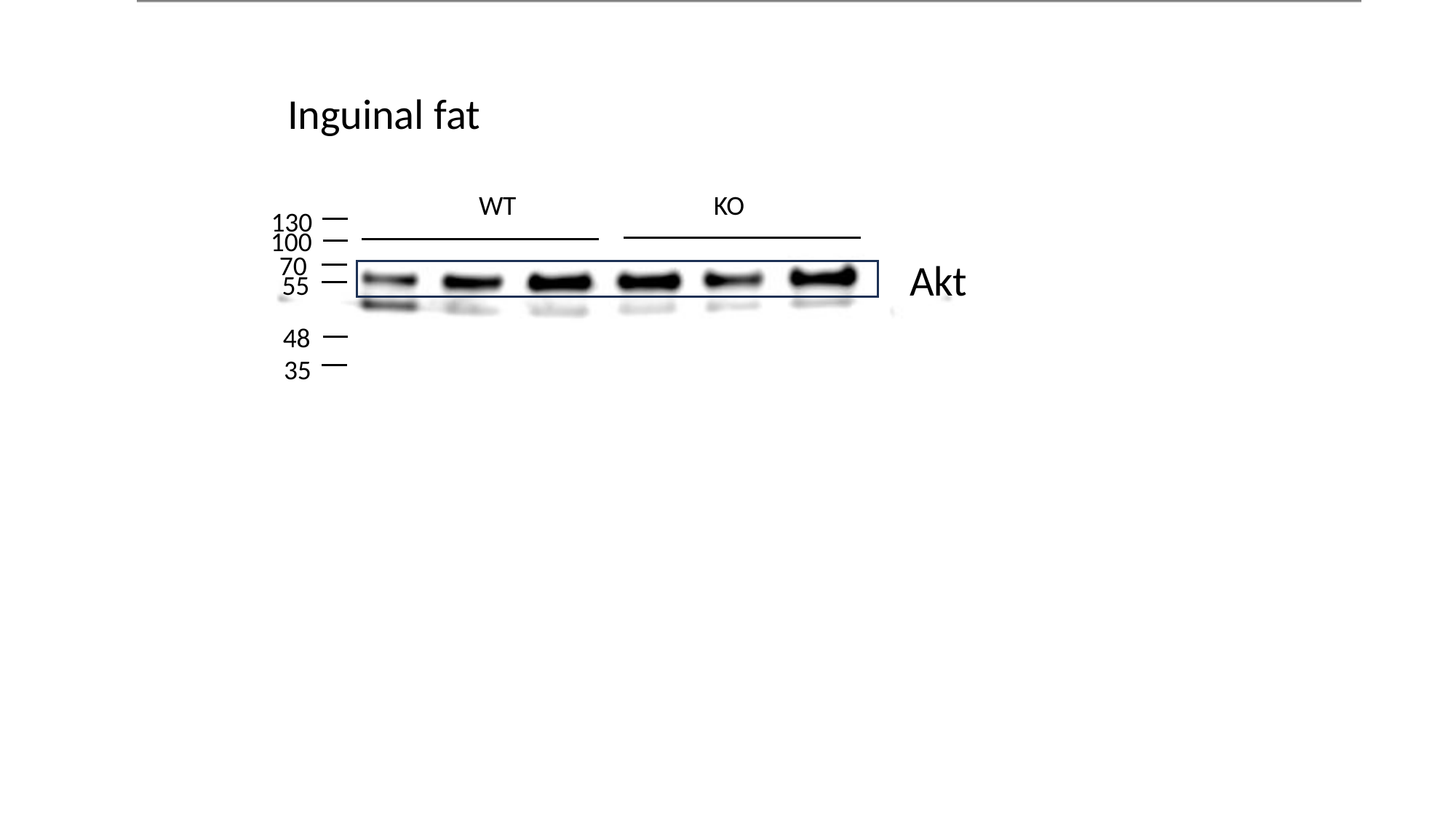

Inguinal fat
WT KO
130
100
70
Akt
55
48
35

## Slide 5
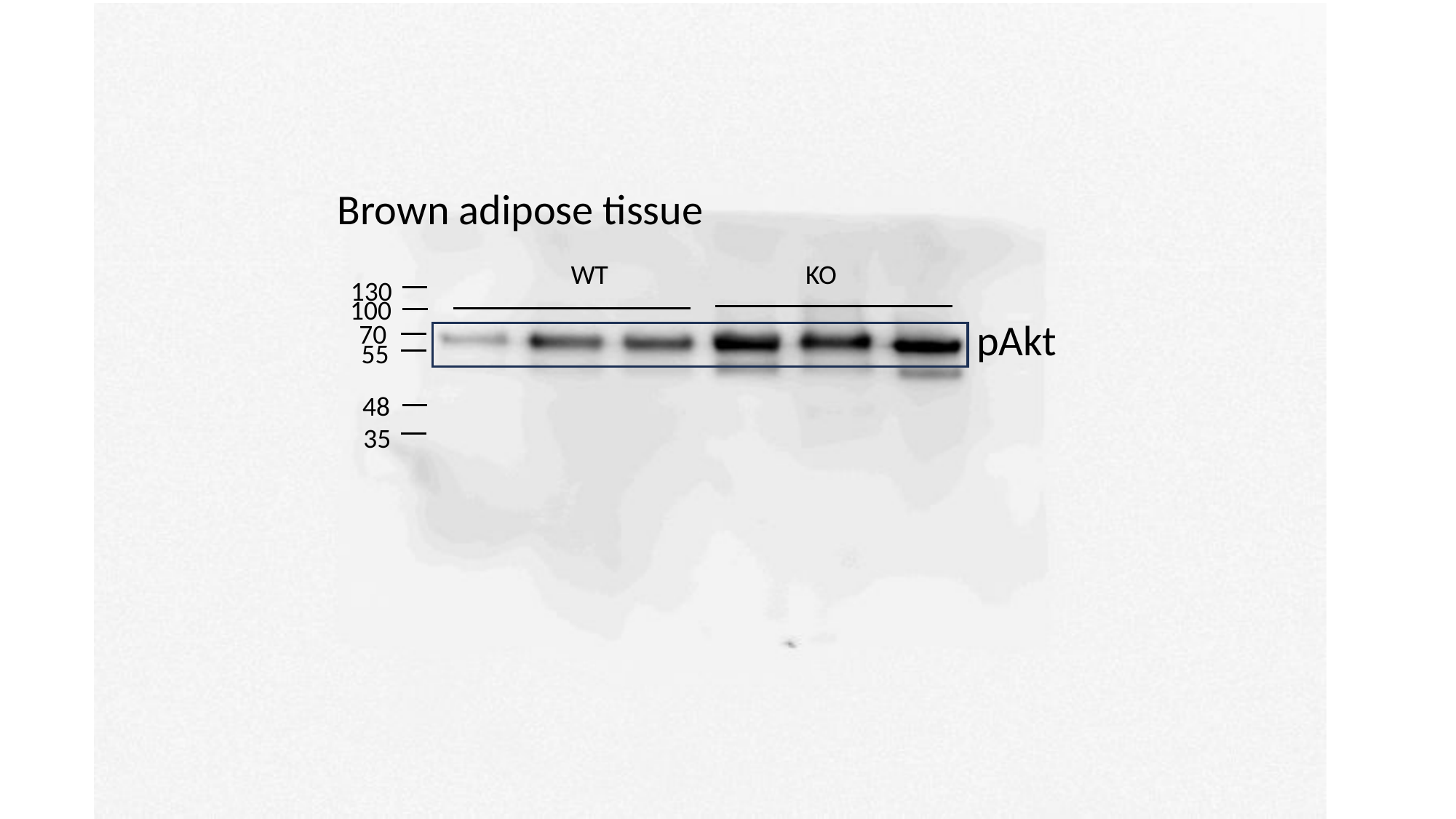

Brown adipose tissue
WT KO
130
100
pAkt
70
55
48
35

## Slide 6
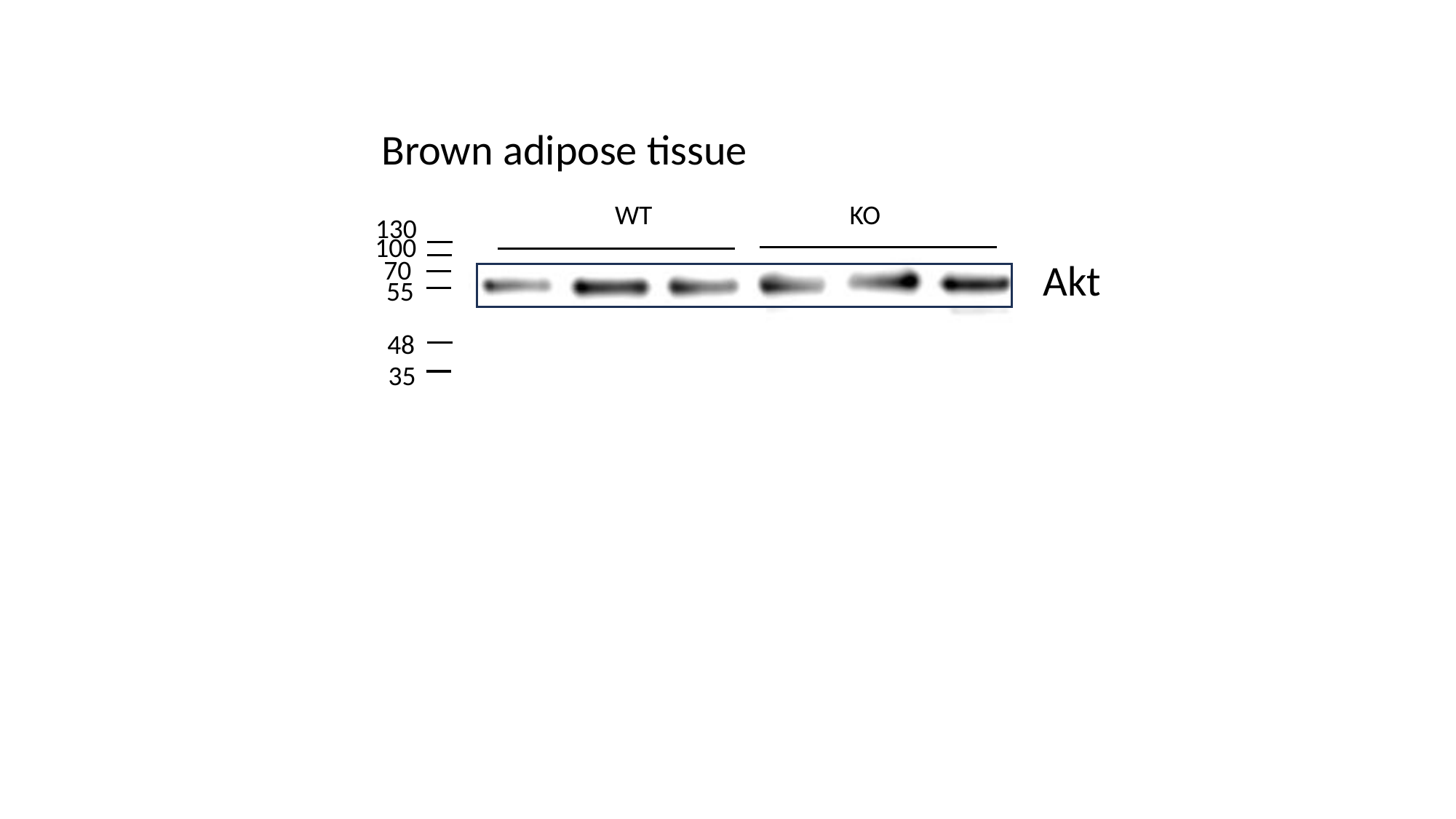

Brown adipose tissue
WT KO
130
100
70
 Akt
55
48
35

## Slide 7
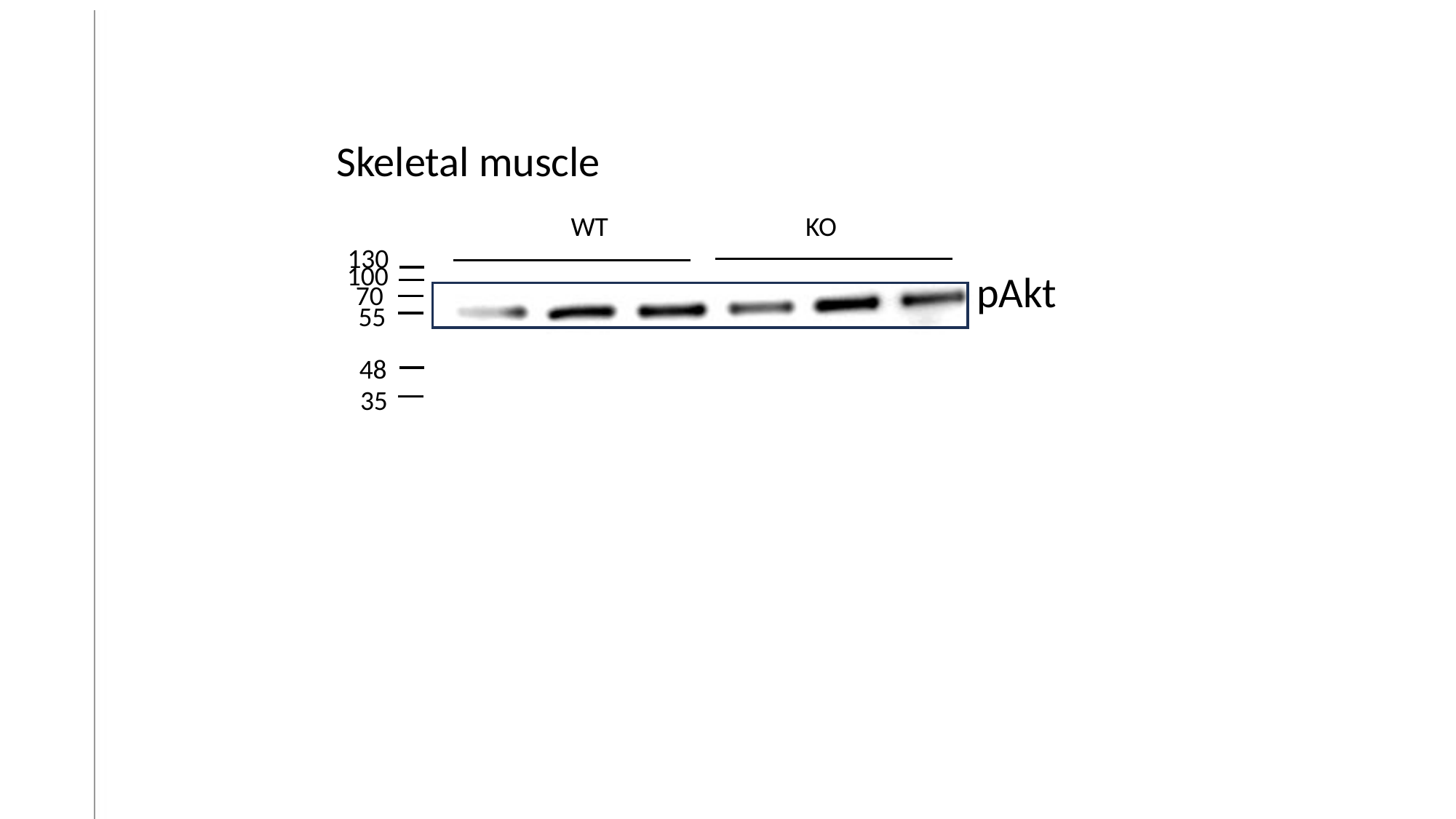

Skeletal muscle
WT KO
130
100
pAkt
70
55
48
35

## Slide 8
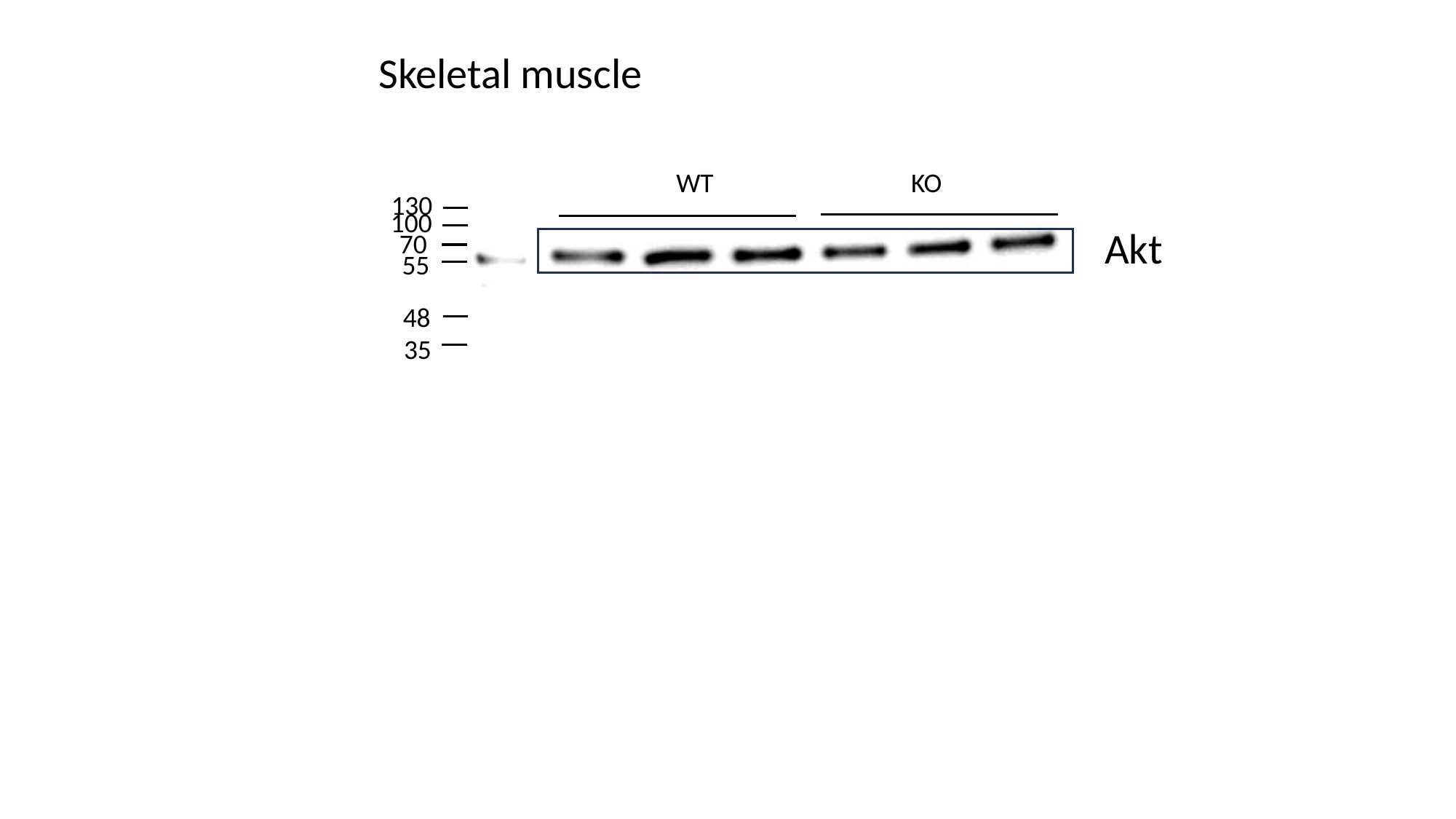

Skeletal muscle
WT KO
130
100
 Akt
70
55
48
35

## Slide 9
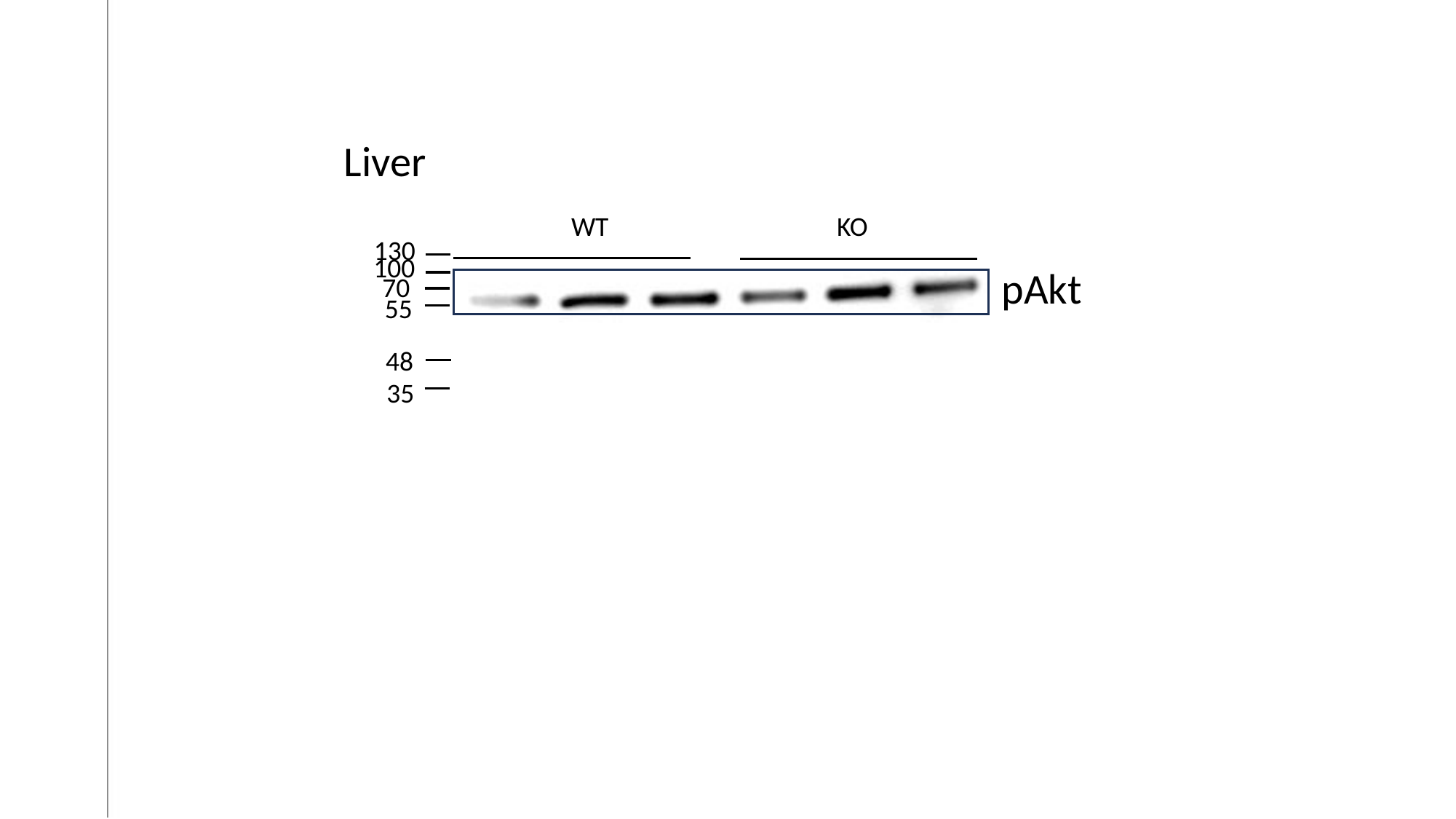

Liver
WT KO
130
100
pAkt
70
55
48
35

## Slide 10
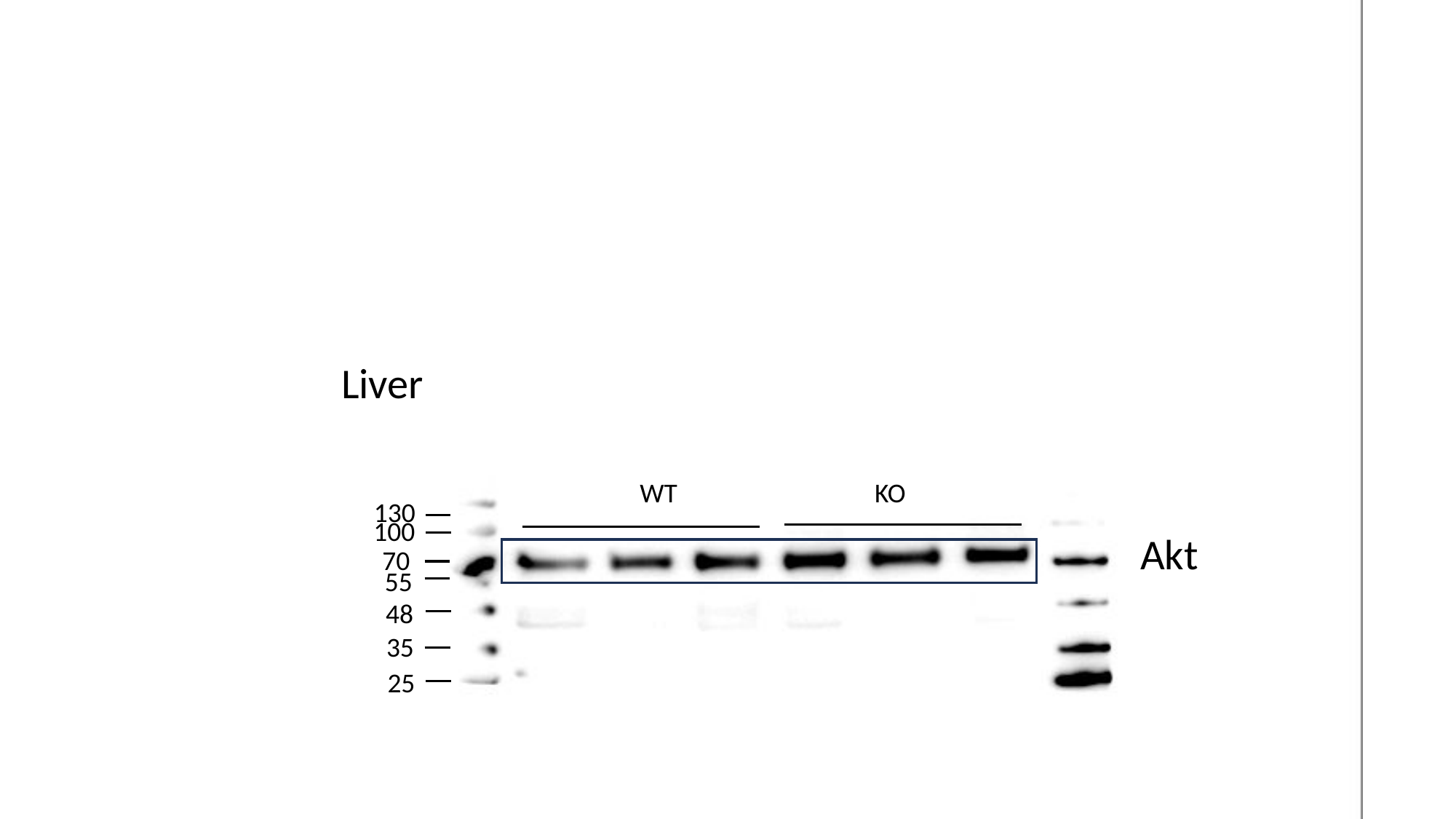

Liver
WT KO
130
100
 Akt
70
55
48
35
25
