# Supplementary material for: Identification of PTGR2 inhibitors as a new therapeutic strategy for diabetes and obesity
Source: EMBO Mol Med. 2025 Mar 21;17(5):938–66. doi: 10.1038/s44321-025-00216-4 (PMC12081876; doi:10.1038/s44321-025-00216-4)
Supplement: Supplementary file 5 — Source data Fig. 3 [file 44321_2025_216_MOESM5_ESM.zip › Figure 3/Figure 3G/Figure 3G.pptx]

## Slide 1
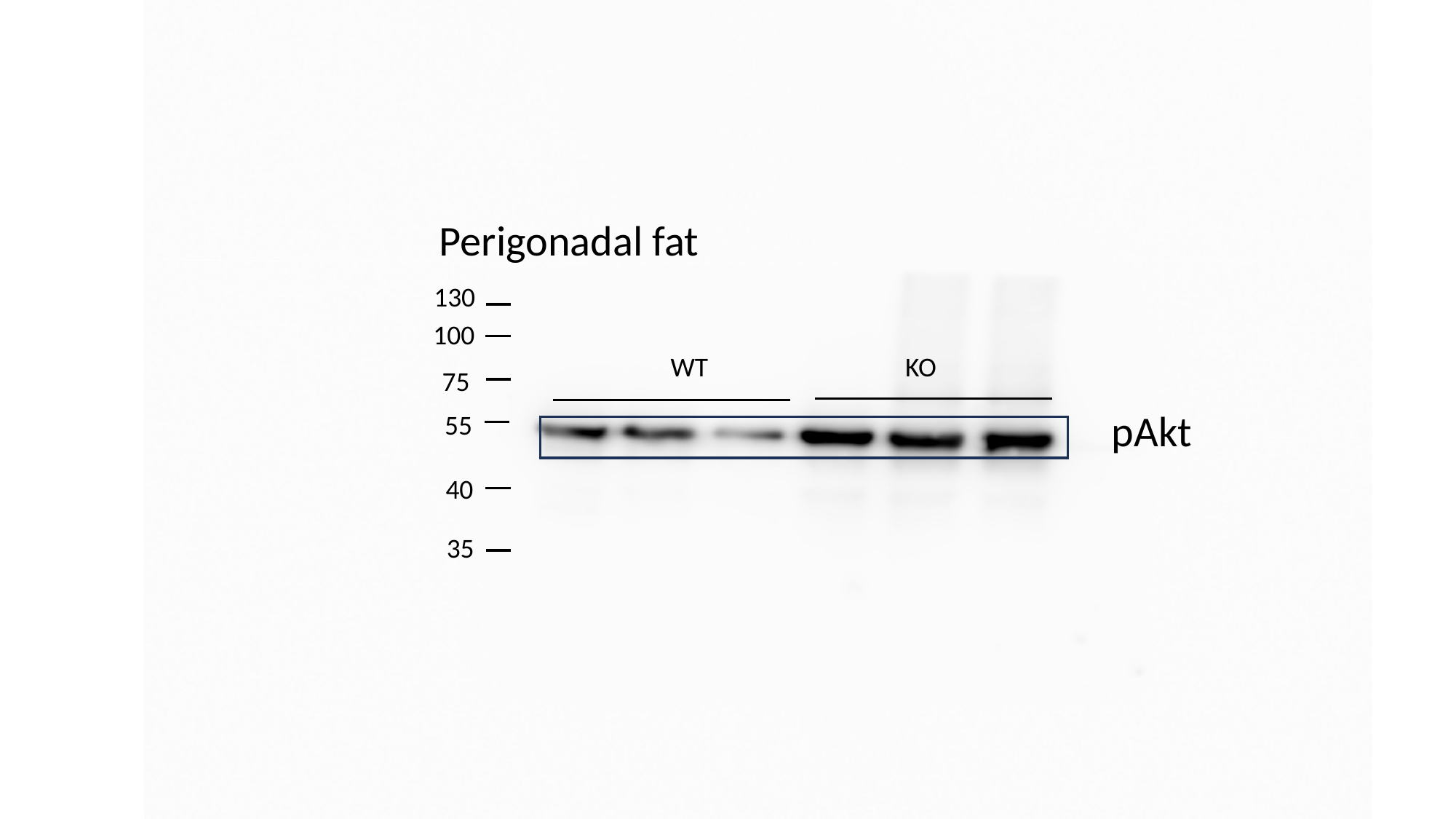

Perigonadal fat
130
100
WT KO
75
pAkt
55
40
35

## Slide 2
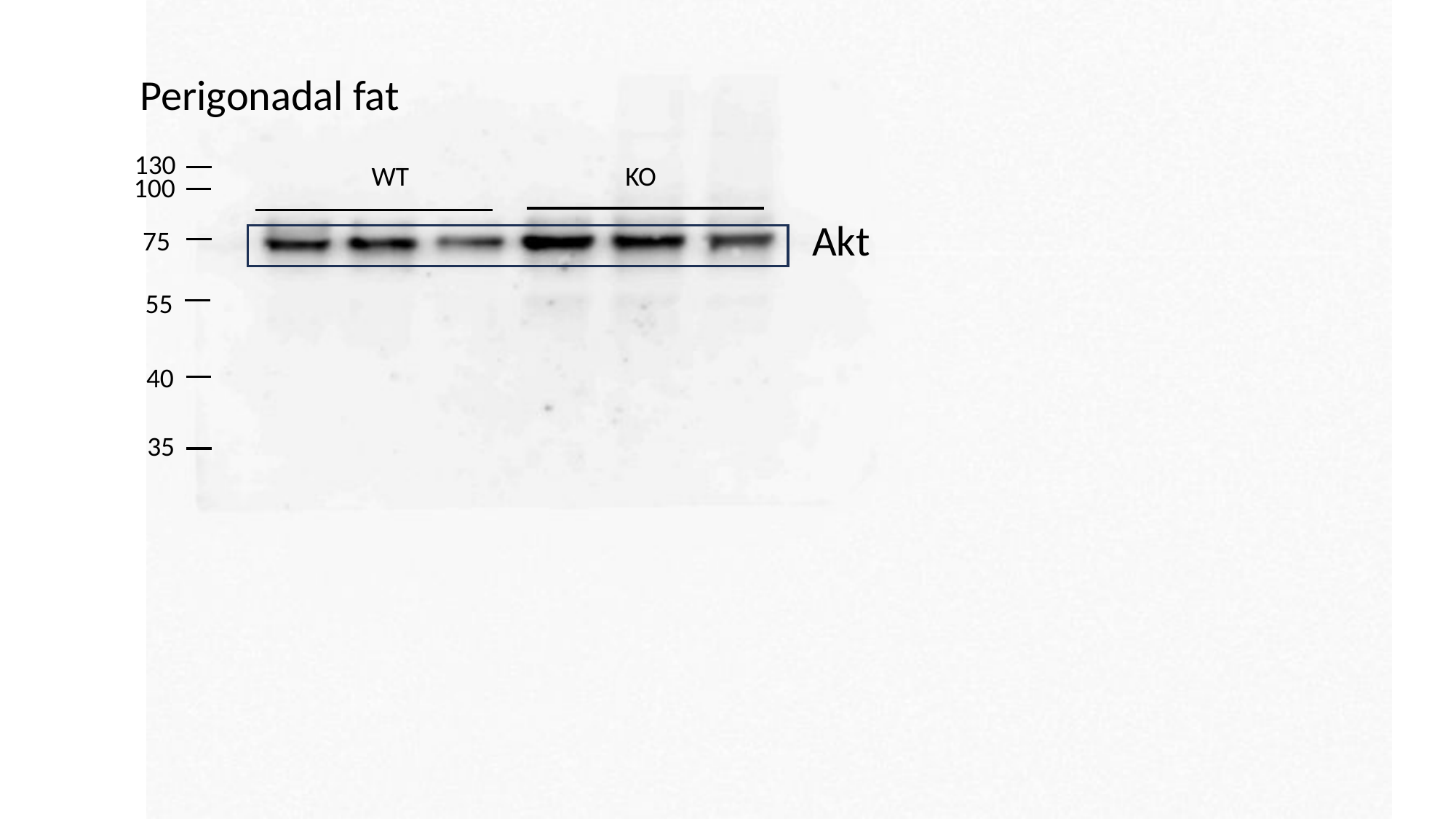

Perigonadal fat
130
WT KO
100
Akt
75
55
40
35

## Slide 3
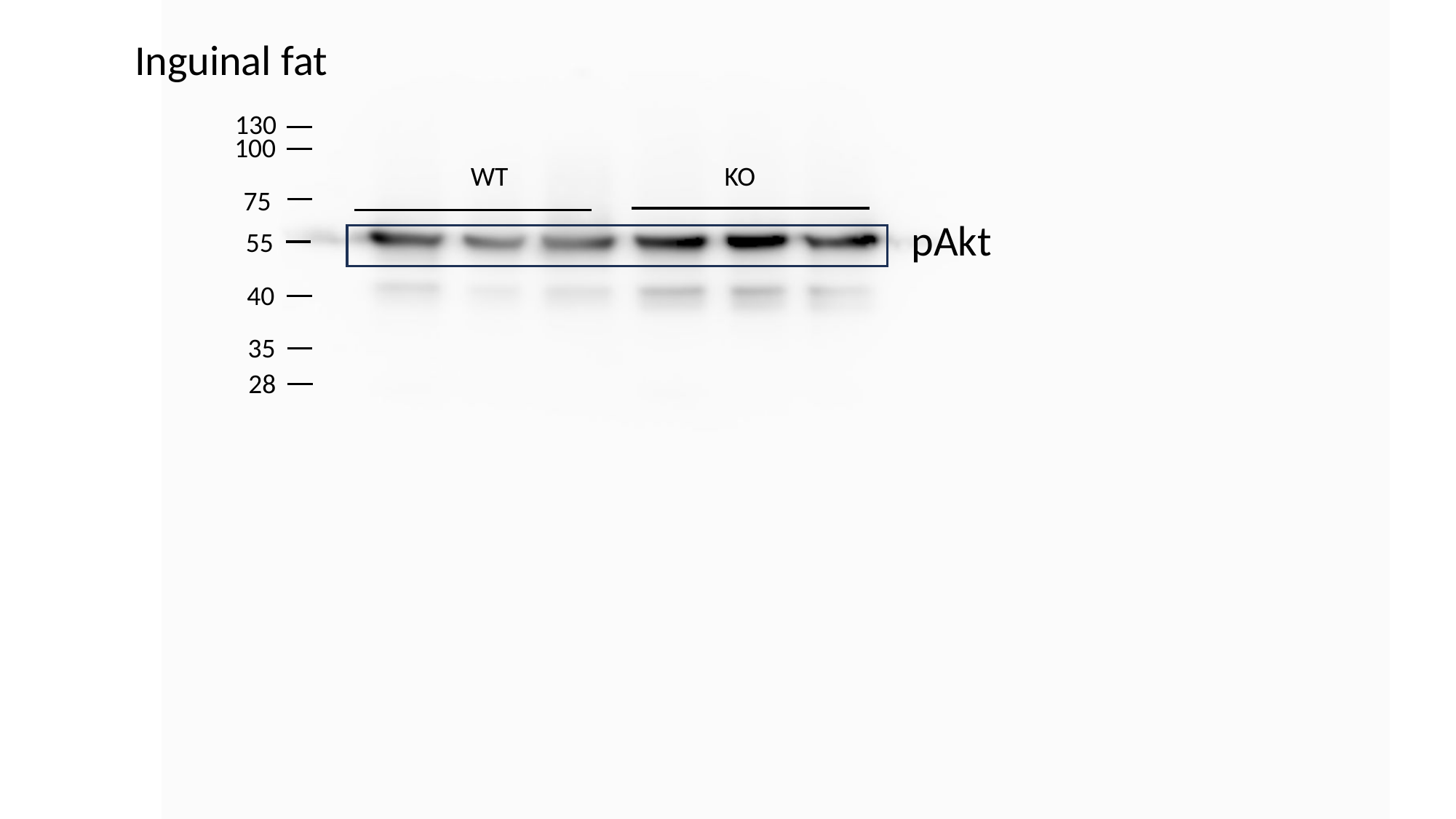

Inguinal fat
130
100
WT KO
75
pAkt
55
40
35
28

## Slide 4
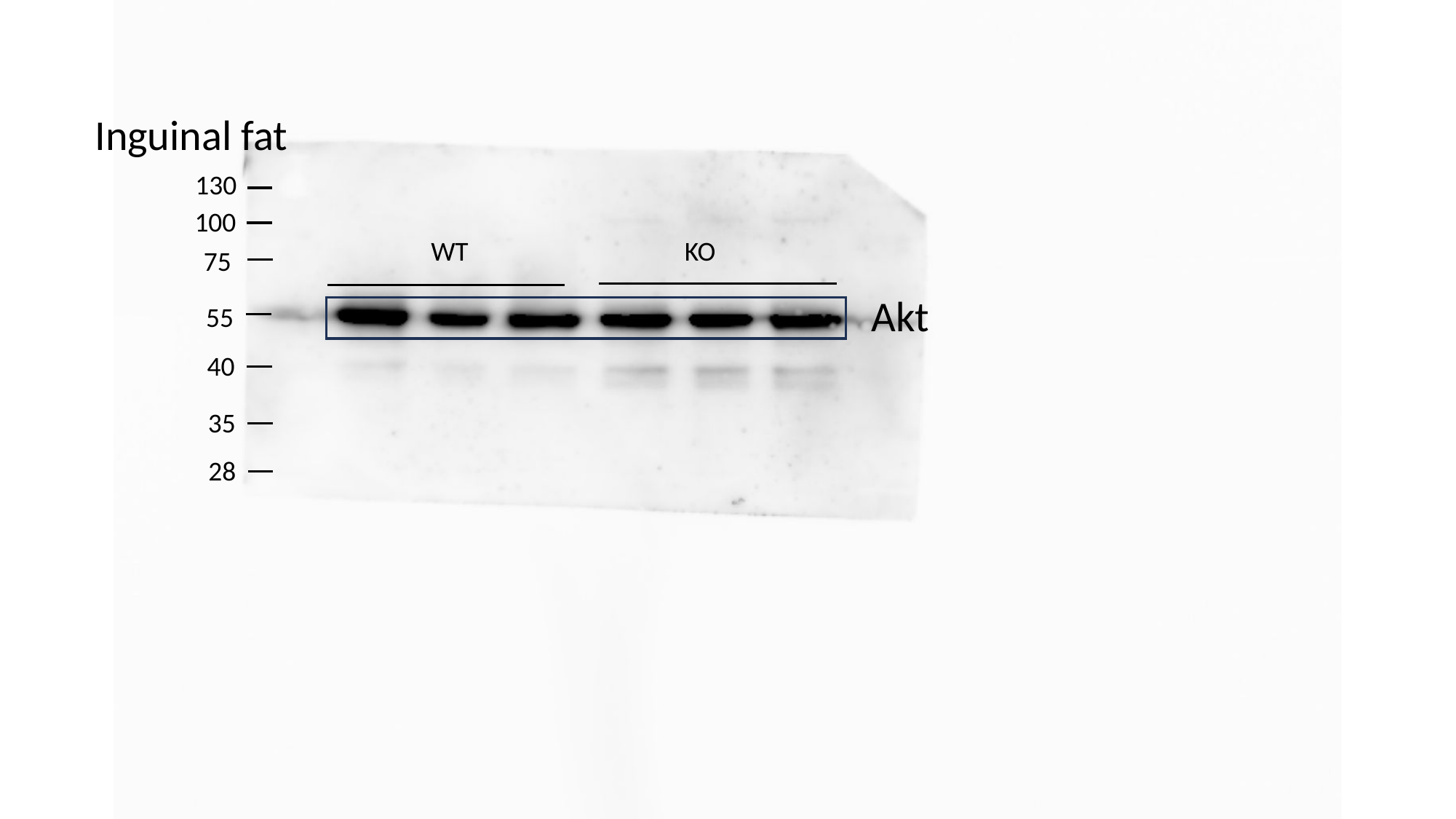

Inguinal fat
130
100
WT KO
75
Akt
55
40
35
28

## Slide 5
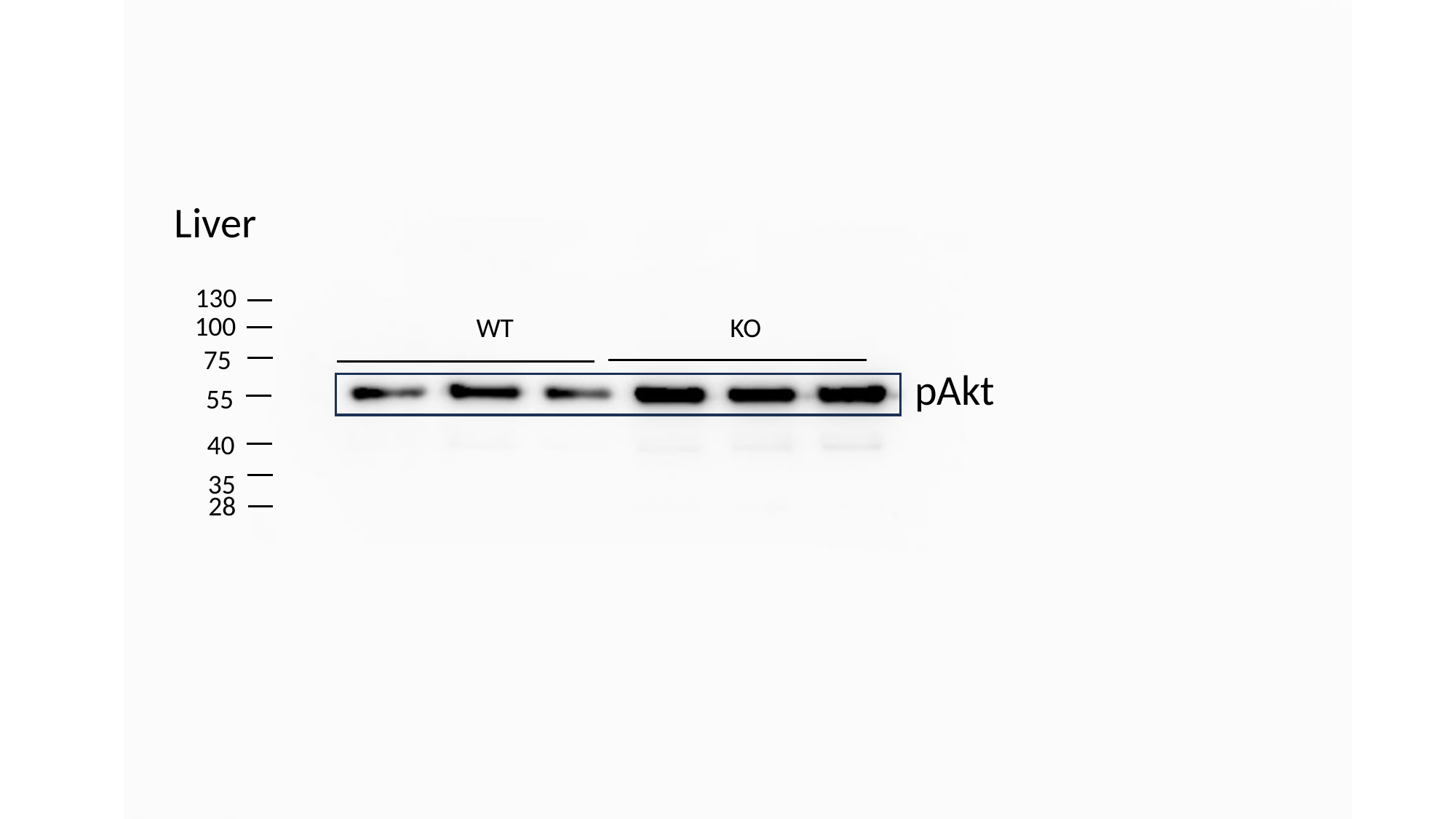

Liver
130
100
WT KO
75
pAkt
55
40
35
28

## Slide 6
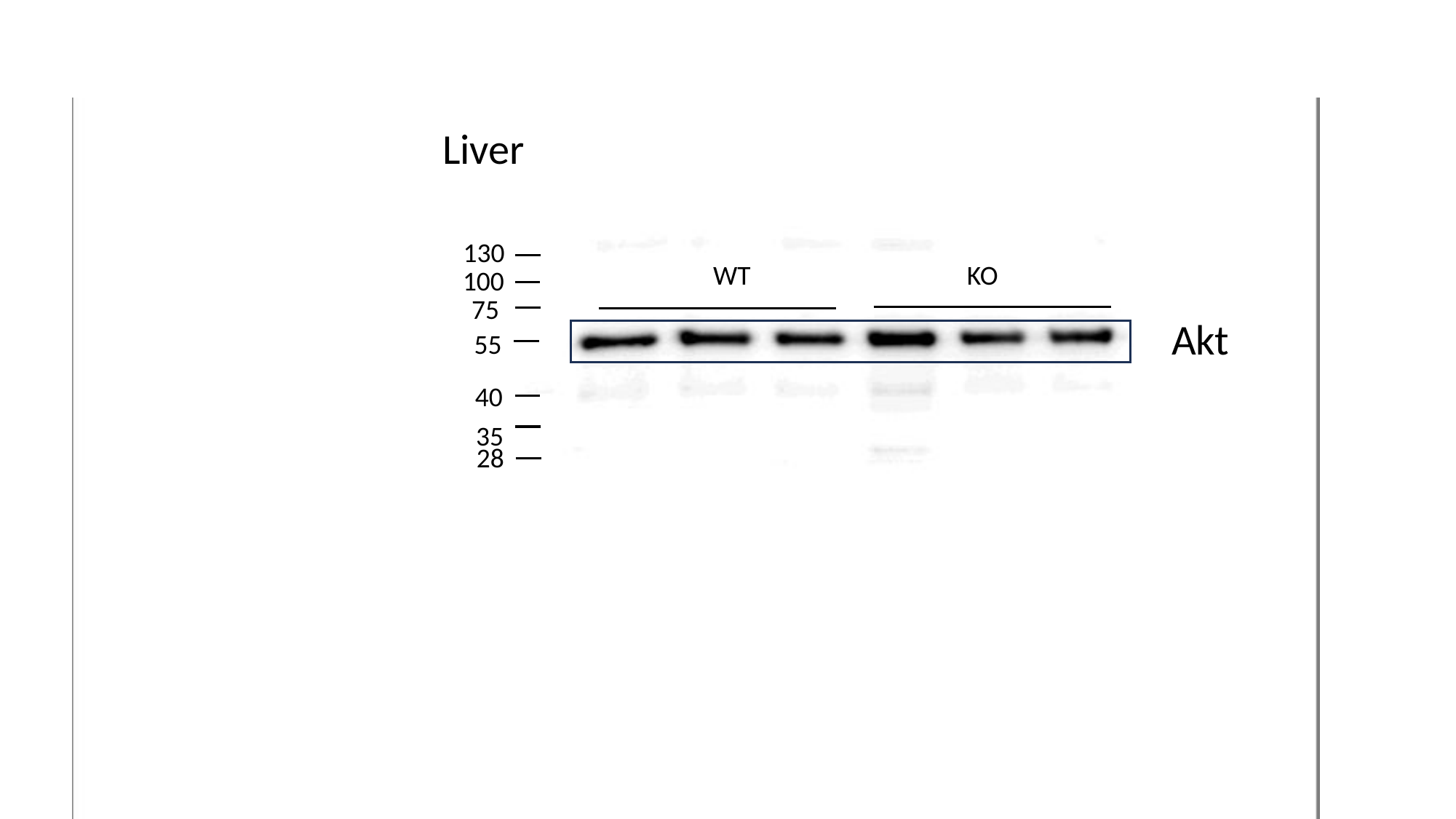

Liver
130
 WT KO
100
75
Akt
55
40
35
28

## Slide 7
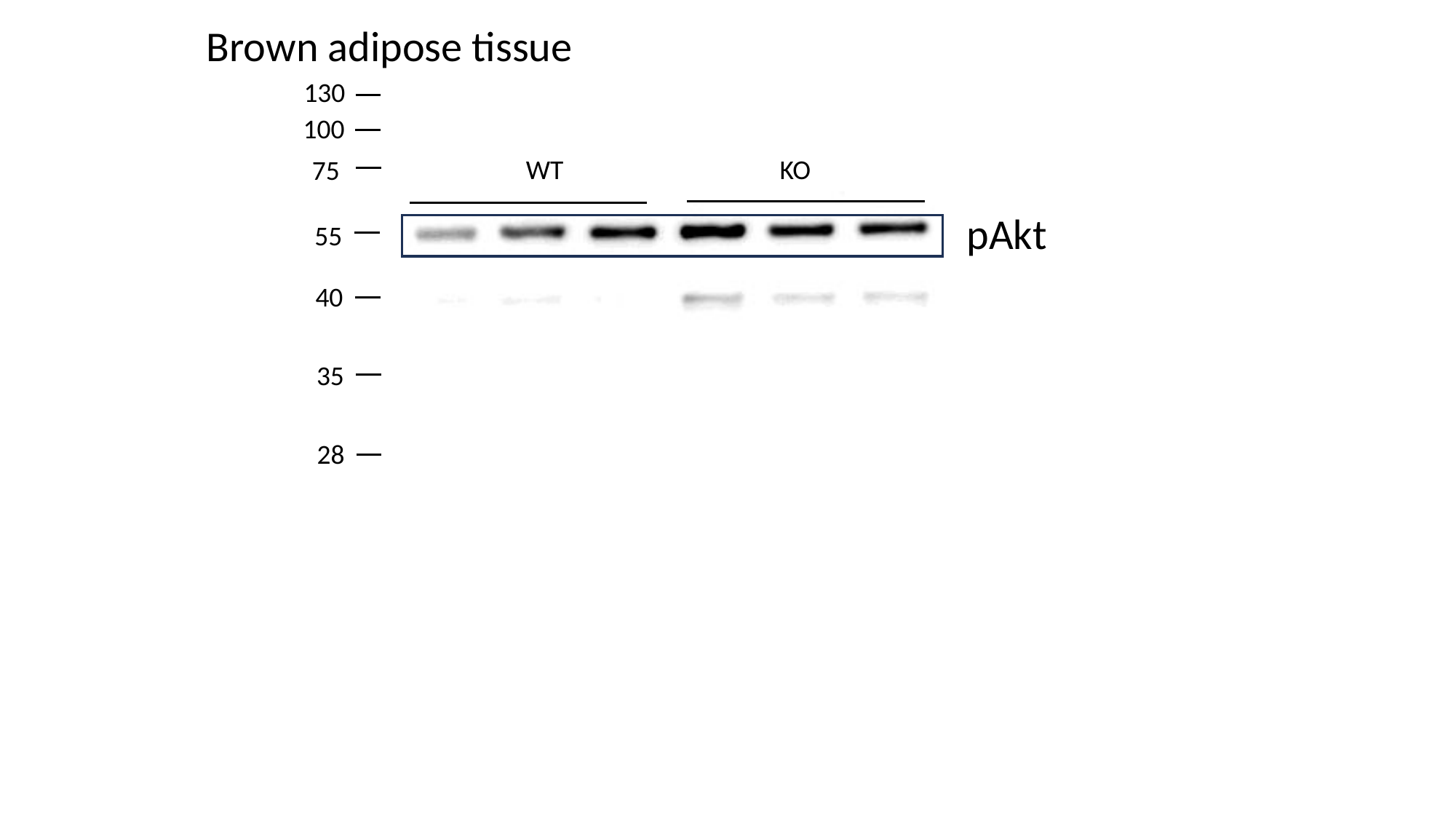

Brown adipose tissue
130
100
WT KO
75
pAkt
55
40
35
28

## Slide 8
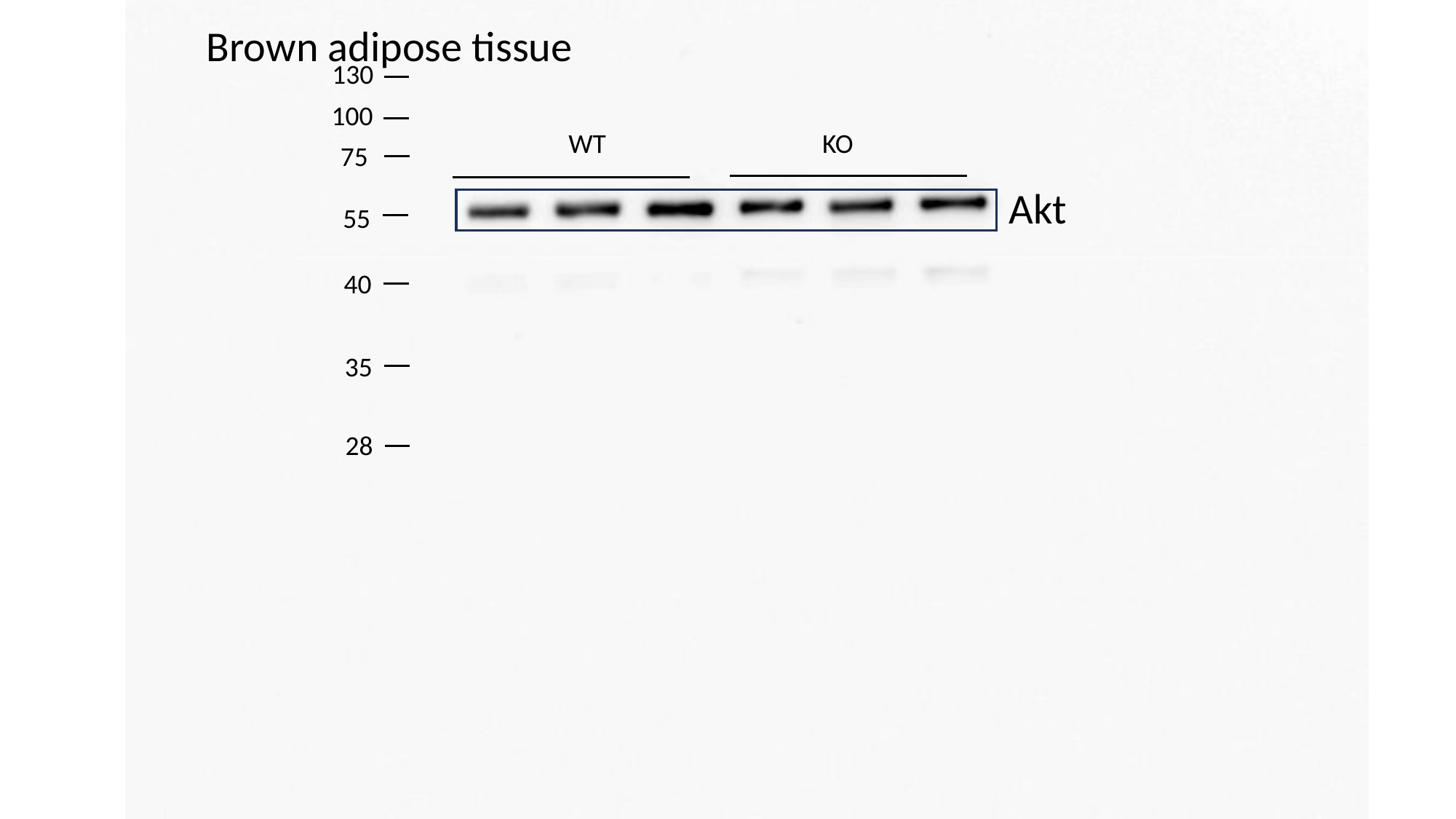

Brown adipose tissue
130
100
WT KO
75
Akt
55
40
35
28

## Slide 9
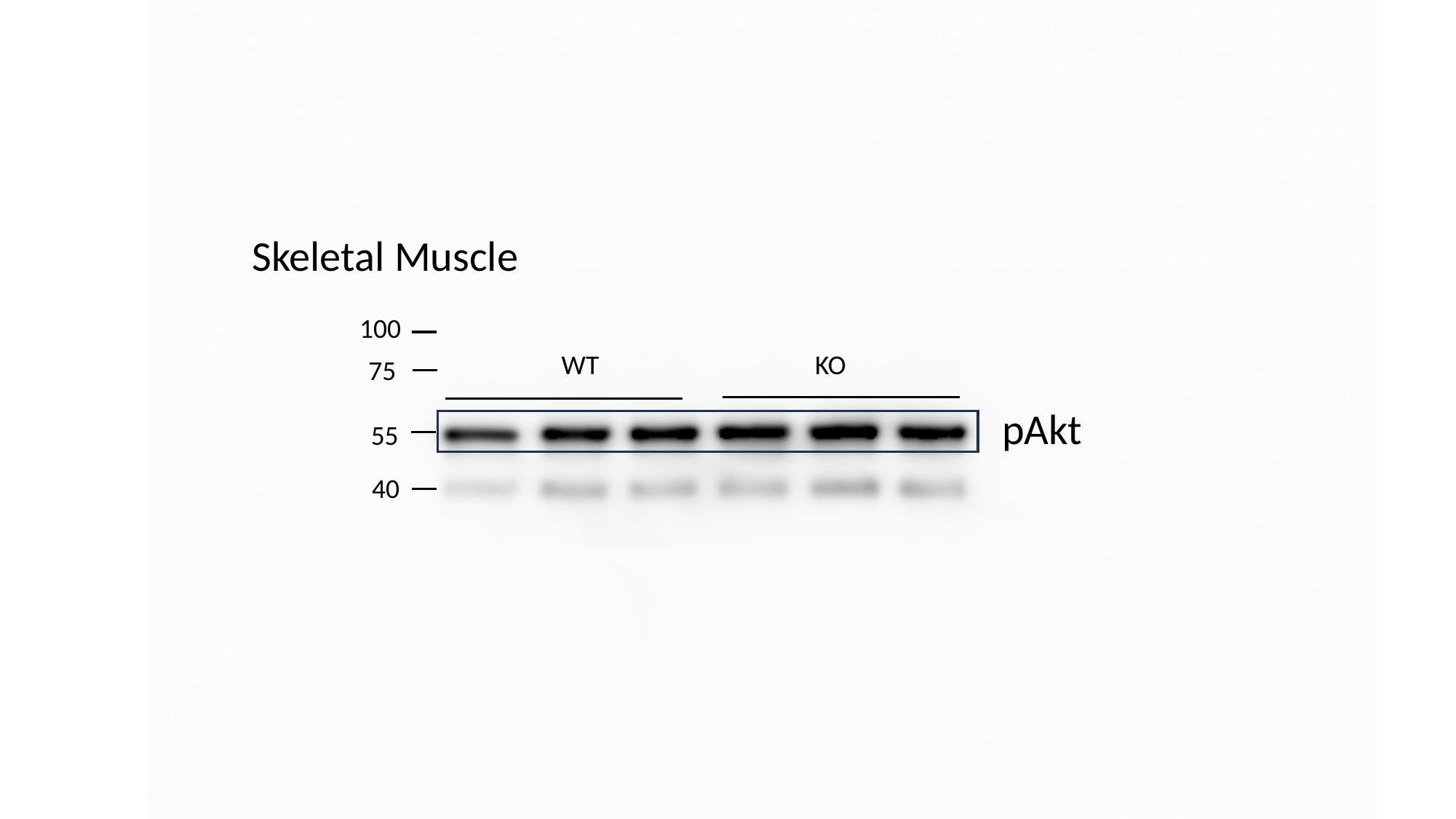

Skeletal Muscle
100
WT KO
75
pAkt
55
40

## Slide 10
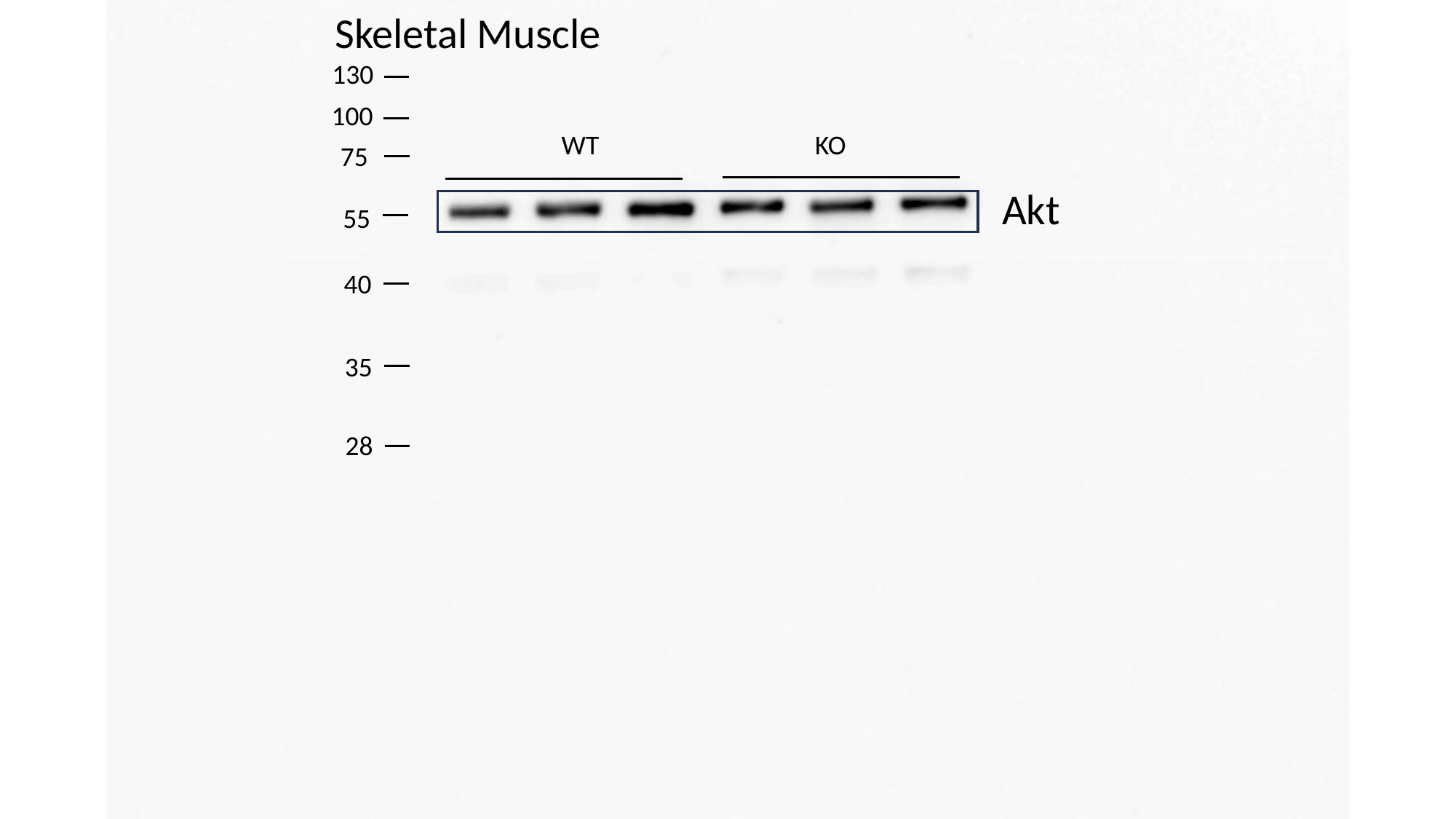

Skeletal Muscle
130
100
WT KO
75
Akt
55
40
35
28
