# Supplementary material for: Identification of PTGR2 inhibitors as a new therapeutic strategy for diabetes and obesity
Source: EMBO Mol Med. 2025 Mar 21;17(5):938–66. doi: 10.1038/s44321-025-00216-4 (PMC12081876; doi:10.1038/s44321-025-00216-4)
Supplement: Supplementary file 8 — Expanded View Figures [file 44321_2025_216_MOESM8_ESM.pdf]

Expanded View Figures

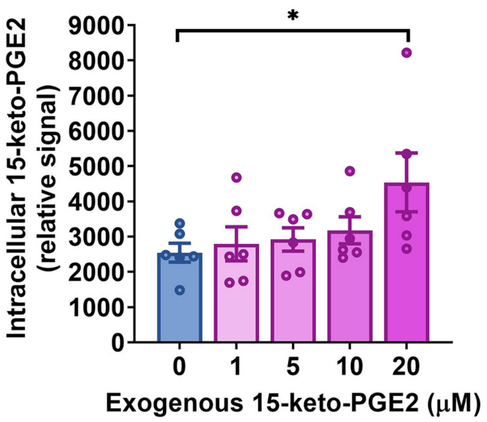

Figure EV1. Relative intracellular 15-keto-PGE2 levels in cultured 3T3-L1 cells treated with exogenous 15-keto-PGE2 of different concentrations (*$P = 0.0109$; $n = 6$ per group, 6 biological replicates with 1 technical replicate).

Data information: Data are presented as mean and standard error (S.E.M.). Statistical significance was calculated by one-way analyses of variance (ANOVA) with Tukey's post hoc test. *$P < 0.05$.

© The Author(s)

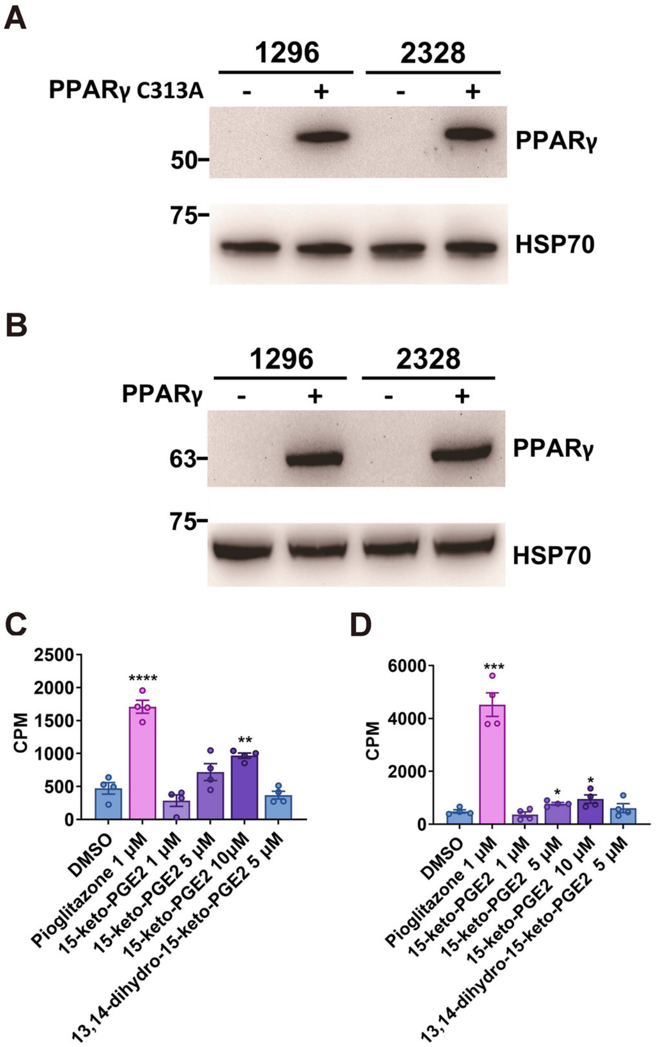

Figure EV2. 15-keto-PGE2 enhanced insulin-stimulated glucose uptake in PPARγ2-null adipocytes rescued with wild-type PPARγ2 but not with mutant PPARγ2 (C313A).

Immunoblots showing PPARγ expression in PPARγ-null 3T3-L1 clones #1296 and #2328 using the CRISPR techniques and then overexpress (**A**) mutant PPARγ2 (C313A) or (**B**) wild-type PPARγ2. 15-keto-PGE2 enhanced insulin-stimulated glucose uptake in clone #2328 rescue ($n = 4$ per cell clone, 4 biological replicates with 1 technical replicate) with (**C**) wild-type (****$P < 0.0001$, **$P = 0.0036$) or (**D**) mutant PPARγ2 (C313A) (***$P = 0.0001$, *$P = 0.0111$, *$P = 0.0363$). Data information: Data are presented as mean and standard error (S.E.M.). Statistical significance was calculated by one-way analyses of variance (ANOVA) with Tukey's post hoc test and two-sample independent *t*-test (**C**, **D**). *$P < 0.05$, **$P < 0.01$, ***$P < 0.001$, ****$P < 0.0001$.

 © The Author(s)

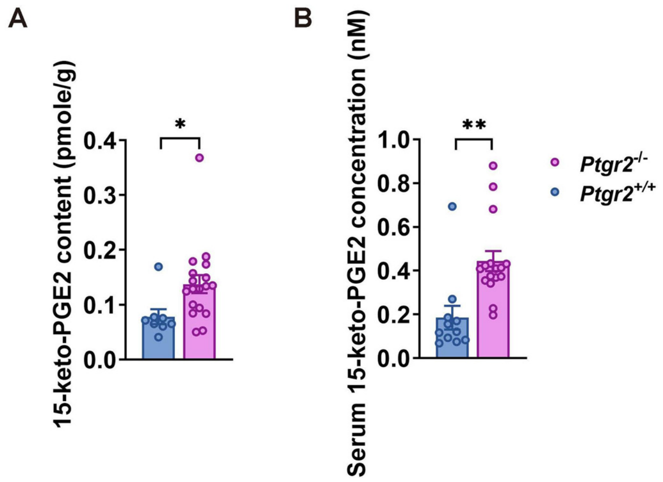

Figure EV3. Relative serum 15-keto-PGE2 concentration and 15-keto-PGE2 content in perigonadal fat were higher in *Ptgr2*$^{-/-}$ mice compared to *Ptgr2*$^{+/+}$ controls.

(**A**) Relative serum 15-keto-PGE2 level (*$P = 0.0352$) and (**B**) relative 15-keto-PGE2 content (**$P = 0.0013$) in perigonadal fat ($n = 8{:}18$ mice) of *Ptgr2*$^{-/-}$ and *Ptgr2*$^{+/+}$ mice on high-fat high-sucrose diet (HFHSD). Data information: Data are presented as mean and standard error (S.E.M.). Statistical significance was calculated by two-sample independent *t*-test in (**A**, **B**). *$P < 0.05$, **$P < 0.01$.

© The Author(s)

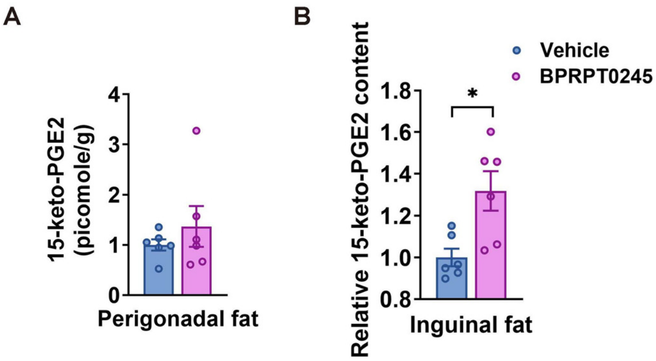

Figure EV4. Relative serum 15-keto-PGE2 concentration and 15-keto-PGE2 content in perigonadal fat were higher in mice treated with BPRPT0245 compared to those receiving the vehicle.

(**A**) Relative 15-keto-PGE2 contents in perigonadal fat ($n = 6{:}6$ mice) and (**B**) inguinal fat ($^*P = 0.0117$; $n = 6{:}6$ mice) after oral gavage of BPRPT0245 (100 mg/kg/day) for 4 days. Samples are harvested 2 h after oral gavage of the latest dose. Data information: Data are presented as mean and standard error (S.E.M.). Statistical significance was calculated by two-sample independent *t*-test (**A**, **B**). $^*P < 0.05$.

 © The Author(s)

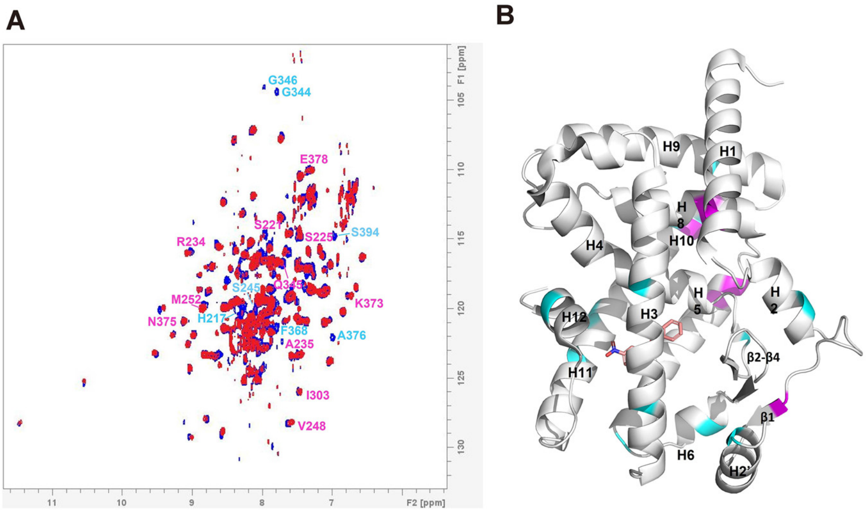

Figure EV5. Comparison between 2D [^{1}H,^{15}N]-TROSY-HSQC NMR (nuclear magnetic resonance) spectra of apo-form and 15-keto-PGE2 bound PPARγ LBD (ligand binding domain).

(**A**) Comparison between 2D [^{1}H,^{15}N]-TROSY-HSQC NMR spectra of apo-form and 15-keto-PGE2 bound PPARγ LBD (ligand binding domain). Cyanide color indicates missing peak. magenta color indicates chemical shift with $\Delta\delta > 0.05$. (**B**) NMR missing peak (cyanide color) and chemical shift (magenta color) mapped onto PPARγ LBD structure.
